# Performance of a Machine Learning Algorithm Using Electronic Health Record Data to Identify and Estimate Survival in a Longitudinal Cohort of Patients With Lung Cancer

**DOI:** 10.1001/jamanetworkopen.2021.14723

**Published:** 2021-07-07

**Authors:** Qianyu Yuan, Tianrun Cai, Chuan Hong, Mulong Du, Bruce E. Johnson, Michael Lanuti, Tianxi Cai, David C. Christiani

**Affiliations:** 1 Department of Environmental Health, Harvard T.H. Chan School of Public Health, Boston, Massachusetts; 2 Division of Rheumatology, Immunology, and Allergy, Brigham and Women’s Hospital, Boston, Massachusetts; 3 Department of Biostatistics, Harvard T.H. Chan School of Public Health, Boston, Massachusetts; 4 Department of Biomedical Informatics, Harvard Medical School, Boston, Massachusetts; 5 Department of Biostatistics, Center for Global Health, School of Public Health, Nanjing Medical University, Nanjing, China; 6 Department of Medical Oncology, Dana-Farber Cancer Institute, Boston, Massachusetts; 7 Center for Cancer Genomics, Dana-Farber Cancer Institute, Boston, Massachusetts; 8 Center for Thoracic Cancers, Division of Thoracic Surgery, Massachusetts General Hospital Cancer Center, Boston, Massachusetts; 9 Department of Medicine, Massachusetts General Hospital/Harvard Medical School, Boston, Massachusetts

## Abstract

**Importance:**

Electronic health records (EHRs) provide a low-cost means of accessing detailed longitudinal clinical data for large populations. A lung cancer cohort assembled from EHR data would be a powerful platform for clinical outcome studies.

**Objective:**

To investigate whether a clinical cohort assembled from EHRs could be used in a lung cancer prognosis study.

**Design, Setting, and Participants:**

In this cohort study, patients with lung cancer were identified among 76 643 patients with at least 1 lung cancer diagnostic code deposited in an EHR in Mass General Brigham health care system from July 1988 to October 2018. Patients were identified via a semisupervised machine learning algorithm, for which clinical information was extracted from structured and unstructured data via natural language processing tools. Data completeness and accuracy were assessed by comparing with the Boston Lung Cancer Study and against criterion standard EHR review results. A prognostic model for non–small cell lung cancer (NSCLC) overall survival was further developed for clinical application. Data were analyzed from March 2019 through July 2020.

**Exposures:**

Clinical data deposited in EHRs for cohort construction and variables of interest for the prognostic model were collected.

**Main Outcomes and Measures:**

The primary outcomes were the performance of the lung cancer classification model and the quality of the extracted variables; the secondary outcome was the performance of the prognostic model.

**Results:**

Among 76 643 patients with at least 1 lung cancer diagnostic code, 42 069 patients were identified as having lung cancer, with a positive predictive value of 94.4%. The study cohort consisted of 35 375 patients (16 613 men [47.0%] and 18 756 women [53.0%]; 30 140 White individuals [85.2%], 1040 Black individuals [2.9%], and 857 Asian individuals [2.4%]) after excluding patients with lung cancer history and less than 14 days of follow-up after initial diagnosis. The median (interquartile range) age at diagnosis was 66.7 (58.4-74.1) years. The area under the receiver operating characteristic curves of the prognostic model for overall survival with NSCLC were 0.828 (95% CI, 0.815-0.842) for 1-year prediction, 0.825 (95% CI, 0.812-0.836) for 2-year prediction, 0.814 (95% CI, 0.800-0.826) for 3-year prediction, 0.814 (95% CI, 0.799-0.828) for 4-year prediction, and 0.812 (95% CI, 0.798-0.825) for 5-year prediction.

**Conclusions and Relevance:**

These findings suggest the feasibility of assembling a large-scale EHR-based lung cancer cohort with detailed longitudinal clinical measurements and that EHR data may be applied in cancer progression with a set of generalizable approaches.

## Key Points

**Question:**

Can electronic health record (EHR) elements be integrated to assemble a lung cancer cohort to study prognosis?

**Findings:**

In this cohort study among 42 069 individuals with lung cancer, key cancer characteristics were extracted from structured data and narrative notes by developing customized natural language processing (NLP) tools using EHRs. The prognostic model based on this cohort may estimate overall survival for non–small cell lung cancer with good discrimination.

**Meaning:**

These finding suggest that with well-designed strategies involving machine learning, NLP, and quality assessment, EHR data may be used for cancer research.

## Teaser

This cohort study investigates the performance of a machine learning algorithm used to extract a cohort of patients with lung cancer from electronic health records and estimate overall survival.

## Introduction

Globally, lung cancer has been the most commonly diagnosed cancer and leading cause of cancer-related deaths for several decades (not counting skin cancer).^1^ In the United States, the current 5-year survival rate is approximately 20.6%.^2^ Patients with lung cancer have different outcomes based on various clinical factors.^3,4,5,6^ A 2020 study^7^ using data from Surveillance, Epidemiology, and End Results (SEER) found a significant reduction in mortality for lung cancer from 2013 to 2016, which was potentially associated with incidence reduction along with treatment advances. A large cohort with adequate clinical information is necessary to identify stable and reliable prognostic variables and the factors associated with improved survival outcomes.

The growing availability of access to electronic health record (EHR) data offers a timely and low-cost alternative to traditional cohort studies, with the potential of efficiently including broad and large real-world populations.^8,9^ However, the inconsistent coding and the diversity and complexity of EHR data also introduce difficulties in obtaining research-quality cancer-related data. Many data elements are typically recorded as free text with different terms, which make natural language processing (NLP) a requisite technology for accurate data extraction and classification.^10,11^ In particular, many clinical variables, such as lung cancer status, are not explicitly represented in EHRs but can be inferred based on multiple data elements via machine learning algorithms.^12,13,14^

Our primary goal was to build a large and reliable lung cancer EHR cohort that could be used for studying lung cancer progression with a set of generalizable approaches. To this end, we combined structured data and unstructured data to identify patients with lung cancer and extract clinical variables. We evaluated the completeness and accuracy of the extracted data. To further illustrate the application of EHR cohort data, we developed and validated a prognostic model to predict 1-year to 5-year overall survival (OS) among individuals with non–small cell lung cancer (NSCLC).

## Methods

The institutional review board of Mass General Brigham (MGB) health care (protocol No. 1999P004935/PHS) approved this cohort study and the release of data, which were collected after acquiring written informed consent from participants. This study is reported following the Strengthening the Reporting of Observational Studies in Epidemiology (STROBE) reporting guideline.

### Data Source and Study Population

**Figure 1.**
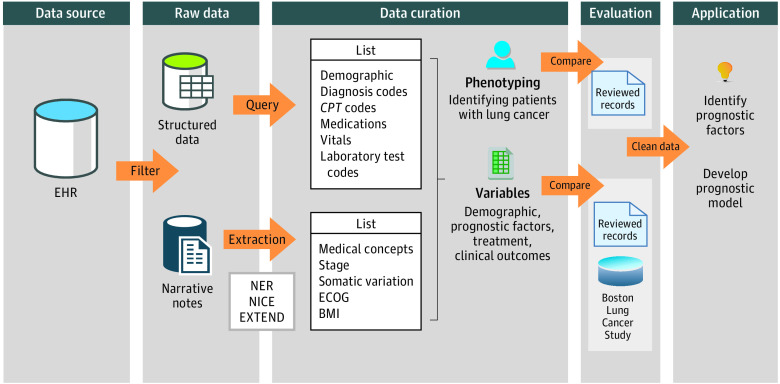
Overview of Electronic Health Record (EHR) Cohort Assembling Data were initially from EHRs; lung cancer diagnosis code was used as a filter to create a data mart containing structured data and narrative notes. Structured data were queried, and narrative notes were processed using natural language processing tools. Structured data and narrative notes were combined to develop the phenotyping algorithm and extract variables of interest. The performance of the phenotyping algorithm was compared with a random sample of patients selected for EHR review. The accuracies of the extracted variables were compared with EHR reviewed samples and Boston Lung Cancer Study cohort data. BMI indicates body mass index; *CPT*, *Current Procedural Terminology*; ECOG, Eastern Cooperative Oncology Group; EXTEND, Extraction of Electronic Medical Record Numerical Data; NER, named-entity recognition; NICE, Natural Language Processing Interpreter for Cancer Extraction.

The overview of cohort assembly from EHRs is shown in Figure 1. The initial data mart consisted of 76 643 patients with at least 1 *International Classification of Diseases, Ninth Revision (ICD-9)* or *International Statistical Classification of Diseases and Related Health Problems, Tenth Revision (ICD-10)* code for lung cancer (eAppendix in the Supplement). All EHR data for these patients were extracted from Massachusetts General Hospital (MGH) and Brigham and Women’s Hospital using the MGB health care system Research Patient Data Registry.

The Boston Lung Cancer Study (BLCS) is a cancer epidemiology cohort of patients with lung cancer enrolled at MGH and Dana-Farber Cancer Institute from 1992 to the present. A total of 6225 patients from MGH linked to the EHR database were used for data comparison in our study. The BLCS collects information on cancer diagnosis date, stage, histologic type, treatment, and clinical outcomes.

### Identification of Patients With Lung Cancer

#### Training Labels

To develop the classification algorithm to identify patients with lung cancer, 200 individuals were randomly selected from the initial data mart as the criterion set. Medical record reviews of 200 EHRs were separately performed by 2 reviewers (including QY). Discrepancies were resolved via additional in-depth EHR review yielding a consensus opinion on the data element. Among 200 patients with lung cancer *ICD-9* or *ICD-10* codes, we identified 142 patients with definite primary lung cancer diagnoses, 55 patients without lung cancer, and 3 patients with uncertain diagnoses.

#### Algorithm Training and Evaluation

We used the established phenotyping method High-Throughput Phenotyping with EHR using a Common Automated Pipeline (PheCAP) version 1.2.1 (Predictive Analytics Research Solution and Execution)*,* to develop and evaluate an algorithm for classifying lung cancer status.^13^ The PheCAP method includes 3 key steps: feature extraction based on the Surrogate-Assisted Feature Extraction (SAFE) algorithm, algorithm development based on penalized regression, and algorithm validation to evaluate accuracy of the algorithm. The initial feature data for PheCAP consisted of counts of a list of codified features identified by domain experts, NLP features identified from online knowledge source articles as suggested in SAFE, and a health care use feature measured by the total counts of medical notes.^13^

We performed SAFE to select a subset of informative features via sparse regression against the lung cancer *ICD-9* or *ICD-10* and NLP counts, which served as noisy labels of true lung cancer status. We developed a classification algorithm by fitting least absolute shrinkage and selection operator–penalized logistic regression to the training data on SAFE-selected features and criterion labels. The algorithm assigned each patient a probability of having lung cancer. Those with probabilities greater than a threshold that achieved 90% specificity were classified as having lung cancer. Comparing against EHR-review criterion labels, performance characteristics were reported using the area under the receiver operating characteristic curve (AUROC), as well as positive predictive value (PPV), sensitivity, specificity, and *F* score. Cross-validation with 70:30 splits for which means were found over 100 random partitions was used to obtain a bias-corrected estimate of these accuracy parameters.

### Extraction of Clinical Variables for Lung Cancer Prognosis

In addition to demographics and clinical outcomes, we obtained prognostic factors and treatment information from structured data and clinical notes using NLP tools (Table 1). We used the published tool Extraction of Electronic Medical Record Numerical Data^15^ to extract Eastern Cooperative Oncology Group (ECOG) Scale of Performance Status and body mass index (BMI; calculated as weight in kilograms divided by height in meters squared) information. We further developed the NLP Interpreter for Cancer Extraction (NICE) tool to infer cancer characteristics, including stage, histologic type, diagnosis date, and somatic variant information from clinical notes, including pathology reports, discharge summaries, and progress notes (eFigure 1 in the Supplement). Smoking status was predicted by a classification algorithm (eTable 1 in the Supplement) The detailed processes for determining the value of each variable are described in the eAppendix in the Supplement. Dictionaries for NLP tools are shown in eTable 2 in the Supplement.

**Table 1.** Data Sources, Extraction Method, and Description

Key variable	Data sources and extraction method		Variable description
	Structured data	Unstructured data	
Demographic characteristic			
Birth date	Demographics	NA	NA
Sex	Demographics	NA	NA
Race/ethnicity	Demographics	NA	NA
Clinical outcomes			
Diagnosis date	Diagnosis codes (*ICD-9* and *ICD-10* codes)	NICE	Date of lung cancer diagnosis
Date of death	Death report	NA	NA
Prognostic factors	NA	NA	NA
Stage	NA	NICE	TNM stage and clinical stage
Histologic type	NA	NICE	NSCLC (ie, adenocarcinoma, squamous cell carcinoma, other non-small cell carcinoma) or small cell lung cancer
Smoking status	NA	NA	Smoker or nonsmoker
BMI	Vital signs	EXTEND	Calculated as weight in kilograms divided by height in meters squared
ECOG performance status	NA	EXTEND	Grade 0 to 4
Laboratory test	Laboratory test codes	NA	Complete blood count, metabolic panel, lipid panel, liver panel, hemoglobin A1C, and urinalysis
Tumor somatic variant information	NA	NICE	Genetic alterations in *EGFR*, *KRAS*, *ALK*, *ROS1*, *MET*, or *BRAF*
Medical history	Diagnosis codes (*ICD-9* and *ICD-10* codes)	NA	Respiratory disease (eg, COPD and asthma), cardiovascular disease, type 2 diabetes, and others
Treatment			
Surgical treatment	Procedure codes (*CPT* and *ICD-10* codes)	NA	Surgical procedure (ie, lobectomy, segmentectomy, wedge resection, video-assisted thoracic surgical procedure) with surgical admission and discharge dates
Radiation therapy	Procedure codes (*CPT* and *ICD-10* codes)	NA	Radiation therapy procedure, treatment start and end dates
Chemotherapy	Procedure codes (*CPT* and *ICD-10* codes) and medication name codes	NA	Chemotherapy procedures, chemotherapy drugs, and treatment start and end dates
Target therapy and immunotherapy	Medication name codes	NA	Target therapy and immunotherapy drugs and treatment start and end dates

Abbreviations: BMI, body mass index; COPD, chronic obstructive pulmonary disease; ECOG, Eastern Cooperative Oncology Group; EXTEND, Extraction of Electronic Medical Record Numerical Data; *ICD-9*, *International Classification of Diseases, Ninth Revision*; *ICD-10*, *International Statistical Classification of Diseases and Related Health Problems, Tenth Revision*; NA, not applicable; NICE, Natural Language Processing Interpreter for Cancer Extraction; NSCLC, non–small cell lung cancer.

### Patient Selection Criteria

This study excluded patients whose lung cancer history *ICD-9* or *ICD-10* codes were assigned before the patients were assigned lung cancer *ICD-9* or *ICD-10* codes, under the assumption that these patients had recurrent or secondary primary lung cancer. Patients with follow-up less than 14 days after diagnosis were also excluded.

### Assessment of Data Quality

#### Completeness

For each variable, we calculated the percentage of patients with at least 1 measurement for the variable. We also assessed the availability of the variables during a 2-month window before and after diagnosis. We further investigated the association between completeness and year of diagnosis, as well as the total counts of medical notes.

#### Accuracy

We evaluated accuracy, defined as the concordance of extracted cancer characteristics, including diagnosis date, histologic type, and clinical stages, with annotated variable information from 2 sources: criterion manual annotation of random samples selected from the EHR cohort and stored information from the BLCS cohort, which prospectively collected data with multiple data-collection sources.^16^ For diagnosis date, we calculated the absolute difference between the date inferred by NICE and the annotated date obtained from EHR review or BLCS and summarized the distribution of the absolute differences. The agreement between EHR-extracted data and annotation for histologic and clinical stages was summarized based on contingency tables. We additionally assessed the overall data quality by comparing hazard ratio (HR) estimates from fitting 2 Cox models for OS to the BLCS cohort with 1 model using baseline covariates extracted from EHRs and the other using covariates extracted from the BLCS database. We included baseline covariates for age, sex, race/ethnicity, smoking status, histologic type, and stage. Race/ethnicity data were collected from EHRs; race/ethnicity was assessed because it is associated with lung cancer survival. The HR and *P* values that assessed the association of each variable with lung cancer survival were compared to test the consistency of results.

### Development of a Prognostic Model for NSCLC

Patients with histologically confirmed and stage-confirmed NSCLC diagnosed from January 2000 through January 2015 in the EHR cohort were included in the analysis. We limited the age range to 18 to 90 years and excluded patients without laboratory test results within 60 days before or after diagnosis dates, given that that these patients were less likely to be treated within MGB. Routing clinically collected variables, including demographic information, smoking status, BMI, ECOG performance status, histologic type, stage, history of chronic obstructive pulmonary disease, history of asthma, history of type 2 diabetes, and common laboratory test results (ie, complete blood count and comprehensive metabolic panel), were considered candidate prognostic factors. Values of the baseline variables were determined based on their information within 60 days before or after diagnosis. For variables with multiple measurements, the measurement closest to diagnosis date was used in the analysis. Missing values were coded as a separate missing category, given that missing certain tests or variables could be informative of the patient’s health status. Variables that were categorized included BMI (ie, underweight, reference range, overweight, and obesity) and laboratory tests (ie, low, reference range, or high based on clinical range) to facilitate easier clinical interpretation.

### Statistical Analysis

The primary outcome was OS at 5 years, defined as the time from the date of diagnosis until death. Patients alive at the last follow-up or 5 years after diagnosis without evidence of death were censored. Among 11 724 patients in the study cohort, we randomly assigned 8793 patients to a training set (75.0%) and 2931 patients to a nonoverlapping testing set (25.0%) to train models and evaluate performance. Penalized Cox regression was used for training a sparse prognostic model that allowed the removal of noninformative variables. We used group minimax concave penalty as the penalty function to enable group selection of multilevel categorical covariates.^17^ Features with nonzero coefficients selected by minimax concave penalty from the training data set were used for multivariate Cox proportional hazards analyses. To facilitate use of the models in the clinical setting, the results of multivariate Cox regression model were used to build the final nomogram and generate probabilities of OS at 1 to 5 years after diagnosis.^18^ In the nomogram, the final risk score was calculated by summing the points for each item using the nomogram and aligned to the total point axis to estimate 1-year to 5-year OS probabilities.

Model discrimination accuracy was assessed based on the time-dependent receiver operating characteristic (ROC) curves and AUROC,^18,19^ as well as *C* index.^20^The AUROCs of final models were compared with the AUROC of the model for age, sex, stage, and histologic type at each year separately. Model calibration capability was assessed by agreements between predicted and observed death rates. *P* values were 2-sided, and statistical significance was set at *P* < .05. Data were analyzed using R statistical software version 3.6.1 (R Project for Statistical Computing) from March 2019 through July 2020.

## Results

### Assembly of the EHR Lung Cancer Cohort

Among 76 643 patients with at least 1 lung cancer diagnostic code, 42 069 patients were identified as having lung cancer with the classification algorithm. The study cohort consisted of 35 375 patients (16 613 men [47.0%] and 18 756 women [53.0%]; 30 140 White individuals [85.2%], 1040 Black individuals [2.9%], and 857 Asian individuals [2.4%]) after excluding 2876 patients with lung cancer history and 5302 patients with less than 14 days of follow-up after initial diagnosis (Table 2). The median (interquartile range [IQR]) age at diagnosis was 66.7 (58.4-74.1) years, and most patients had a history of smoking (32 650 patients [92.3%]). Among patients in the cohort, 27 748 patients (90.1%) had NSCLC and 3065 patients (9.9%) had small cell lung cancer (SCLC); 13 628 patients (38.5%) had received surgical treatment, 14 039 patients (39.7%) had received chemotherapy, and 14 710 patients (41.6%) had received radiation therapy within the MGB health care system with *ICD 9* and *ICD 10* codes, procedure codes, or medication codes available. In total, 2631 patients received target therapy and 503 patients received programmed death–ligand 1 or programmed cell death protein 1 inhibitors. Among 4655 patients tested using the SNaPshot assay,^21^ 2183 patients (46.9%) were positive for at least 1 variant in at least 1 of 3 genes, namely *KRAS* (1242 patients [26.7%]), *EGFR* (857 patients [18.4%]), and *BRAF* (171 patients [3.7%]). Translocation was tested for *ALK* among 3791 patients, with positive results among 203 patients (5.4%), and *ROS1* among 2436 patients, with positive results among 51 patients (2.1%). The median (IQR) follow-up time was 1.62 (0.63-4.14) years, and the estimated median OS was 2.51 years (95% CI, 2.45 years-2.57 years).

**Table 2.** Demographic and Clinical Characteristics of Final Cohort

Characteristic	No. (%) (N = 35 375)
Age at initial diagnosis, median (IQR), y	66.7 (58.4-74.1)
Sex	
Women	18 756 (53.0)
Men	16 613 (47.0)
Unknown	6 (0.02)
Race/ethnicity	
White	30 140 (85.2)
Black	1040 (2.9)
Asian	857 (2.4)
Hispanic	323 (0.9)
Other	267 (0.8)
Unknown	2748 (7.8)
Smoking status	
Smoker	32 650 (92.3)
Nonsmoker	2725 (7.7)
Histologic type	
Completeness	30 813 (87.1)
Adenocarcinoma	18 331 (59.5)
Squamous cell carcinoma	5816 (18.9)
NSCLC unspecified	3601 (11.7)
Small cell lung cancer	3065 (9.9)
Stage	
Completeness	26 843 (75.9)
1	7083 (26.4)
2	3069 (11.4)
3	5889 (21.9)
4	8495 (31.6)
Limited	1222 (4.6)
Extensive	1085 (4.0)
Treatment received within MGB health care system^a^	
Surgical treatment	13 628 (38.5)
Chemotherapy	14 039 (39.7)
Radiation therapy	14 710 (41.6)
Target therapy	2631 (7.4)
Immunotherapy	504 (1.4)
*EGFR* tested^b^	4655 (13.1)
*EGFR* status	
Variant positive	857 (18.4)
Variant negative	3798 (81.6)
*KRAS* tested^b^	4655 (13.1)
*KRAS* status	
Variant positive	1242 (26.7)
Variant negative	3413 (73.3)
*BRAF* tested^b^	4655 (13.1)
*KRAF* status	
Variant positive	171 (3.7)
Variant negative	4484 (96.3)
*ALK* tested^c^	3791 (10.1)
*ALK* status	
Rearrangement present	203 (5.4)
Rearrangement not present	3588 (81.6)
*ROS* tested^c^	2436 (6.9)
*ROS* status	
Rearrangement present	51 (2.1)
Rearrangement not present	2385 (97.9)
Follow-up from initial diagnosis, median (IQR), y	1.62 (0.63-4.14)

Abbreviations: IQR, interquartile range; MGB, Mass General Brigham; NSCLC, non–small cell lung cancer.

^a^ Patients received treatments within the MGB health care system with *International Classification of Diseases, Ninth Revision* (*ICD-9*) or *International Statistical Classification of Diseases and Related Health Problems, Tenth Revision* (*ICD-10*) codes, procedure codes, or medication codes available.

^b^ *EGFR*, *KRAS*, and *BRAF* were tested using the SNaPshot assay (Thermo Fisher Scientific).

^c^ *ALK* and *ROS* were tested using fluorescence in situ hybridization or immunohistochemistry.

The lung cancer classification model attained an AUROC of 0.927. By setting a threshold value to achieve specificity of 90.0%, we achieved a sensitivity of 75.2%, a PPV of 94.4%, and an *F* score of 0.837.

### Data Completeness

In structured data, date of birth, sex, and race/ethnicity were available for 35 375 patients (100%), 35 369 patients (99.97%), and 32 627 patients (92.2%), respectively; 23 949 patients (59.9%) had at least 1 lung cancer–related therapy within MGB with specific treatment procedure codes and medication codes. For common laboratory tests, 29 184 patients (82.5%) had at least 1 measurement and 23 949 patients (67.7%) had at least 1 measurement within 60 days before or after the diagnosis date. Variables that needed to be extracted from clinical notes frequently had a greater number of missing values. For cancer characteristics, there were 30 813 patients (87.1%) with histologic type, 26 843 patients (75.9%) with stage, and 10 754 patients (30.4%) with ECOG performance status data. For BMI, 17 546 patients (49.6%) had at least 1 measurement and 13 761 patients (38.9%) were measured at baseline (eTable 3 in the Supplement). In general, we found that the completeness was higher for patients with more recent diagnoses. For example, BMI completeness increased from 56 of 497 patients (11.2%) in 1994 to 1399 of 2282 patients (61.3%) in 2016. The increase followed the gradual process of EHR adoption within the MGB health care system. The completeness was also higher for patients with greater health care use. For example, BMI completeness increased from 148 of 1767 patients (8.4%) among those with fewer than 10 health care visit days recorded lifetime to 12 621 of 19 417 patients (65.0%) among those with more than 100 health care visit days recorded lifetime (eFigure 2 in the Supplement).

### Data Accuracy

Compared with 6225 patients in BLCS, the extracted diagnosis date combining *ICD-9* and *ICD-10* and NICE had a median (IQR) discrepancy of 0 (−12 to 9) days, and there were absolute discrepancies of less than 90 days, less than 180 days, and less than 1 year among 5431 patients (87.6%), 5677 patients (91.2%), and 5827 patients (93.6%), respectively. Compared with 67 patients with EHR review, 60 patients (89.6%), 61 patients (91.0%), and 62 patients (92.5%) had absolute discrepancies of less than 90 days, less than 180 days, and less than 1 year, respectively (eTable 4 and eFigure 3 in the Supplement). The accuracy of the diagnosis dates was higher than that using *ICD-9* or *ICD-10* time only compared with the EHR review result (eTable 4 in the Supplement). Histologic type showed great accuracy and agreement, with 3 discrepancies compared with 67 EHR reviews (4.5%) and 514 discrepancies compared with 5526 BLCS patients (9.3%) (eTable 5 in the Supplement). Among 514 histologic type discrepancies, 185 discrepancies (36.0%) arose from discriminating between adenocarcinoma and non–small cell unspecified cancer. For stages, there were 11 discrepancies compared with 63 EHR reviews (19.3%) and 957 discrepancies compared with 5189 BLCS patients (18.4%); among 957 stage discrepancies, 413 discrepancies (43.2%) were 1 stage category off (eTable 6 in the Supplement). For patients with NSCLC or SCLC, Cox proportional models yielded similar estimates of the HR for age, sex, histologic type, and stage (eTable 7 and eTable 8 in the Supplement).

### Prognostic Model for NSCLC

Among 16 648 patients with NSCLC identified from January 2000 through January 2015, 61 patients younger than age 18 years or older than age 90 years were excluded. Additionally, 4854 patients without routine blood test results within 60 days before or after diagnosis dates were excluded. A total of 11 724 patients were included in the final analysis, with 8793 patients in the training set and 2931 patients in the testing set. Baseline characteristics of the patients and distributions of laboratory variables are summarized in eTable 9 and eTable 10 in the Supplement. Among collected variables, ECOG performance status had missing values higher than 30% (missing for 9682 patients at diagnosis [82.5%])and was excluded from further analysis.

**Figure 2.**
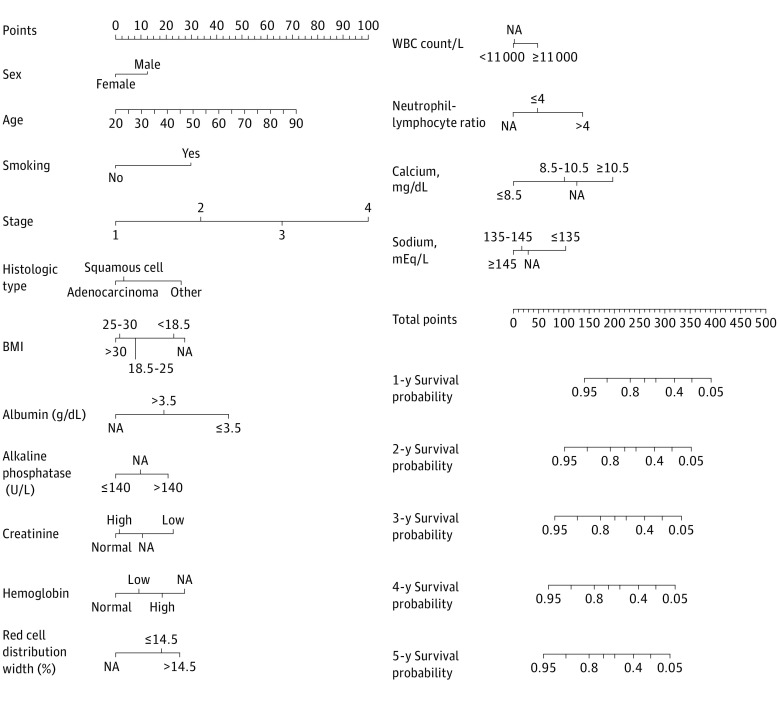
Prognostic Nomogram for Patients With Non–Small Cell Lung Cancer The results of multivariate Cox regression model incorporating variables from panelized regression were used to build the final nomogram and generate probabilities of overall survival at 1 year to 5 years after diagnosis. BMI indicates body mass index; NA, not applicable; WBC, white blood cell. SI conversion factors: To convert albumin to grams per liter, multiply by 10; alkaline phosphatase to microkatals per liter, multiply by 0.0167; calcium to millimoles per liter, multiply by 0.25; creatinine to micromoles per liter, multiply by 88.4; hemoglobin to grams per liter, multiply by 10.0; sodium to millimoles per liter, multiply by 1.0; WBC count to × 10^9^ per liter, multiply by 0.001.

The group minimax concave penalty retained 15 variables with nonzero coefficients in the training set; these included age, sex, smoking status, histologic type, stage, BMI, albumin levels, alkaline phosphatase levels, creatinine levels, hemoglobin levels, red cell distribution width, white blood count, neutrophil-lymphocyte ratio, calcium levels, and sodium levels. These variables were used for multivariate Cox proportional hazards analysis and construction of the nomogram. The variables were independent predictors associated with OS (eg, in the testing set, men: HR, 1.303 [95% CI, 1.17-1.44]; *P* < .001; stage 4 cancer vs stage 1 cancer: HR, 4.83 [95% CI, 4.16-5.62]; *P* < .001; squamous cell carcinoma vs adenocarcinoma: HR, 1.14 [95% CI, 1.01-1.29]; *P* = .03; neutrophil-lymphocyte ratio: HR, 1.23 [95% CI, 1.10-1.38]; *P* < .001) (Table 3). A nomogram is shown in Figure 2.

**Table 3.** Factors Associated With Overall Survival at 5 Years

Factor	Training set		Testing set	
	HR (95% CI)	*P* value	HR (95% CI)	*P* value
Sex				
Women	1 [Reference]	NA	1 [Reference]	NA
Men	1.23 (1.16-1.30)	<.001	1.303 (1.17-1.44)	<.001
Age	1.02 (1.01-1.02)	<.001	1.02 (1.01-1.02)	<.001
Smoking status				
Nonsmoker	1 [Reference]	NA	1 [Reference]	NA
Smoker	1.66 (1.46-1.89)	<.001	1.92 (1.51-2.44)	<.001
Stage				
1	1 [Reference]	NA	1 [Reference]	NA
2	1.73 (1.54-1.94)	<.001	1.32 (1.08-1.61)	<.001
3	2.92 (2.66-3.20)	<.001	2.44 (2.08-2.86)	<.001
4	5.08 (4.65-5.54)	<.001	4.83 (4.16-5.62)	<.001
Histologic type				
Adenocarcinoma	1 [Reference]	NA	1 [Reference]	NA
Squamous cell carcinoma	1.05 (0.98-1.13)	.16	1.14 (1.01-1.29)	.03
Other	1.52 (1.40-1.65)	<.001	1.32 (1.16-1.51))	<.001
BMI				
Reference range	1 [Reference]	NA	1 [Reference]	NA
Obesity	0.88 (0.80-0.97)	.01	0.86 (0.73-1.01)	.06
Overweight	0.90 (0.83-0.98)	.02	0.90 (0.78-1.04)	.15
Underweight	1.28 (1.06-1.55)	.01	0.94 (0.65-1.35)	.73
Missing	1.38 (1.28-1.49)	<.001	1.32 (1.16-1.51)	<.001
Albumin, g/dL				
≤3.5	1 [Reference]	NA	1 [Reference]	NA
>3.5	0.66 (0.61-0.71)	<.001	0.59 (0.52-0.68)	<.001
Missing	0.48 (0.41-0.57)	<.001	0.45 (0.34-0.61)	<.001
Alkaline phosphatase, U/L				
≤140	1 [Reference]	NA	1 [Reference]	NA
>140	1.40 (1.27-1.54)	<.001	1.54 (1.29-1.83)	<.001
Missing	1.17 (0.99-1.38)	.06	1.09 (0.81-1.47)	.57
Creatinine, mg/dL				
Reference range^a^	1 [Reference]	NA	1 [Reference]	NA
High	1.02 (0.95-1.10)	.57	0.93 (0.81-1.06)	.27
Low	1.45 (1.26-1.67)	<.001	1.45 (1.26-1.67)	<.001
Missing	1.19 (0.90-1.57)	.22	0.93 (0.81-1.06)	.16
Hemoglobin, g/dL				
Reference range^b^	1 [Reference]	NA	1 [Reference]	NA
High	1.35 (1.08-1.70)	.01	1.34 (0.89-2.03)	.15
Low	1.16 (1.09-1.24)	<.001	1.06 (0.95-1.19)	.28
Missing	1.56 (0.96-2.55)	.07	0.54 (0.18-1.65)	.32
Red cell distribution width, %				
≤14.5	1 [Reference]	NA	1 [Reference]	NA
>14.5	1.12 (1.05-1.20)	<.001	1.36 (1.20-1.53)	<.001
Missing	0.74 (0.46-1.20)	.23	0.84 (0.27-2.61)	.77
WBC count, per μL				
4500-11 000	1 [Reference]	NA	1 [Reference]	NA
≥11 000	1.17 (1.09-1.25)	<.001	1.24 (1.10-1.40)	<.001
Missing	1.01 (0.64-1.59)	.97	2.82 (0.73-10.91)	.13
Neutrophil-lymphocyte ratio				
≤4	1 [Reference]	NA	1 [Reference]	NA
>4	1.34 (1.25-1.43)	<.001	1.23 (1.10-1.38)	<.001
Missing	0.85 (0.76-0.96)	.01	0.98 (0.80-1.20)	.83
Calcium, mg/dL				
8.5-10.5	1 [Reference]	NA	1 [Reference]	NA
≥8.5	0.72 (0.66-0.79)	<.001	0.68 (0.58-0.79)	<.001
≥10.5	1.37 (1.17-1.59)	<.001	1.56 (1.15-2.11)	.003
Missing	1.08 (0.84-1.40)	.54	1.12 (0.75-1.67)	.59
Sodium, mEq/L				
135-145	1 [Reference]	NA	1 [Reference]	NA
<135	1.32 (1.23-1.43)	<.001	1.25 (1.10-1.43)	<.001
>145	0.95 (0.77-1.16)	.59	1.07 (0.74-1.55)	.72
Missing	1.04 (0.73-1.48)	.82	0.97 (0.58-1.65)	.92

Abbreviations: BMI, body mass index; HR, hazard ratio; NA, not applicable; WBC, white blood cell.

SI conversion factors: To convert albumin to grams per liter, multiply by 10; alkaline phosphatase to microkatals per liter, multiply by 0.0167; calcium to millimoles per liter, multiply by 0.25; creatinine to micromoles per liter, multiply by 88.4; hemoglobin to grams per liter, multiply by 10.0; sodium to millimoles per liter, multiply by 1.0; WBC count to × 10^9^ per liter, multiply by 0.001.

^a^ The reference range for creatinine is 0.6 to 1.2 mg/dL for men and 0.5 to 1.1 mg/dL for women.

^b^ The reference range for hemoglobin is 13.5 to 17.5 g/dL for men and 12.0 to 15.5 g/dL for women.

The AUROCs were 0.828 (95% CI, 0.815-0.842) for 1-year prediction, 0.825 (95% CI, 0.812-0.836) for 2-year prediction, 0.814 for 3-year prediction (95% CI, 0.800-0.826), 0.814 (95% CI, 0.799-0.828) for 4-year prediction, and 0.812 (95% CI, 0.798-0.825) for 5-year prediction in the testing set (eFigure 4 in the Supplement). The prognostic ability of the proposed final model was statistically significantly better compared with the AUROCs of the base model, which included sex, age, histologic type, and stage (1-year prediction: 0.774 [95% CI, 0.758-0.789]; *P* < .001; 2-year prediction: 0.779 [95% CI, 0.765-0.793]; *P* = .002; 3-year prediction: 0.780 [95% CI, 0.766-0.796]; *P* = .002; 4-year prediction: 0.782 [95% CI, 0.767-0.797]; *P* = .001; 5-year prediction: 0.782 [95% CI, 0.768-0.798]; *P* < .001). In addition, the *C* indexes were 0.726 and 0.697 for final and basic model separately in the testing set, respectively. In calibration plots, observed probabilities of OS were generally within 95% CI of the predicted probabilities of OS (eFigure 5 in the Supplement).

## Discussion

In this cohort study, we applied a classification algorithm to identify a cohort of patients with lung cancer with high PPV and sensitivity. We found that NICE was able to reliably extract important cancer prognostic factor information embedded in EHR notes, including cancer stage, histologic type, and somatic variants. The tool extracted information from all notes rather than focusing on pathology notes only, which was associated with improved sensitivity in the extractions. In this quality assessment, we found that HR estimates obtained from BLCS cohort data were similar to those obtained from extracted EHR data. The magnitude of data completeness increased over the diagnosis year and was associated with health care use. Missing data in the EHR are often informative, and hence the availability of some variables themselves could be predictive factors associated with clinical states.^22^ Our study used a variety of strategies to improve EHR data quality, including using machine learning algorithms to classify cancer status and using NICE to extract factors associated with cancer prognosis from clinical notes. These strategies are generally applicable to the assembly of other EHR cancer cohorts.

In addition, this large-scale, EHR-based cohort study collected detailed longitudinal measurements of clinical factors and patient care data over time. Our cohort offers different data elements not typically found in registries from SEER, such as real-world treatment and follow-up data, genetic and molecular profiling, and clinical laboratory test results. This cohort may augment various studies on prognosis. For example, this cohort may help provide a better understanding of the association between specific drugs and improved survival outcomes, modeling clinical outcomes with comprehensive variables collected in routine clinical care.

### Limitations

This study has several limitations. First, patient mortality data were incomplete, given that patients may leave the health care system and data may not be updated frequently when death occurs outside of the health care system. One possible solution is to augment incomplete mortality data with other sources. Second, the determination of diagnosis dates for patients who had recurrences or transferred from other hospitals is challenging. Although we excluded patients with lung cancer history codes that were recorded before their lung cancer–related codes, the current data were inadequate to identify patients who were initially diagnosed elsewhere. Third, extracting stage through NLP is challenging, given that there are many discrepancies and uncertainties in the notes. Fourth, structured data may not be enough to capture the complete information of treatment, given that patients may receive treatments outside of the health care system. Studies aiming to evaluate the association of treatment with cancer progression may need to use codified and NLP data to ascertain treatment information. Fifth, patients within MGH and BWH were not a random sample from the US population. This may affect the generalizability to other medical institutions of the models trained on our data. Thus, external validation of our model in other populations is desirable.

## Conclusions

We assembled a large lung cancer cohort from EHRs using a phenotyping algorithm and extraction strategies combining structured and unstructured data. Our findings suggest that a prognostic model based on EHR cohort may be used conveniently to facilitate prediction of NSCLC survival.

## Notes

Supplement. **eAppendix.** Supplemental Methods **eTable 1.** Coefficients of Smoking Algorithm **eTable 2.** Dictionaries for Natural Language Processing Tools **eTable 3.** Completeness of Variables in Final Cohort **eTable 4.** Discrepancies Between Electronic Health Record Diagnosis Date and Random Samples and Boston Lung Cancer Study Diagnosis Date **eTable 5.** Histologic Type From Boston Lung Cancer Study Cohort and Random Samples vs From Electronic Health Records **eTable 6.** Stage From Boston Lung Cancer Study Cohort and Random Samples vs From Electronic Health Records **eTable 7.** Multivariate Cox Proportional Hazards Regression for Patients With Non–Small Cell Lung Cancer in Boston Lung Cancer Study and Electronic Health Record Data **eTable 8.** Multivariate Cox Proportional Hazards Regression for Patients With Small Cell Lung Cancer in Boston Lung Cancer Study and Electronic Health Record Data **eTable 9.** Basic Characteristics of Patients in Non–Small Cell Lung Cancer Prognostic Model **eTable 10.** Values of Laboratory Variables for Patients in Non–Small Cell Lung Cancer Prognostic Model **eFigure 1.** Flow Chart of Natural Language Processing Interpreter for Cancer Extraction (NICE) **eFigure 2.** Completeness of Type, Stage, Body Mass Index, and Eastern Cooperative Oncology Group Performance Status Improvement Over Time With Total Counts of Visit Days **eFigure 3.** Histogram of Date Discrepancies for Extracted Diagnosis Date From Electronic Health Records Compared With Record Review and Boston Lung Cancer Study Cohort Diagnosis Date **eFigure 4.** Time-Dependent Area Under the Receiver Operating Characteristic Curves and 95% CIs for 1 to 5 Years in Testing Set **eFigure 5.** Calibration Curves Comparing Predicted and Actual Survival Probabilities at 1, 3, and 5 Years for Training and Testing Sets
