# Supplementary material for: Performance of a Machine Learning Algorithm Using Electronic Health Record Data to Identify and Estimate Survival in a Longitudinal Cohort of Patients With Lung Cancer
Source: JAMA Netw Open. 2021 Jul 7;4(7):e2114723. doi: 10.1001/jamanetworkopen.2021.14723 (PMC8264641; doi:10.1001/jamanetworkopen.2021.14723)
Supplement: Supplement. — eAppendix. Supplemental Methods eTable 1. Coefficients of Smoking Algorithm eTable 2. Dictionaries for Natural Language Processing Tools eTable 3. Completeness of Variables in Final Cohort eTable 4. Discrepancies Between Electronic Health Record Diagnosis Date and Random Samples and Boston Lung Cancer Study Diagnosis Date eTable 5. Histologic Type From Boston Lung Cancer Study Cohort and Random Samples vs From Electronic Health Records eTable 6. Stage From Boston Lung Cancer Study Cohort and Random Samples vs From Electronic Health Records eTable 7. Multivariate Cox Proportional Hazards Regression for Patients With Non–Small Cell Lung Cancer in Boston Lung Cancer Study and Electronic Health Record Data eTable 8. Multivariate Cox Proportional Hazards Regression for Patients With Small Cell Lung Cancer in Boston Lung Cancer Study and Electronic Health Record Data eTable 9. Basic Characteristics of Patients in Non–Small Cell Lung Cancer Prognostic Model eTable 10. Values of Laboratory Variables for Patients in Non–Small Cell Lung Cancer Prognostic Model eFigure 1. Flow Chart of Natural Language Processing Interpreter for Cancer Extraction (NICE) eFigure 2. Completeness of Type, Stage, Body Mass Index, and Eastern Cooperative Oncology Group Performance Status Improvement Over Time With Total Counts of Visit Days eFigure 3. Histogram of Date Discrepancies for Extracted Diagnosis Date From Electronic Health Records Compared With Record Review and Boston Lung Cancer Study Cohort Diagnosis Date eFigure 4. Time-Dependent Area Under the Receiver Operating Characteristic Curves and 95% CIs for 1 to 5 Years in Testing Set eFigure 5. Calibration Curves Comparing Predicted and Actual Survival Probabilities at 1, 3, and 5 Years for Training and Testing Sets [file jamanetwopen-e2114723-s001.pdf]

Supplemental Online Content

Yuan Q, Cai T, Hong C, et al. Performance of a machine learning algorithm using electronic health record data to identify and estimate survival in a longitudinal cohort of patients with lung cancer. *JAMA Netw Open*. 2021;4(7):e2114723. doi:10.1001/jamanetworkopen.2021.14723

eAppendix. Supplemental Methods
eTable 1. Coefficients of Smoking Algorithm
eTable 2. Dictionaries for Natural Language Processing Tools
eTable 3. Completeness of Variables in Final Cohort
eTable 4. Discrepancies Between Electronic Health Record Diagnosis Date and Random Samples and Boston Lung Cancer Study Diagnosis Date
eTable 5. Histologic Type From Boston Lung Cancer Study Cohort and Random Samples vs From Electronic Health Records
eTable 6. Stage From Boston Lung Cancer Study Cohort and Random Samples vs From Electronic Health Records
eTable 7. Multivariate Cox Proportional Hazards Regression for Patients With Non–Small Cell Lung Cancer in Boston Lung Cancer Study and Electronic Health Record Data
eTable 8. Multivariate Cox Proportional Hazards Regression for Patients With Small Cell Lung Cancer in Boston Lung Cancer Study and Electronic Health Record Data
eTable 9. Basic Characteristics of Patients in Non–Small Cell Lung Cancer Prognostic Model
eTable 10. Values of Laboratory Variables for Patients in Non–Small Cell Lung Cancer Prognostic Model
eFigure 1. Flow Chart of Natural Language Processing Interpreter for Cancer Extraction (NICE)
eFigure 2. Completeness of Type, Stage, Body Mass Index, and Eastern Cooperative Oncology Group Performance Status Improvement Over Time With Total Counts of Visit Days
eFigure 3. Histogram of Date Discrepancies for Extracted Diagnosis Date From Electronic Health Records Compared With Record Review and Boston Lung Cancer Study Cohort Diagnosis Date
eFigure 4. Time-Dependent Area Under the Receiver Operating Characteristic Curves and 95% CIs for 1 to 5 Years in Testing Set
eFigure 5. Calibration Curves Comparing Predicted and Actual Survival Probabilities at 1, 3, and 5 Years for Training and Testing Sets

This supplemental material has been provided by the authors to give readers additional information about their work.

© 2021 Yuan Q et al. *JAMA Network Open.*

eAppendix. Supplemental Methods

Data extraction details

Structured data extraction

Initial population were drawn from MGB healthcare with at least one lung cancer related ICD codes (162, 1620, 1622, 1623, 1624, 1625, 1628, 1629, 20921, 2312, ICD10:C33, ICD10:C34.00, ICD10:C34.10, ICD10:C34.2, ICD10:C34.30, ICD10:C34.80, ICD10:C34.90, ICD10:C7A.090, ICD10:D02.20, ICD10:Z85.118, V1011).

Birthday, sex, and race/ethnicity were stored in the structured coded data. Birthday was used to calculate the age of diagnosis. Race was categorized into five categories: White, Asian, Black, Hispanic, and others. Common treatments for lung cancer patients including surgery, chemotherapy, radiation therapy, target therapy, and immunotherapy could be found in the prescribed medications and billing codes. Surgery and radiation therapy were extracted using ICD-9/10 and Common Procedural Terminology (CPT) codes. Chemotherapy, target therapy, and immunotherapy were extracted from ICD-9/10-CM, CPT, and medication codes. Common laboratory tests included complete blood count, metabolic panel, lipid panel, liver panel, hemoglobin A1C and urinalysis. The numeric value with its measurement dates were extracted using structured codes.

Unstructured data extraction using NLP tools

We used the published tool EXTEND to extract ECOG and BMI information. For other variables including cancer stage, histology, and mutation information, we developed a new tool named NICE to perform data extraction from clinical notes including discharge summary, progress notes (Figure1). All dictionaries were shared (separate file). The flow chat of NICE was shown in eFigure 1.

Lung cancer concepts

For the extraction of lung cancer concepts, we built a dictionary containing all synonyms of the concept 'lung cancer' such as 'lung cancer' and 'lung carcinoma' using the Unified Medical Language System. All notes were processed to identify the positive mention of the 'lung cancer' concept via Named Entity Recognition (e.g., 'lung cancer' in a sentence like 'The patient denies lung cancer history' was ignored). Date information was also extracted if a date was mentioned in the same sentence that the concept of 'lung cancer' was located (e.g., "Lung cancer (HCC) 11/18/2014"). The date "11/18/2014" was assigned to the concept mention of 'lung cancer'. The most mentioned dates of the 'lung cancer' concept was combined with ICD-9/10 codes time to choose the earlier time as the cancer diagnosis date.

Stage and histology type

To extract stage and histology type, we built dictionaries for both that are similar as those for the lung cancer concept. Then we processed notes to identify positive mention of stage and histology. Because stage information can also be mentioned for other diseases such as other cancers, sleep status, bed sore, and chronic kidney disease, we ignored the mention of stage with mention of any of these diseases in the same sentence. We also excluded the mention of histology if there was mention of a cancer other than lung cancer. The mention of stage and histology were categorized into three confidence levels: high, medium, and low. A high confidence level was assigned when the lung cancer concept appeared in the same sentence. A medium confidence level was assigned when the lung cancer concept appeared in the same note instead of the same sentence. We assigned a low confidence level to stage or histology concept if there was no mention of lung cancer concept in the same note. We built regular expression patterns for extracting TNM stage as additional stage information, and then converted TNM stage to clinical stage. For histological type, phrases were grouped into four categories: NSCLC, SCLC, adenocarcinoma, and squamous cell carcinoma. If at least two histological types were mentioned, the most commonly occurring phrases were selected. If the most commonly occurring type was NSCLC, we chose the most occurring subtype: adenocarcinoma or squamous cell carcinoma. If none of these subtypes were mentioned, the histological type was defined as non-small cell unspecified. For stage, phrases were extracted and grouped into the seven categories: stage I, stage II, stage III, and stage IV, extensive stage, limited stage, and metastatic.

© 2021 Yuan Q et al. *JAMA Network Open.*

Somatic mutations
Gene alterations that were listed in the NCCN guideline and have been identified that impact therapy selection including *EGFR, KRAS, ALK, ROS1,* and *BRAF*. For patients who received tumor diagnostic tests from MGB HealthCare System including the Snapshot assay, fluorescence in situ hybridization, and immunohistochemistry, we extracted results from the molecular pathology reports. The process of extraction was similar to that used for stage and histology, but we did not categorize the confidence level because the mention of these genetic variables in pathology notes was specific without ambiguation.

Smoking status
For smoking status, each patient was assigned as smoker or non-smoker, and was predicted using the classification model combining structured coded data and clinical notes. To develop the smoking algorithm, we adapted a standardized phenotyping process using NLP and machine learning (ref). We built the smoking algorithm for the entire data mart with 74,421 patients.

We considered six codified features: 1) diagnosis code for ever smoking; 2) EMR current smoking; 3) medical use of Bupropion; 4) nicotine replacement therapy; 5) medical use of varenicline; and 6) tobacco cessation counseling. We considered three most important smoking concepts: C0037369 (smoking), C0337664 (smoker) and C1519384 (tobacco smoking history). We processed free-text clinical notes using NLP to count the number of positive mentions of each smoking concept for each patient. In addition, we measured healthcare utilization as the total number of medical notes in each patient's EHR. To select the most informative features, we first constructed three surrogate labels using diagnosis code for ever smoking, main smoking CUI C0037369, and the combination of two former surrogates. We then repeatedly fitted lasso penalized logistic regression to each of three surrogate labels against all candidate features. Features that were selected >50% of the time were retained for subsequent supervised algorithm training.

Among the 74,421 patients, 4,922 were also from the BLCS, thus having the gold standard training labels for current smoker, past smoker, and non-smoker. Then the smoking labels were further categorized into either: smoker (including both current smoker and past smoker) or non-smoker.

We developed a smoking algorithm using lasso penalized logistic regression, which further reduced the number of variables in the model. Comparing against the chart review gold-standard, the performance characteristics of each algorithm was reported using the AUC, PPV, sensitivity, and specificity. Cross-validation with 70:30 splits averaged over 100 random partitions was used to correct for over-fitting bias. The algorithms assigned each patient their probability of being a smoker. Those with probabilities above a threshold that achieved 90% PPV were classified as smokers.

Of the 4,922 patients with gold standard labels, 4,314 (87.6%) were identified as smokers and 608 were identified as non-smokers. Six smoking features were selected as the most informative features, as shown in eTable 1. The six selected features were then fitted into the lasso model. The estimated regression coefficients are summarized in eTable 1. When compared with gold standard labels, the smoking algorithm achieved an AUC of 0.852 after cross-validation. With the probability threshold set to provide 90% PPV, the smoking algorithm identified 69,021 patients as smokers with 97.6% sensitivity.

BMI and ECOG
To calculate BMI, height and weight or calculated BMI recorded with measurement date were extracted from the structured data and clinical notes using the NLP tool EXTEND. ECOG performance status with the documented dates was extracted using EXTEND.

© 2021 Yuan Q et al. *JAMA Network Open.*

Prognostic model pre-selected variables

Demographic (age, sex, race), smoking status (smoker, nonsmoker), BMI, ECOG performance status, tumor characteristics (histological type, stage), history of COPD, history of asthma, history of type 2 diabetes, and common laboratory tests were collected. Laboratory tests were from complete blood count (CBC) and comprehensive metabolic panel (CMP). CBC includes WBC, neutrophil, lymphocyte, monocyte, and eosinophil and their ratio such as NLR, red blood count (RBC), RDW, HGB, hematocrit, platelet count (PLT), and mean corpuscular volume (MCV). Routine CMP panel includes albumin, total bilirubin, ALP, alanine aminotransferase (ALT) and aspartate aminotransferase (AST), blood urea nitrogen (BUN), creatinine, glucose, calcium, sodium, potassium, and chloride.

Sensitivity analysis for prognostic model

In main analysis, we coded missing as a separate group given that missing could itself to informative for general health status. In this sensitivity analysis, we used multiple imputations for the missing values and evaluated the final model performance. Time-dependent AUCs are 0.824, 0.824 0.812, 0.810 and 0.808 for 1- to 5-year OS prediction, which are similar to our missing indictor methods.

© 2021 Yuan Q et al. *JAMA Network Open.*

eTable 1. Coefficients of Smoking Algorithm

Variable	Coefficients
Intercept	3.103
Diagnosis code for ever smoking	4.777
Medical use of nicotine replacement therapy	4.148
C0337664Y	0.000
C1519384Y	1.155
C0037369Y	0.328
Healthcare utilization	-0.565

Final variables retained in the smoking algorithm using lasso penalized logistic regression.

© 2021 Yuan Q et al. *JAMA Network Open.*

eTable 2. Dictionaries for Natural Language Processing Tools

1. CUIs for lung cancer

Term	CUI
acinar cell carcinoma of lung	C2146663
acinar cell carcinoma of lung	C3873379
acinar cell cystadenocarcinoma of lung	C2146675
adenocarcinoma cancer lungs	C0152013
adenocarcinoma cancer lung	C0152013
adenocarcinoma lung cancer	C0152013
adenocarcinoma lungs	C0152013
adenocarcinoma lung	C0152013
adenocarcinoma of lung	C0152013
adenocarcinoma of the lung	C0152013
adenocarcinomas lung	C0152013
adenocystic carcinoma of lung	C1334439
adenocystic carcinoma of the lung	C1334439
adenoid cystic carcinoma of lung	C1334439
adenoid cystic carcinoma of the lung	C1334439
adenoid squamous cell carcinoma of lung	C2205881
adenosquamous carcinoma of lung	C0279557
adenosquamous cell lung cancer	C0279557
adenosquamous cell lung carcinoma	C0279557
adenosquamous lung carcinoma	C0279557
adult type pleomorphic rhabdomyosarcoma of lung	C2205917
alveolar adenocarcinoma	C0007120
alveolar carcinoma	C0007120
alveolar cell carcinoma	C0007120
anaplastic carcinoma of lung	C0345958
anaplastic large cell lung cancer	C0345958
anaplastic lung carcinoma	C0345958
angiomyosarcoma of lung	C2205901
atypical carcinoid tumor of lung	C1708766
bac	C0007120
basaloid carcinoma of lung	C2205887
basaloid squamous cell carcinoma of lung	C3873339
biopsy of lung showed malignant neoplasm	C4030747
blastoma of lung	C0206629
blastoma of the lung	C0206629
bronchial cancer	C0007121
bronchial carcinoma	C0007121
bronchioalveolar adenocarcinoma of lung	C0007120

© 2021 Yuan Q et al. *JAMA Network Open.*

bronchioalveolar adenocarcinoma of the lung	C000 7120
bronchioalveolar carcinoma	C000 7120
bronchioalveolar lung carcinoma	C000 7120
bronchiogenic carcinoma	C000 7121
bronchiolar adenocarcinoma	C000 7120
bronchiolar alveolar cell carcinoma	C000 7120
bronchiolar carcinoma	C000 7120
bronchioloalveolar adenocarcinoma of lung	C000 7120
bronchioloalveolar adenocarcinoma of the lung	C000 7120
bronchioloalveolar adenocarcinoma	C000 7120
bronchioloalveolar carcinoma	C000 7120
bronchioloalveolar lung adenocarcinoma	C000 7120
bronchoalveolar cancer	C000 7120
bronchoalveolar carcinoma	C000 7120
bronchoalveolar cell lung cancer	C000 7120
bronchogenic carcinoma squamous cell type	C131 4696
bronchogenic carcinoma	C000 7121
bronchogenic lung carcinoma	C000 7121
bronchus cancer	C000 7121
bronchus carcinoma cell squamous	C131 4696

bronchus carcinoma squamous cell	C131 4696
bronchus carcinoma	C000 7121
cancer bronchus	C000 7121
cancer cell lung non small	C000 7131
cancer cell lung oat	C014 9925
cancer cell lung small	C014 9925
cancer cell lung squamous	C014 9782
cancer cell lungs squamous	C014 9782
cancer cells large lung	C034 5958
cancer cells lung non small	C000 7131
cancer cells lung squamous	C014 9782
cancer lung metastatic	C015 3676
cancer lung secondary	C015 3676
cancer lungs metastatic	C015 3676
cancer metastatic to lung	C015 3676
cancer of bronchus	C000 7121
cancer of left lung	C386 2552
cancer of lung	C024 2379
cancer of lung	C068 4249
cancer of parietal pleura	C200 4481

© 2021 Yuan Q et al. *JAMA Network Open.*

cancer of right lung	C386 2551
cancer of the lung	C024 2379
cancer of the lung	C068 4249
cancer of visceral pleura	C260 7950
cancer pulmonary	C024 2379
cancer with pulmonary metastases	C015 3676
cancers cell lung squamous	C014 9782
cancers lungs	C024 2379
carcinoid lung tumors	C028 0089
carcinoid lung tumor	C028 0089
carcinoid lung tumour	C028 0089
carcinoid pulmonary tumors	C028 0089
carcinoid tumor lung	C028 0089
carcinoid tumor of lung	C028 0089
carcinoid tumor of the lung	C028 0089
carcinoid tumour of lung	C028 0089
carcinoid tumour pulmonary	C028 0089
carcinoma cell large lungs	C034 5958
carcinoma cell large lung	C034 5958
carcinoma cell lung non small	C000 7131

carcinoma cell lung squamous	C014 9782
carcinoma cell lungs squamous	C014 9782
carcinoma lungs	C068 4249
carcinoma lung	C068 4249
carcinoma of bronchus	C000 7121
carcinoma of lung paranchyema	C034 5957
carcinoma of lung parenchyma	C034 5957
carcinoma of lung	C068 4249
carcinoma of lung	C316 3822
carcinoma of the bronchus	C000 7121
carcinoma of the lung	C068 4249
carcinoma simplex of lung	C220 0128
carcinomas lung	C068 4249
carcinosarcoma of lung	C171 1276
carcinosarcoma of lung	C387 3261
chemodectoma of lung	C204 5776
clear cell carcinoma of lung	C034 5959
clear cell squamous cell carcinoma of lung	C387 3340
clear cell type neoplasm of lung	C207 5625
cloacogenic carcinoma of lung	C207 5838

© 2021 Yuan Q et al. *JAMA Network Open.*

combined carcinoma of lung	C1333123
combined carcinoma of the lung	C1333123
combined lung carcinoma	C1333123
cribriform carcinoma of lung	C2138451
desmoplastic small round cell sarcoma of lung	C2182950
embryonal carcinosarcoma of lung	C2205906
embryonal rhabdomyosarcoma of lung	C2205919
embryonal sarcoma of lung	C2205898
epidermoid carcinoma of lung	C0149782
epidermoid cell carcinoma of lung	C0149782
epidermoid cell carcinoma of the lung	C0149782
epidermoid cell lung carcinoma	C0149782
epidermoid lung cancer	C0149782
epithelioid angioendothelioma of lung	C0345961
epithelioid angioendothelioma of the lung	C0345961
epithelioid haemangioendothelioma of lung	C0345961
epithelioid hemangioendothelioma of lung	C0345961
epithelioid hemangioendothelioma of the lung	C0345961
epithelioid sarcoma of lung	C2205895
fascial fibrosarcoma of lung	C2205916

fibromyxosarcoma of lung	C2205915
fibrosarcoma of lung	C2205905
fibrous histiocytoma of lung	C2205912
follicular dendritic cell sarcoma of lung	C2205897
fusiform type small cell carcinoma of lung	C2009882
giant cell and spindle cell carcinoma of lung	C2011219
giant cell carcinoma of lung	C0345960
giant cell carcinoma of the lung	C0345960
giant cell sarcoma of lung	C2011308
giant cell type neoplasm of lung	C2011382
glassy cell carcinoma of lung	C2012093
grade 1 follicular lymphoma of lung	C2200134
grade 1 nodular sclerosing hodgkin's lymphoma of lung	C2200121
grade 2 follicular lymphoma of lung	C2200135
grade 2 nodular sclerosing hodgkin's lymphoma of lung	C2200122
grade 3 follicular lymphoma of lung	C2200136
granular cell carcinoma of lung	C2012541
hemangiopericytoma of lung	C2030351
hemangiosarcoma of lung	C2030357
high grade lung neuroendocrine neoplasm	C1708364

© 2021 Yuan Q et al. *JAMA Network Open.*

hilar lung carcinoma	C133<br>4445
histiocytic sarcoma of lung	C204<br>6324
hodgkin's granuloma of lung	C204<br>6574
hodgkin's sarcoma of lung	C204<br>6714
infantile fibrosarcoma of lung	C220<br>0114
interdigitating dendritic cell sarcoma of lung	C207<br>7749
intravascular bronchoalveolar tumor	C034<br>5961
intravascular bronchoalveolar tumour	C034<br>5961
kaposi sarcoma of lung	C015<br>3564
kaposi's lung sarcoma	C015<br>3564
kaposi's lungs sarcoma	C015<br>3564
kaposi's sarcoma of lung	C015<br>3564
kaposi's sarcoma of the lung	C015<br>3564
langerhans cell sarcoma of lung	C211<br>1162
large cell cancer lung	C034<br>5958
large cell carcinoma lung	C034<br>5958
large cell carcinoma of lung	C034<br>5958
large cell carcinoma of the lung	C034<br>5958
large cell lung cancer	C034<br>5958
large cell lung carcinoma	C034<br>5958

large cell undifferentiated lung carcinoma	C034<br>5958
local recurrence of malignant neoplasm of lung	C128<br>2470
local recurrence of malignant tumor of lung	C128<br>2470
lung adenocarcinoma	C015<br>2013
lung adenocystic carcinoma	C133<br>4439
lung adenoid cystic carcinoma	C133<br>4439
lung atypical carcinoid tumor	C170<br>8766
lung biopsy malig lymphoma hodgkin's lymphocytic depletion diffuse fibrosis	C402<br>8795
lung biopsy malignant carcinoma large cell with rhabdoid phenotype	C402<br>8791
lung biopsy malignant carcinoma squamous cell with horn formation	C402<br>8790
lung biopsy malignant carcinoma transitional cell spindle cell	C402<br>8789
lung biopsy malignant lymphoma hodgkin's lymphocytic depletion	C402<br>8786
lung biopsy malignant lymphoma hodgkin's nodular sclerosis cellular phase	C402<br>8784
lung biopsy malignant lymphoma hodgkin's nodular sclerosis grade 1	C402<br>8783
lung biopsy malignant lymphoma hodgkin's nodular sclerosis grade 2	C402<br>8782
lung biopsy malignant lymphoma hodgkin's nodular sclerosis	C402<br>8785
lung biopsy malignant myosarcoma rhabdomyosarcoma embryonal	C402<br>8777
lung biopsy malignant myosarcoma rhabdomyosarcoma mixed type	C402<br>8776
lung biopsy malignant myosarcoma rhabdomyosarcoma spindle cell	C402<br>8775
lung biopsy malignant neoplasm adenocarcinoma hepatoid	C402<br>8770

© 2021 Yuan Q et al. *JAMA Network Open.*

lung biopsy malignant neoplasm carcinoid atypical	C402 8756
lung biopsy malignant neoplasm carcinoid composite	C402 8755
lung biopsy malignant neoplasm carcinoid enterochromaffin cell	C402 8754
lung biopsy malignant neoplasm carcinoid goblet cell	C402 8752
lung biopsy malignant neoplasm carcinoid	C402 8758
lung biopsy malignant neoplasm carcinoma acinar cell cystadenocarcinoma	C402 8749
lung biopsy malignant neoplasm carcinoma acinar cell	C402 8750
lung biopsy malignant neoplasm carcinoma adenoid cystic	C402 8748
lung biopsy malignant neoplasm carcinoma adenosquamous	C402 8747
lung biopsy malignant neoplasm carcinoma anaplastic	C402 8746
lung biopsy malignant neoplasm carcinoma basaloid	C402 8745
lung biopsy malignant neoplasm carcinoma cloacogenic	C402 8743
lung biopsy malignant neoplasm carcinoma cribriform	C402 8742
lung biopsy malignant neoplasm carcinoma epithelioma	C402 8740
lung biopsy malignant neoplasm carcinoma giant cell and spindle cell	C402 8738
lung biopsy malignant neoplasm carcinoma giant cell	C402 8739
lung biopsy malignant neoplasm carcinoma glassy cell	C402 8737
lung biopsy malignant neoplasm carcinoma granular cell	C402 8736
lung biopsy malignant neoplasm carcinoma large cell neuroendocrine	C402 8735
lung biopsy malignant neoplasm carcinoma medullary	C402 8734

lung biopsy malignant neoplasm carcinoma mucoepidermoid	C402 8733
lung biopsy malignant neoplasm carcinoma neuroendocrine	C402 8732
lung biopsy malignant neoplasm carcinoma papillary squamous cell	C402 8727
lung biopsy malignant neoplasm carcinoma papillary	C402 8728
lung biopsy malignant neoplasm carcinoma pleomorphic	C402 8725
lung biopsy malignant neoplasm carcinoma polygonal cell	C402 8724
lung biopsy malignant neoplasm carcinoma pseudosarcomatous	C402 8723
lung biopsy malignant neoplasm carcinoma schneiderian	C402 8722
lung biopsy malignant neoplasm carcinoma signet ring cell	C402 8721
lung biopsy malignant neoplasm carcinoma simplex	C402 8720
lung biopsy malignant neoplasm carcinoma small cell combined	C402 8719
lung biopsy malignant neoplasm carcinoma small cell fusiform cell	C402 8718
lung biopsy malignant neoplasm carcinoma small cell intermediate cell	C402 8717
lung biopsy malignant neoplasm carcinoma solid	C402 8716
lung biopsy malignant neoplasm carcinoma spindle cell	C402 8715
lung biopsy malignant neoplasm carcinoma squamous cell adenoid	C402 8714
lung biopsy malignant neoplasm carcinoma squamous cell keratinizing	C402 8713
lung biopsy malignant neoplasm carcinoma squamous cell spindle cell	C402 8712
lung biopsy malignant neoplasm carcinoma transitional cell	C402 8711
lung biopsy malignant neoplasm carcinoma undifferentiated	C402 8710

lung biopsy malignant neoplasm carcinoma verrucous	C4028709
lung biopsy malignant neoplasm carcinosarcoma embryonal	C4028707
lung biopsy malignant neoplasm carcinosarcoma myoepithelioma	C4028706
lung biopsy malignant neoplasm carcinosarcoma	C4028708
lung biopsy malignant neoplasm clear cell type	C4028704
lung biopsy malignant neoplasm epithelioid hemangioendothelioma	C4028703
lung biopsy malignant neoplasm fibrosarcoma fascial	C4028701
lung biopsy malignant neoplasm fibrosarcoma fibromyxosarcoma	C4028700
lung biopsy malignant neoplasm fibrosarcoma infantile	C4028699
lung biopsy malignant neoplasm fibrosarcoma solitary fibrous tumor	C4028698
lung biopsy malignant neoplasm fibrosarcoma	C4028702
lung biopsy malignant neoplasm fibrous histiocytoma	C4028697
lung biopsy malignant neoplasm giant cell type	C4028696
lung biopsy malignant neoplasm hemangiopericytoma	C4028695
lung biopsy malignant neoplasm lymphoma burkitt's	C4028692
lung biopsy malignant neoplasm lymphoma follicular grade 1	C4028690
lung biopsy malignant neoplasm lymphoma follicular grade 2	C4028689
lung biopsy malignant neoplasm lymphoma follicular grade 3	C4028688
lung biopsy malignant neoplasm lymphoma follicular	C4028691
lung biopsy malignant neoplasm lymphoma histiocytosis	C4028687
lung biopsy malignant neoplasm lymphoma hodgkin's granuloma	C4028685
lung biopsy malignant neoplasm lymphoma hodgkin's mixed cellularity	C4028683
lung biopsy malignant neoplasm lymphoma hodgkin's sarcoma	C4028682
lung biopsy malignant neoplasm lymphoma hodgkin's	C4028686
lung biopsy malignant neoplasm lymphoma lymphoplasmacytic	C4028680
lung biopsy malignant neoplasm lymphoma mantle cell	C4028679
lung biopsy malignant neoplasm lymphoma precursor cell lymphoblastic	C4028672
lung biopsy malignant neoplasm lymphoma primary effusion	C4028671
lung biopsy malignant neoplasm lymphoma	C4028694
lung biopsy malignant neoplasm mastocytosis	C4028669
lung biopsy malignant neoplasm mesenchymoma	C4028668
lung biopsy malignant neoplasm mesothelioma biphasic	C4028666
lung biopsy malignant neoplasm mesothelioma epithelioid	C4028665
lung biopsy malignant neoplasm mesothelioma fibrous	C4028664
lung biopsy malignant neoplasm mesothelioma	C4028667
lung biopsy malignant neoplasm myosarcoma angiomyosarcoma	C4028662
lung biopsy malignant neoplasm myosarcoma leiomyosarcoma epithelioid	C4028660
lung biopsy malignant neoplasm myosarcoma leiomyosarcoma myxoid	C4028659
lung biopsy malignant neoplasm myosarcoma leiomyosarcoma	C4028661
lung biopsy malignant neoplasm myosarcoma rhabdomyosarcoma	C4028658

© 2021 Yuan Q et al. *JAMA Network Open.*

lung biopsy malignant neoplasm myosarcoma	C402 8663
lung biopsy malignant neoplasm plasmacytoma extramedullary	C402 8655
lung biopsy malignant neoplasm plasmacytoma	C402 8656
lung biopsy malignant neoplasm pleuropulmonary blastoma	C402 8654
lung biopsy malignant neoplasm pulmonary blastoma	C402 8653
lung biopsy malignant neoplasm sarcoma embryonal	C402 8651
lung biopsy malignant neoplasm sarcoma epithelioid	C402 8650
lung biopsy malignant neoplasm sarcoma follicular dendritic cell	C402 8649
lung biopsy malignant neoplasm sarcoma giant cell	C402 8648
lung biopsy malignant neoplasm sarcoma hemangiosarcoma	C402 8647
lung biopsy malignant neoplasm sarcoma histiocytic	C402 8646
lung biopsy malignant neoplasm sarcoma kaposi's	C402 8645
lung biopsy malignant neoplasm sarcoma langerhans cell	C402 8644
lung biopsy malignant neoplasm sarcoma mast cell	C402 8643
lung biopsy malignant neoplasm sarcoma small cell	C402 8642
lung biopsy malignant neoplasm sarcoma spindle cell	C402 8641
lung biopsy malignant neoplasm sarcoma undifferentiated	C402 8640
lung biopsy malignant neoplasm sarcoma	C402 8652
lung biopsy malignant neoplasm small cell type	C402 8639
lung biopsy malignant neoplasm spindle cell type	C402 8638

lung biopsy malignant sarcoma desmoplastic small round cell	C402 8637
lung biopsy malignant sarcoma interdigitating dendritic cell	C402 8636
lung blastoma	C020 6629
lung cancer adenocarcinoma	C015 2013
lung cancer adenosquamous	C027 9557
lung cancer bronchogenic carcinoma	C000 7121
lung cancer large cell	C034 5958
lung cancer malignant	C024 2379
lung cancer metastatic	C015 3676
lung cancer non small cell	C000 7131
lung cancer oat cell	C014 9925
lung cancer small cell	C014 9925
lung cancer squamous cell	C014 9782
lung cancers	C024 2379
lung cancer	C024 2379
lung cancer	C068 4249
lung cancer	C130 6460
lung carcinoid tumor	C028 0089
lung carcinoma by ajcc v6 stage	C298 3716
lung carcinoma by ajcc v7 stage	C298 4096

© 2021 Yuan Q et al. *JAMA Network Open.*

lung carcinoma cell type unspecified recurrent	C085 5002
lung carcinoma non small cell	C000 7131
lung carcinoma	C068 4249
lung carcinosarcoma	C171 1276
lung epithelioid angioendothelioma	C034 5961
lung epithelioid hemangioendothelioma	C034 5961
lung giant cell carcinoma	C034 5960
lung hilum carcinoma	C133 4445
lung kaposi sarcoma	C015 3564
lung kaposi's sarcoma	C015 3564
lung lymphoma	C170 4383
lung malignancies	C024 2379
lung malignancy	C024 2379
lung malignant carcinoid tumor	C170 8766
lung malignant carcinoma clear cell	C034 5959
lung malignant carcinoma oat cell	C014 9925
lung malignant carcinoma of lower lobe or bronchus	C129 9242
lung malignant carcinoma of middle lobe or bronchus	C129 9244
lung malignant carcinoma of upper lobe or bronchus	C129 9257
lung malignant carcinoma schneiderian	C220 5885

lung malignant neoplasm primary bilateral	C386 2555
lung malignant neoplasm primary left	C386 2552
lung malignant neoplasm primary right	C386 2551
lung malignant neoplasm primary	C130 6460
lung malignant tumors	C024 2379
lung malignant tumours	C024 2379
lung melanoma	C170 8773
lung metastases	C015 3676
lung metastasis	C015 3676
lung mucoepidermoid carcinoma	C170 8778
lung neoplasm malignant bronchus secondary left lower lobe	C068 5052
lung neoplasm malignant bronchus secondary right lower lobe	C068 5047
lung neoplasm malignant bronchus secondary right middle lobe	C068 5042
lung neoplasm malignant bronchus	C132 2284
lung neoplasm malignant carcinoma of paranchyema	C034 5957
lung neoplasm malignant clear cell type	C207 5625
lung neoplasm malignant giant cell type	C201 1382
lung neoplasm malignant hilus primary	C034 6601
lung neoplasm malignant left lower lobe primary	C068 5077
lung neoplasm malignant left lower lobe	C068 5077

lung neoplasm malignant left upper lobe primary	C068 5072
lung neoplasm malignant left upper lobe	C068 5072
lung neoplasm malignant middle lobe of bronchus	C034 6603
lung neoplasm malignant middle lobe right primary	C068 5062
lung neoplasm malignant middle lobe right	C068 5062
lung neoplasm malignant myosarcoma rhabdomyosarcoma	C220 0351
lung neoplasm malignant parenchyma	C034 5955
lung neoplasm malignant rhabdomyosarcoma embryonal	C220 5919
lung neoplasm malignant rhabdomyosarcoma mixed type	C220 5918
lung neoplasm malignant rhabdomyosarcoma spindle cell	C201 8439
lung neoplasm malignant right lower lobe primary	C068 5067
lung neoplasm malignant right lower lobe	C068 5067
lung neoplasm malignant right upper lobe primary	C068 5057
lung neoplasm malignant right upper lobe	C068 5057
lung neoplasm malignant sarcoma desmoplastic small round cell	C218 2950
lung neoplasm malignant sarcoma kaposi's	C015 3564
lung neoplasm malignant small cell type	C220 5877
lung neoplasm malignant spindle cell type	C201 8665
lung neoplasm malignant squamous cell carcinoma of bronchus	C131 4696
lung neoplasm malignant susceptibility	C383 6906

lung neoplasm malignant	C024 2379
lung pleomorphic carcinoma	C171 1397
lung sarcomas	C059 8790
lung sarcoma	C059 8790
lung secondaries	C015 3676
lung secondary	C015 3676
lung signet ring cell carcinoma	C170 8782
lung small cell neuroendocrine carcinoma	C014 9925
lung spindle cell carcinoma	C170 8784
lung squamous cell carcinoma	C014 9782
lungs cancer	C024 2379
lungs metastasis	C015 3676
lungs sarcoma	C059 8790
lymphoma involving lung	C051 9063
lymphoma involving the lung	C051 9063
lymphoma of lung	C170 4383
lymphoma of the lung	C170 4383
lymphoplasmacytic lymphoma of lung	C220 0140
malignant biphasic mesothelioma of lung	C220 5924
malignant carcinoid tumor of lung	C170 8766

© 2021 Yuan Q et al. *JAMA Network Open.*

malignant chemodectoma of lung	C204<br>5776
malignant epithelial neoplasm of lung	C316<br>3822
malignant epithelioid hemangioendothelioma of lung	C034<br>5961
malignant epithelioid mesothelioma of lung	C220<br>5923
malignant epithelioma of lung	C220<br>5878
malignant fibrous mesothelioma of lung	C220<br>5922
malignant hemangiopericytoma of lung	C203<br>0351
malignant hilar lung neoplasm	C260<br>7931
malignant hilar lung tumor	C260<br>7931
malignant histiocytosis of lung	C220<br>0137
malignant lung hilum neoplasm	C260<br>7931
malignant lung hilum tumor	C260<br>7931
malignant lung neoplasm	C024<br>2379
malignant lung tumor	C024<br>2379
malignant lymphoma of lung	C220<br>5908
malignant mastocytosis of lung	C220<br>5913
malignant mesenchymoma of lung	C220<br>5920
malignant mesothelioma of lung	C220<br>5921
malignant myoepithelioma of lung	C220<br>5907
malignant neoplasm bronchus/lung	C034<br>8343

malignant neoplasm bronchus	C000<br>7121
malignant neoplasm lung	C024<br>2379
malignant neoplasm of bronchus carcinoma	C000<br>7121
malignant neoplasm of bronchus	C132<br>2284
malignant neoplasm of carina of lung	C200<br>4495
malignant neoplasm of carina	C200<br>4495
malignant neoplasm of hilus of lung	C034<br>6601
malignant neoplasm of hilus of lung	C260<br>7931
malignant neoplasm of left lower lobe of lung	C068<br>5077
malignant neoplasm of left upper lobe of lung	C068<br>5072
malignant neoplasm of lower lobe of lung	C034<br>6604
malignant neoplasm of lung hilum	C260<br>7931
malignant neoplasm of lung	C024<br>2379
malignant neoplasm of middle lobe bronchus	C034<br>6603
malignant neoplasm of middle lobe of bronchus	C034<br>6603
malignant neoplasm of middle lobe of lung	C034<br>6602
malignant neoplasm of other parts of bronchus or lung	C015<br>3493
malignant neoplasm of overlapping lesion of bronchus and lung	C034<br>9043
malignant neoplasm of overlapping sites of bronchus and lung	C034<br>9043
malignant neoplasm of parietal pleura of lung	C200<br>4481

© 2021 Yuan Q et al. *JAMA Network Open.*

malignant neoplasm of parietal pleura	C200 4481
malignant neoplasm of right lower lobe of lung	C068 5067
malignant neoplasm of right middle lobe of lung	C068 5062
malignant neoplasm of right upper lobe of lung	C068 5057
malignant neoplasm of the lung hilum	C260 7931
malignant neoplasm of the lung	C024 2379
malignant neoplasm of upper lobe of lung	C034 6600
malignant neoplasm of visceral pleura of lung	C260 7950
malignant neoplasm of visceral pleura	C260 7950
malignant neosplasm of the bronchus	C132 2284
malignant neosplasm of the lung	C024 2379
malignant plasmacytoma of lung	C220 5910
malignant small cell neoplasm of lung	C220 5877
malignant solitary fibrous tumor of lung	C220 0149
malignant superior sulcus lung neoplasm	C054 9471
malignant superior sulcus lung tumor	C054 9471
malignant superior sulcus neoplasm of lung	C054 9471
malignant superior sulcus neoplasm of the lung	C054 9471
malignant superior sulcus neoplasm	C054 9471
malignant superior sulcus tumor of lung	C054 9471

malignant superior sulcus tumor of the lung	C054 9471
malignant superior sulcus tumor	C054 9471
malignant tumor of bronchus	C132 2284
malignant tumor of carina of lung	C200 4495
malignant tumor of hilus of lung	C260 7931
malignant tumor of lower lobe of lung	C034 6604
malignant tumor of lung hilum	C260 7931
malignant tumor of lung parenchyma	C034 5955
malignant tumor of lung	C024 2379
malignant tumor of middle lobe of lung	C034 6602
malignant tumor of parietal pleura of lung	C200 4481
malignant tumor of the lung hilum	C260 7931
malignant tumor of the lung	C024 2379
malignant tumor of upper lobe of lung	C034 6600
malignant tumor of visceral pleura of lung	C260 7950
malignant tumour of bronchus	C132 2284
malignant tumour of lung parenchyma	C034 5955
malignant tumour of lung	C024 2379
mantle cell lymphoma of lung	C220 0141
mast cell sarcoma of lung	C220 5896

© 2021 Yuan Q et al. *JAMA Network Open.*

medullary carcinoma of lung	C220 5883
metastases to lung	C015 3676
metastasis from adenocarcinoma of lung	C349 4606
metastasis from malignant neoplasm of lung	C128 2508
metastasis from malignant tumor of lung	C128 2508
metastasis lung	C015 3676
metastasis of malignant neoplasm to lung	C015 3676
metastasis to lung from adenocarcinoma	C349 4606
metastasis to lung	C015 3676
metastasis to the lung	C015 3676
metastatic cancer lung	C015 3676
metastatic cancer to lung	C015 3676
metastatic carcinoma in the lung	C023 8254
metastatic carcinoma to the lung	C023 8254
metastatic lung cancer	C015 3676
metastatic malignant neoplasm in the lung	C015 3676
metastatic malignant neoplasm to bronchus of left lower lobe	C068 5052
metastatic malignant neoplasm to bronchus of right lower lobe	C068 5047
metastatic malignant neoplasm to bronchus of right middle lobe	C068 5042
metastatic malignant neoplasm to lung	C015 3676

metastatic malignant neoplasm to the lung	C015 3676
metastatic neoplasm to the lung	C015 3676
metastatic tumor to the lung	C015 3676
mixed cellularity hodgkin's lymphoma of lung	C220 0118
mixed type rhabdomyosarcoma of lung	C220 5918
mucoepidermoid carcinoma of lung	C170 8778
mucoepidermoid carcinoma of lung	C385 4329
myosarcoma of lung	C220 5899
neuroendocrine carcinoma of lung	C220 0127
nodular sclerosing hodgkin's lymphoma in cellular phase of lung	C220 0120
nodular sclerosing hodgkin's lymphoma of lung	C220 0119
non small cell lung cancer nos	C000 7131
non small cell lung cancer	C000 7131
non small cell lung carcinoma	C000 7131
nonsmall cell lung cancer	C000 7131
nsclca	C000 7131
nsclc	C000 7131
oat cell cancer	C014 9925
oat cell carcinoma of lung	C014 9925
oat cell lung cancer	C014 9925

© 2021 Yuan Q et al. *JAMA Network Open.*

occult carcinoma of lung	C133 5097
occult carcinoma of the lung	C133 5097
occult lung carcinoma	C133 5097
of carcinoid lung tumor	C028 0089
of lung adenocarcinoma	C015 2013
of lung carcinoma	C068 4249
overlapping malignant lesion of bronchus and lung	C034 9043
overlapping malignant neoplasm of bronchus and lung	C034 9043
pancoast tumor of lung	C054 9471
pancoast tumors	C054 9471
pancoast tumor	C054 9471
pancoast tumour	C054 9471
pancoast's tumor	C054 9471
pancoast's tumour	C054 9471
papillary carcinoma of lung	C203 3219
papillary squamous cell carcinoma of lung	C203 3296
papillary squamous cell carcinoma of lung	C385 4249
pleomorphic carcinoma of lung	C171 1397
pleomorphic carcinoma of lung	C385 4292
pleuropulmonary blastoma of lung	C208 2572

pleuropulmonary blastoma	C126 6144
pneumoblastoma	C020 6629
polygonal cell carcinoma of lung	C211 1806
precursor cell lymphoblastic lymphoma of lung	C211 3706
primary acinar cell carcinoma of lung	C387 3379
primary adenocarcinoma of lung	C387 3341
primary basaloid squamous cell carcinoma of lung	C387 3339
primary bronchial cancer	C000 7121
primary carcinosarcoma of lung	C387 3261
primary clear cell squamous cell carcinoma of lung	C387 3340
primary effusion lymphoma of lung	C220 0147
primary lung lymphoma	C170 4383
primary malignant neoplasm of bilateral lungs	C386 2555
primary malignant neoplasm of hilus of lung	C034 6601
primary malignant neoplasm of left lower lobe of lung	C068 5077
primary malignant neoplasm of left lung	C386 2552
primary malignant neoplasm of left upper lobe of lung	C068 5072
primary malignant neoplasm of lower lobe of left lung	C068 5077
primary malignant neoplasm of lung	C130 6460
primary malignant neoplasm of right lower lobe of lung	C068 5067

© 2021 Yuan Q et al. *JAMA Network Open.*

primary malignant neoplasm of right lung	C386 2551
primary malignant neoplasm of right middle lobe of lung	C068 5062
primary malignant neoplasm of right upper lobe of lung	C068 5057
primary malignant neoplasm of upper lobe of left lung	C068 5072
primary malignant neoplasm of upper lobe of right lung	C068 5057
primary malignant neuroendocrine neoplasm of lung	C430 2468
primary mucoepidermoid carcinoma of lung	C385 4329
primary papillary squamous cell carcinoma of lung	C385 4249
primary pleomorphic carcinoma of lung	C385 4292
primary pseudosarcomatous carcinoma of lung	C387 3337
primary pulmonary lymphoma	C427 3669
primary signet ring cell carcinoma of lung	C387 3362
primary spindle cell carcinoma of lung	C387 3338
primary undifferentiated carcinoma of lung	C385 4184
protection against malignant lung neoplasm	C383 6305
pseudosarcomatous carcinoma of lung	C214 2924
pulmonary adenocarcinoma	C015 2013
pulmonary adenocystic carcinoma	C133 4439
pulmonary adenoid cystic carcinoma	C133 4439
pulmonary blastoma of childhood	C126 6144

pulmonary blastoma of lung	C206 2937
pulmonary blastomas	C020 6629
pulmonary blastoma	C020 6629
pulmonary cancer	C024 2379
pulmonary carcinoid tumor	C028 0089
pulmonary carcinoma	C068 4249
pulmonary epithelioid angioendothelioma	C034 5961
pulmonary epithelioid hemangioendothelioma	C034 5961
pulmonary kaposi sarcoma	C015 3564
pulmonary kaposi's sarcoma	C015 3564
pulmonary lymphoma	C051 9063
pulmonary lymphoma	C170 4383
pulmonary metastases	C015 3676
pulmonary metastasis	C015 3676
pulmonary sarcoma	C059 8790
pulmonary secondary	C015 3676
pulmonary small cell carcinoma oat cell	C014 9925
recurrent lung cancer	C085 5002
recurrent lung carcinoma cell type unspecified	C085 5002
recurrent lung carcinoma	C085 5002

recurrent unspecified carcinoma of lung	C0855002
recurrent unspecified carcinoma of the lung	C0855002
recurrent unspecified lung carcinoma	C0855002
relapsed unspecified carcinoma of lung	C0855002
relapsed unspecified carcinoma of the lung	C0855002
relapsed unspecified lung carcinoma	C0855002
rhabdomyosarcoma of lung	C2200351
sarcoma lung	C0598790
sarcoma of lung	C0598790
sarcoma of the lung	C0598790
sarcomatoid carcinoma of lung	C3873337
schneiderian carcinoma of lung	C2205885
sclc	C0149925
secondary cancer lung	C0153676
secondary cancer of lung	C0153676
secondary lung cancer	C0153676
secondary malignant neoplasm of bronchus of left lower lobe	C0685052
secondary malignant neoplasm of bronchus of right lower lobe	C0685047
secondary malignant neoplasm of bronchus of right middle lobe	C0685042
secondary malignant neoplasm of lung	C0153676

secondary malignant tumor of lung	C0153676
secondary malignant tumour of lung	C0153676
sezary syndrome of lung	C2205925
signet ring cell carcinoma of lung	C1708782
signet ring cell carcinoma of lung	C3873362
scc	C0149925
oat cell cancer	C0262584
oat cell carcinoma	C0262584
oat cell carcinoma syndrome	C0262584
oat cell carcinomas	C0262584
reserve cell carcinoma	C0262584
round cell carcinoma	C0262584
sclc	C0262584
small cell cancer	C0262584
small cell carcinoma	C0262584
small cell carcinoma nos	C0262584
small cell carcinomas	C0262584
small cell nec	C0262584
small cell neuroendocrine carcinoma	C0262584
small cell cancer of the lung	C0149925

© 2021 Yuan Q et al. *JAMA Network Open.*

small cell carcinoma of lung	C014 9925
small cell carcinoma of the lung	C014 9925
small cell carcinoma	C014 9925
small cell lung cancer	C014 9925
small cell lung carcinoma	C014 9925
small cell neuroendocrine carcinoma of lung	C014 9925
small cell neuroendocrine carcinoma of the lung	C014 9925
small cell sarcoma of lung	C220 5894
small non cell lung cancer	C000 7131
solid carcinoma of lung	C201 7450
spindle cell carcinoma of lung	C170 8784
spindle cell carcinoma of lung	C387 3338
spindle cell rhabdomyosarcoma of lung	C201 8439
spindle cell sarcoma of lung	C201 8494
spindle cell type neoplasm of lung	C201 8665
squamous cell cancer lung	C014 9782

squamous cell carcinoma lung	C014 9782
squamous cell carcinoma of bronchus	C131 4696
squamous cell carcinoma of lung	C014 9782
squamous cell carcinoma of the lung	C014 9782
squamous cell lung cancer	C014 9782
squamous cell lung carcinoma	C014 9782
superior sulcus tumor	C054 9471
superior sulcus tumour	C054 9471
terminal bronchiolar carcinoma	C000 7120
transitional cell carcinoma of lung	C214 5459
undifferentiated carcinoma of lung	C206 2537
undifferentiated carcinoma of lung	C385 4184
undifferentiated large cell lung cancer	C034 5958
undifferentiated sarcoma of lung	C218 8131
verrucous carcinoma of lung	C218 9349

2. CUIs for other cancers

Term	CUI
bed sore	C0011127
bed sores	C0011127
bedsore	C0011127
bedsores	C0011127
chronic cutaneous pressure ulcer back	C0558155
chronic cutaneous pressure ulcer buttock and hip	C2888461
chronic cutaneous pressure ulcer elbow	C0558156
chronic cutaneous pressure ulcer head	C0558154
chronic cutaneous pressure ulcer heel	C0558158
chronic cutaneous ulcer decubitus healing	C2368046
chronic decubitus	C3815112
chronic decubitus ulcer	C0011127
chronic decubitus ulcer	C3815112
chronic decubitus ulcer stage	C1718233
chronic pressure sore	C3815112
chronic pressure ulcer	C3815112
contact ulcer	C0011127
contact ulcers	C0011127
decubital ulcer of coccygeal region	C1997878
decubiti	C0011127
decubitus	C0011127
decubitus gangrene	C0343044
decubitus heel ulcers	C0558158
decubitus pressure sore	C0011127
decubitus skin ulcer	C0011127
decubitus skin ulcers	C0011127
decubitus ulcer	C0011127
decubitus ulcer of ankle	C0577713
decubitus ulcer of breast	C0577714
decubitus ulcer of buttock	C0558160
decubitus ulcer of dorsum of foot	C0577716
decubitus ulcer of heel	C0558158
decubitus ulcer of hip	C0577712
decubitus ulcer of left ankle	C2074656
decubitus ulcer of left hip	C2074660
decubitus ulcer of lower back	C1456141
decubitus ulcer of natal cleft	C0577715
decubitus ulcer of right ankle	C2074661
decubitus ulcer of right hip	C2074665
decubitus ulcer of sacrum	C0558159
decubitus ulcer of shoulder	C0558152
decubitus ulcer of upper back	C1456139
decubitus ulcers	C0011127
deep and superficial pressure sore	C0429996
deep and superficial pressure ulcer	C0429996
deep pressure sore	C0429995
deep pressure ulcer	C0429995
gangrenous pressure sore	C0343044
gangrenous pressure ulcer	C0343044
healing chronic decubitus ulcer	C2368046
heels pressure sores	C0558158
hospice acquired pressure ulcer	C2960067
hospital acquired pressure ulcer	C2959937
nonhospital acquired pressure ulcer	C4551355
nonstageable pressure ulcer	C1720363
nursing home acquired pressure ulcer	C2960681
plaster ulcer	C0263559
pressure injury stage 1	C1720599
pressure injury stage 2	C1720518
pressure injury stage 3	C1719811
pressure injury stage 4	C1719910
pressure sore	C0011127
pressure sore of buttock	C0558160
pressure sore on ankle	C0558157
pressure sore on back	C0558155
pressure sore on buttocks	C0558160
pressure sore on ear	C0558153
pressure sore on elbow	C0558156
pressure sore on head	C0558154
pressure sore on heel	C0558158
pressure sore on keel	C3687383
pressure sore on malleolus	C0558157
pressure sore on sacrum	C0558159
pressure sore on shoulder	C0558152
pressure sore or ulcer	C0011127
pressure sores	C0011127
pressure sores/ulcers	C0011127
pressure ulcer	C0011127
pressure ulcer caused by device	C4041246
pressure ulcer due to device	C4041246
pressure ulcer due to spina bifida	C4040708
pressure ulcer of ankle	C0577713
pressure ulcer of back	C0558155
pressure ulcer of breast	C0577714
pressure ulcer of buttock	C0558160
pressure ulcer of coccygeal region	C1997878
pressure ulcer of dorsum of foot	C0577716
pressure ulcer of ear	C0558153
pressure ulcer of elbow	C0558156
pressure ulcer of foot	C4082071
pressure ulcer of head	C0558154
pressure ulcer of heel	C0558158
pressure ulcer of hip	C0577712
pressure ulcer of knee	C3164536
pressure ulcer of left ankle	C2074656
pressure ulcer of left hip	C2074660
pressure ulcer of left leg	C4047492
pressure ulcer of left lower limb	C4047492
pressure ulcer of lower back	C1456141
pressure ulcer of malleolus	C0558157
pressure ulcer of natal cleft	C0577715
pressure ulcer of other site	C2888580
pressure ulcer of right ankle	C2074661
pressure ulcer of right hip	C2074665
pressure ulcer of right lower extemity	C4082060
pressure ulcer of right lower limb	C4082060
pressure ulcer of sacral region	C0558159
pressure ulcer of sacrum	C0558159
pressure ulcer of shoulder	C0558152
pressure ulcer of the hip	C0577712
pressure ulcer of unspecified site	C0011127

pressure ulcer of upper back	C1456139
pressure ulcer on back	C0558155
pressure ulcer on ear	C0558153
pressure ulcer on elbow	C0558156
pressure ulcer on head	C0558154
pressure ulcer on heel	C0558158
pressure ulcer on malleolus	C0558157
pressure ulcer on sacrum	C0558159
pressure ulcer on shoulder	C0558152
pressure ulcer stage	C1718233
pressure ulcer stage 1	C1720599
pressure ulcer stage 2	C1720518
pressure ulcer stage 3	C1719811
pressure ulcer stage 4	C1719910
pressure ulcer stages	C1718233
pressure ulcers	C0011127
sacral pressure core	C0558159
sacral pressure sore	C0558159
stage i decubitus ulcer	C2074666
stage i pressure ulcer	C2074666
stage ii decubitus ulcer	C2074667
stage ii pressure ulcer	C2074667
stage iii decubitus ulcer	C2074668
stage iii pressure ulcer	C2074668
stage iv decubitus ulcer	C2074669
stage iv pressure ulcer	C2074669
stage v decubitus ulcer	C2074670
stage vi decubitus ulcer	C2074671
superficial pressure sore	C0429994
superficial pressure ulcer	C0429994
unstageable decubitus ulcer	C2368027
unstageable pressure ulcer	C2368027
malignant spindle cell neoplasm	C0334230
malignant spindle cell tumor	C0334230
penile neoplasm malignant spindle cell type	C1336052
spindle cell cancer	C0334230
spindle cell neoplasm	C1336052
spindle cell neoplasm of penis	C1336052
spindle cell tumor	C1336052
spindle cell type neoplasm	C0334230
curling ulcer	C2741638
decreased pressure	C0231299
decreased stress	C0231299
difficulty managing stress	C0237203
emotional stress	C0086209
feeling stressed	C0564404
fetal stress	C0456057
fetal stress disorder	C0456057
foetal stress	C0456057
increased pressure	C0231298
increased stress	C0231298
job strain	C2985217
life stress	C0038443
life stresses	C0038443
mental stress	C0038443
muscle stress	C0596987
muscle stressed	C0596987
muscles stress	C0596987

organic stress	C0683136
out stress	C0564404
physical stress	C0231302
physiol stress	C0449430
physiological stress	C0449430
physiological stresses	C0449430
prenatal stress	C0456057
psychol stress	C0038443
psychologic stress	C0038443
psychological stress	C0038443
psychological stresses	C0038443
psychological stressor	C0038443
psychological stressors	C0038443
reaction to severe stress	C3646472
recent emotional stress from a physical disability	C2020634
recent emotional stress from a serious illness	C2020635
recent emotional stress from chronic pain	C2020637
serious illness	C2020635
situational stress related to housing	C0281978
situational stress related to illness	C0281979
skeletal stress	C0542272
state of stress	C0038435
stress	C0038435
stress	C0723460
stress	C1718621
stress associated with a physical disability	C2020634
stress associated with physical disability	C2020634
stress associated with serious illness	C2020635
stress bismuth subsalicylate	C0723460
stress brand	C0723460
stress due to family tension	C4076073
stress emotional	C0086209
stress from financial loss	C2020675
stress induced peptic ulcer	C2741638
stress mental	C0038443
stress muscle	C0596987
stress muscles	C0596987
stress out	C0564404
stress physical	C0231302
stress physiol	C0449430
stress physiological	C0449430
stress psychol	C0038443
stress symptom	C0521991
stress symptoms	C0521991
stress ulcer	C2741638
stress ulcer nos	C2741638
stress ulcers	C2741638
stressed	C0038435
stressed out	C0564404
stressor	C0597530
stressors	C0597530
symptoms of stress	C0521991
symptoms stress	C0521991
tension reduction theory of aodu	C0678340
ulcer stress	C2741638
w stress	C1718621
bladder ca	C0005684

© 2021 Yuan Q et al. *JAMA Network Open.*

bladder cancer	C0005684
bladder cancer malignant	C0005684
bladder cancer nos	C0005684
bladder cancers	C0005684
bladder neoplasms malignant	C0005684
bladder--cancer	C0005684
ca - bladder cancer	C0005684
ca bladder	C0005684
cancer of bladder	C0005684
cancer of the bladder	C0005684
malig neo bladder nos	C0005684
malig neop bladder nos	C0005684
malig neop of bladder	C0005684
malign tumor urinary bladder	C0005684
malign tumour urinary bladder	C0005684
malignant bladder neoplasm	C0005684
malignant bladder tumor	C0005684
malignant neoplasm of bladder	C0005684
malignant neoplasm of the bladder	C0005684
malignant neoplasm of the urinary bladder	C0005684
malignant neoplasm of urinary bladder	C0005684
malignant neoplasm of urinary bladder nos	C0005684
malignant neosplasm of the bladder	C0005684
malignant tumor of bladder	C0005684
malignant tumor of the bladder	C0005684
malignant tumor of the urinary bladder	C0005684
malignant tumor of urinary bladder	C0005684
malignant tumour of urinary bladder	C0005684
malignant urinary bladder neoplasm	C0005684
malignant urinary bladder tumor	C0005684
urinary bladder cancer	C0005684
urinary bladder malignant neoplasm	C0005684
urinary bladder malignant tumor	C0005684
bladder cancer	C0005695
bladder neopl	C0005695
bladder neoplasm	C0005695
bladder neoplasm nos	C0005695
bladder neoplasm urinary	C0005695
bladder neoplasms	C0005695
bladder tumor	C0005695
bladder tumors	C0005695
bladder--tumors	C0005695
neopl bladder	C0005695
neoplasm bladder	C0005695
neoplasm of bladder	C0005695
neoplasm of the bladder	C0005695
neoplasm of the urinary bladder	C0005695
neoplasm of urinary bladder	C0005695
neoplasm urinary bladder	C0005695
ngb - new growth of bladder	C0005695
tumor bladder	C0005695
tumor of bladder	C0005695
tumor of the bladder	C0005695
tumor of the urinary bladder	C0005695
tumor of urinary bladder	C0005695
tumour of urinary bladder	C0005695
urinary bladder neopl	C0005695
urinary bladder neoplasm	C0005695
urinary bladder neoplasms	C0005695

urinary bladder tumor	C0005695
urinary bladder tumors	C0005695
brain neopl	C0006118
brain neoplasm	C0006118
brain neoplasm nos	C0006118
brain neoplasms	C0006118
brain tumor	C0006118
brain tumors	C0006118
brain tumour	C0006118
brain tumours	C0006118
neopl brain	C0006118
neoplasm of brain	C0006118
neoplasm of the brain	C0006118
neoplasm of unspecified nature of brain	C0006118
tumor of brain	C0006118
tumor of the brain	C0006118
breast cancer	C0006142
breast cancers	C0006142
breast malignant neoplasm	C0006142
breast malignant neoplasms	C0006142
breast malignant tumor	C0006142
breast malignant tumors	C0006142
cancer of breast	C0006142
cancer of the breast	C0006142
malignant breast neoplasm	C0006142
malignant breast tumor	C0006142
malignant neoplasm of breast	C0006142
malignant neoplasm of the breast	C0006142
malignant tumor of breast	C0006142
malignant tumor of the breast	C0006142
malignant tumour of breast	C0006142
mammary cancer	C0006142
mammary cancers	C0006142
b all	C0006413
burkitt cell leukaemia	C0006413
burkitt cell leukemia	C0006413
burkitt leukemia	C0006413
burkitt lymphoma	C0006413
burkitt lymphoma/leukaemia	C0006413
burkitt lymphoma/leukemia	C0006413
burkitt lymphomas	C0006413
burkitt tumor	C0006413
burkitt's cell leukaemia	C0006413
burkitt's cell leukemia	C0006413
burkitt's leukaemia	C0006413
burkitt's leukemia	C0006413
burkitt's lymphoma	C0006413
burkitt's lymphoma nos	C0006413
burkitt's lymphoma of unspecified site	C0006413
burkitt's tumor	C0006413
burkitt's tumor or lymphoma	C0006413
burkitt's tumour	C0006413
burkitts leukemia	C0006413
burkitts lymphoma	C0006413
burkitts tumor	C0006413
fab l3	C0006413
l3 acute lymphoblastic leukemia	C0006413
l3 acute lymphocytic leukemia	C0006413
l3 acute lymphogenous leukemia	C0006413

l3 acute lymphoid leukemia	C0006413
l3 lymphocytic leukemia	C0006413
l3 lymphocytic leukemias	C0006413
leukemia burkitt cell	C0006413
carcinoid	C0007095
carcinoid tumor	C0007095
carcinoid tumor nos	C0007095
carcinoid tumors	C0007095
carcinoid tumour	C0007095
carcinoid tumours	C0007095
carcinoids	C0007095
cancer of endometrium	C0007103
cancer of the endometrium	C0007103
endometrial cancer	C0007103
endometrial neoplasm malignant	C0007103
malignant endometrial neoplasm	C0007103
malignant neoplasm of endometrium	C0007103
malignant neoplasm of the endometrium	C0007103
ca larynx	C0007107
cancer laryngeal	C0007107
cancer larynx	C0007107
cancer of larynx	C0007107
cancer of the larynx	C0007107
laryngeal cancer	C0007107
laryngeal cancers	C0007107
larynx cancer	C0007107
larynx cancers	C0007107
larynx neoplasm malignant	C0007107
malignant laryngeal neoplasm	C0007107
malignant laryngeal tumor	C0007107
malignant larynx neoplasm	C0007107
malignant larynx tumor	C0007107
malignant neo larynx nos	C0007107
malignant neoplasm of larynx	C0007107
malignant neoplasm of larynx nos	C0007107
malignant neoplasm of the larynx	C0007107
malignant neosplasm of the larynx	C0007107
malignant tumor of larynx	C0007107
malignant tumor of the larynx	C0007107
malignant tumour of larynx	C0007107
of larynx cancer	C0007107
ca rectum	C0007113
cancer of rectum	C0007113
cancer of the rectum	C0007113
carcinoma of rectum	C0007113
carcinoma of the rectum	C0007113
carcinoma rectum	C0007113
malignant neoplasm of rectum	C0007113
rectal cancer	C0007113
rectal cancers	C0007113
rectal carcinoma	C0007113
rectal neoplasm malignant carcinoma	C0007113
rectum cancer	C0007113
rectum cancers	C0007113
rectum carcinoma	C0007113
cancer of skin	C0007114
cancer of the skin	C0007114
cancer skin	C0007114
cancers skin	C0007114

malignant neoplasm of skin	C0007114
malignant neoplasm of skin nos	C0007114
malignant neoplasm of the skin	C0007114
malignant neoplasm skin	C0007114
malignant neoplasm skin nos	C0007114
malignant neosplasm of the skin	C0007114
malignant skin neoplasm	C0007114
malignant skin tumor	C0007114
malignant tumor of skin	C0007114
malignant tumor of the skin	C0007114
malignant tumour of skin	C0007114
of skin cancer	C0007114
of the skin cancer	C0007114
skin cancer	C0007114
skin cancers	C0007114
skin neoplasm malignant	C0007114
cancer of the thyroid	C0007115
cancer of thyroid	C0007115
malign neopl thyroid	C0007115
malignant neoplasm of the thyroid	C0007115
malignant neoplasm of the thyroid gland	C0007115
malignant neoplasm of thyroid	C0007115
malignant neoplasm of thyroid gland	C0007115
malignant neoplasm thyroid	C0007115
malignant neosplasm of the thyroid gland	C0007115
malignant thyroid gland neoplasm	C0007115
malignant thyroid gland tumor	C0007115
malignant thyroid neoplasm	C0007115
malignant thyroid tumor	C0007115
malignant tumor of the thyroid	C0007115
malignant tumor of the thyroid gland	C0007115
malignant tumor of thyroid	C0007115
malignant tumor of thyroid gland	C0007115
malignant tumour of thyroid gland	C0007115
thyroid ca	C0007115
thyroid cancer	C0007115
thyroid cancers	C0007115
thyroid gland cancer	C0007115
thyroid neoplasm malignant	C0007115
basal cell cancer	C0007117
basal cell carcinoma	C0007117
basal cell carcinoma nos	C0007117
basal cell carcinoma of skin	C0007117
basal cell carcinoma of the skin	C0007117
basal cell carcinomas	C0007117
basal cell epithelioma	C0007117
basal cell epitheliomas	C0007117
basal cell skin cancer	C0007117
basal cell skin carcinoma	C0007117
basalioma	C0007117
basiloma	C0007117
bcc	C0007117
epithelioma basal cell	C0007117
malignant neoplasm carcinoma epithelioma basal cell	C0007117
rodent ulcer	C0007117
rodent ulcers	C0007117
skin basal cell cancer	C0007117
skin basal cell carcinoma	C0007117

© 2021 Yuan Q et al. *JAMA Network Open.*

adult neuroblastoma of the skin	C0007129
anaplastic carcinoma of the skin	C0007129
apudoma of skin	C0007129
carcinoma cell merkels	C0007129
carcinoma neuroendocrine skin	C0007129
cell merkel tumors	C0007129
cutaneous apudoma	C0007129
cutaneous neuroendocrine carcinoma	C0007129
cutaneous neuroendocrine tumor	C0007129
endocrine carcinoma of the skin	C0007129
mcc	C0007129
merkel cell cancer	C0007129
merkel cell cancer of the skin	C0007129
merkel cell carcinoma	C0007129
merkel cell carcinoma nos	C0007129
merkel cell skin cancer	C0007129
merkel cell tumor	C0007129
merkel cell tumour	C0007129
merkle tumors	C0007129
neuroendocrine carcinoma of skin	C0007129
neuroendocrine carcinoma of the skin	C0007129
neuroendocrine skin carcinoma	C0007129
neuroendocrine tumor apudoma of skin	C0007129
neuroendocrine tumor of the skin	C0007129
primary cutaneous neuroendocrine carcinoma	C0007129
primary small cell carcinoma of the skin	C0007129
primary undifferentiated carcinoma of the skin	C0007129
small cell neuroepithelial tumor of the skin	C0007129
trabecular cancer	C0007129
trabecular carcinoma of the skin	C0007129
trabecular cell carcinoma of skin	C0007129
trabecular skin carcinoma	C0007129
adenocarcinoma cells renal	C0007134
adenocarcinoma kidneys	C0007134
adenocarcinoma of kidney	C0007134
adenocarcinoma of kidneys	C0007134
adenocarcinoma of the kidney	C0007134
adenocarcinoma renal	C0007134
cancer cell renal	C0007134
cancer cells renal	C0007134
carcinoma cell renal	C0007134
carcinoma cells renal	C0007134
carcinoma kidney	C0007134
carcinoma of kidney	C0007134
carcinoma renal	C0007134
carcinomas renal	C0007134
cell renal cancer	C0007134
grawitz tumor	C0007134
grawitz tumour	C0007134
hypernephroid carcinomas	C0007134
hypernephroma	C0007134
kidney adenocarcinoma	C0007134
kidney carcinoma	C0007134
nephroid carcinoma	C0007134
nephroid carcinomas	C0007134
of kidney carcinoma	C0007134
rcc	C0007134

rccs	C0007134
renal adenocarcinoma	C0007134
renal adenocarcinomas	C0007134
renal carcinoma	C0007134
renal cell adenocarcinoma	C0007134
renal cell adenocarcinomas	C0007134
renal cell cancer	C0007134
renal cell cancers	C0007134
renal cell carcinoma	C0007134
renal cell carcinomas	C0007134
cancer of cervix	C0007847
cancer of the uterine cervix	C0007847
cervical cancer	C0007847
cervical neoplasm malignant cervix uteri	C0007847
mal neo cervix uteri nos	C0007847
malignant cervical neoplasm	C0007847
malignant cervical tumor	C0007847
malignant cervix neoplasm	C0007847
malignant cervix tumor	C0007847
malignant cervix uteri neoplasm	C0007847
malignant cervix uteri tumor	C0007847
malignant neoplasm cervix	C0007847
malignant neoplasm of cervix	C0007847
malignant neoplasm of cervix uteri	C0007847
malignant neoplasm of cervix uteri nos	C0007847
malignant neoplasm of the cervix	C0007847
malignant neoplasm of the cervix uteri	C0007847
malignant neoplasm of the uterine cervix	C0007847
malignant neoplasm of uterine cervix	C0007847
malignant neosplasm of the cervix	C0007847
malignant tumor of cervix	C0007847
malignant tumor of cervix uteri	C0007847
malignant tumor of the cervix	C0007847
malignant tumor of the cervix uteri	C0007847
malignant tumor of the uterine cervix	C0007847
malignant tumor of uterine cervix	C0007847
malignant tumour of cervix	C0007847
malignant uterine cervix neoplasm	C0007847
malignant uterine cervix tumor	C0007847
cancer of large bowel	C0009402
cancer of large intestine	C0009402
cancer of the large bowel	C0009402
cancer of the large intestine	C0009402
carcinoma colorectal	C0009402
carcinoma of large bowel	C0009402
carcinoma of large intestine	C0009402
carcinoma of the large bowel	C0009402
carcinoma of the large intestine	C0009402
colorectal cancer	C0009402
colorectal carcinoma	C0009402
colorectal carcinomas	C0009402
crc	C0009402
large bowel cancer	C0009402
large bowel carcinoma	C0009402
large intestine cancer	C0009402
large intestine carcinoma	C0009402
esophageal cancer	C0014859
esophageal mass	C0014859
esophageal masses	C0014859

© 2021 Yuan Q et al. *JAMA Network Open.*

esophageal neopl	C0014859
esophageal neoplasm	C0014859
esophageal neoplasms	C0014859
esophageal tumor	C0014859
esophageal tumor or cancer	C0014859
esophageal tumors	C0014859
esophagus neopl	C0014859
esophagus neoplasm	C0014859
esophagus neoplasms	C0014859
esophagus tumor	C0014859
esophagus tumors	C0014859
neopl esophageal	C0014859
neoplasm of esophagus	C0014859
neoplasm of oesophagus	C0014859
neoplasm of the esophagus	C0014859
tumor of esophagus	C0014859
tumor of the esophagus	C0014859
tumour of oesophagus	C0014859
cell granulomatosis langerhans	C0019621
cell histiocytosis langerhan	C0019621
cell histiocytosis langerhans	C0019621
cells histiocytosis langerhans	C0019621
christian disease hand schuller	C0019621
christian diseases hand schuller	C0019621
chronic differentiated progressive histiocytosis	C0019621
chronic disseminated histiocytosis x	C0019621
chronic histiocytosis x	C0019621
chronic idiopathic xanthomatosis	C0019621
classic multifocal langerhans cell histiocytosis	C0019621
differentiated progressive histiocytosis	C0019621
disease hand schuller christian	C0019621
generalised histiocytosis of bones	C0019621
generalized histiocytoses	C0019621
generalized histiocytosis	C0019621
generalized histiocytosis of bones	C0019621
hand christian schuller disease	C0019621
hand schueller christian disease	C0019621
hand schueller christian syndrome	C0019621
hand schuller christian disease	C0019621
hand schÃƒÂ¼ller christian disease	C0019621
hand schÃƒÂ¼ller christian syndrome	C0019621
histiocytosis x	C0019621
histiocytosis x ii	C0019621
histiocytosis x syndrome	C0019621
langerhan's cell histiocytosis	C0019621
langerhans cell disease	C0019621
langerhans cell granulomatoses	C0019621
langerhans cell granulomatosis	C0019621
langerhans cell histiocytoses	C0019621
langerhans cell histiocytosis	C0019621
langerhans histiocytic syndrome	C0019621
langerhans' cell histiocytosis	C0019621
lch	C0019621
letterer siwe dis	C0019621
letterer siwe disease	C0019621
multifocal and unisystemic langerhans cell histiocytosis	C0019621

multifocal eosinophilic granuloma	C0019621
multifocal unisystem langerhans cell histiocytosis	C0019621
non lipid reticuloendotheliosis	C0019621
schueller christian dis	C0019621
schueller christian disease	C0019621
schuller christian syndrome	C0019621
systemic aleukemic reticuloendothelioses	C0019621
systemic aleukemic reticuloendotheliosis	C0019621
systemic reticuloendothelial granuloma	C0019621
type 2 histiocytoses	C0019621
type 2 histiocytosis	C0019621
hodgins	C0019829
hodgkin	C0019829
hodgkin dis	C0019829
hodgkin disease	C0019829
hodgkin disease granuloma	C0019829
hodgkin disease paragranuloma	C0019829
hodgkin disease sarcoma	C0019829
hodgkin granuloma	C0019829
hodgkin lymphoma	C0019829
hodgkin lymphomas	C0019829
hodgkin sarcoma	C0019829
hodgkin's disease	C0019829
hodgkin's disease nos	C0019829
hodgkin's disease/lymphoma	C0019829
hodgkin's granuloma	C0019829
hodgkin's granuloma nos	C0019829
hodgkin's granuloma of unspecified site	C0019829
hodgkin's lymphoma	C0019829
hodgkin's lymphoma disease	C0019829
hodgkin's lymphoma nos	C0019829
hodgkin's lymphomas	C0019829
hodgkin's paragranuloma	C0019829
hodgkin's paragranuloma nos	C0019829
hodgkin's paragranuloma of unspecified site	C0019829
hodgkin's sarcoma	C0019829
hodgkin's sarcoma nos	C0019829
hodgkin's sarcoma of unspecified site	C0019829
hodgkin`s disease	C0019829
hodgkins dis	C0019829
hodgkins disease	C0019829
hodgkins disease systemic	C0019829
hodgkins diseases	C0019829
hodgkins granuloma	C0019829
hodgkins lymphoma	C0019829
hodgkins lymphomas	C0019829
hodgkins sarcoma	C0019829
lymphogranulomatosis	C0019829
malignant granuloma	C0019829
malignant granulomas	C0019829
malignant hodgkin's lymphoma	C0019829
malignant lymphogranuloma	C0019829
malignant lymphogranulomas	C0019829
malignant lymphogranulomatosis	C0019829
all types of leukemia	C0023418
leucocythaemia	C0023418
leucocythaemias	C0023418

© 2021 Yuan Q et al. *JAMA Network Open.*

leucocythemia	C0023418
leucocythemias	C0023418
leukaemia	C0023418
leukaemia morphology	C0023418
leukaemia nos	C0023418
leukaemia of unspecified cell type	C0023418
leukaemias	C0023418
leukemia	C0023418
leukemia morphology	C0023418
leukemia nos	C0023418
leukemia of unspecified cell type	C0023418
leukemia type	C0023418
leukemia types	C0023418
leukemias	C0023418
leukemias types	C0023418
type leukemia	C0023418
types leukemia	C0023418
unspecified leukemia	C0023418
unspecified leukemias	C0023418
b cell chronic lymphocytic leukemia	C0023434
b cell cll	C0023434
b cell leukemia	C0023434
b cell lymphocytic leukemia	C0023434
b chronic lymphocytic leukemia	C0023434
bcll	C0023434
chronic leukemia lymphocytic	C0023434
chronic lymphatic leukaemia	C0023434
chronic lymphatic leukemia	C0023434
chronic lymphoblastic leukemia	C0023434
chronic lymphoblastic leukemias	C0023434
chronic lymphocytic leukaemia	C0023434
chronic lymphocytic leukemia	C0023434
chronic lymphocytic leukemias	C0023434
chronic lymphogenous leukemia	C0023434
chronic lymphoid leukaemia	C0023434
chronic lymphoid leukemia	C0023434
cll	C0023434
cll chronic lymphocytic leukemia	C0023434
cll lymphoplasmacytoid lymphoma	C0023434
cll lymphoplasmacytoid lymphomas	C0023434
clls	C0023434
diffuse well differ lymphocytic lymphoma	C0023434
diffuse well differentiated lymphocytic lymphoma	C0023434
leukemia b cell	C0023434
leukemia chronic lymphocytic	C0023434
leukemia lymphocytic chronic	C0023434
lymphocytic leukemia chronic	C0023434
lymphocytic leukemia chronic b	C0023434
lymphocytic lymphoma	C0023434
lymphocytic lymphoma diffuse well differ	C0023434
lymphocytic lymphoma well differ	C0023434
lymphocytic lymphomas	C0023434
lymphoma lymphocytic diffuse well differ	C0023434
lymphoma lymphocytic well differ	C0023434
lymphoma small	C0023434
small cell lymphoma	C0023434
small lymphocytic lymphoma	C0023434
small lymphocytic lymphomas	C0023434

small lymphoma	C0023434
cell hairy leukemia	C0023443
cells hairy leukemia	C0023443
hairy cell leukaemia	C0023443
hairy cell leukemia	C0023443
hairy cell leukemia nos	C0023443
hairy cell leukemias	C0023443
hairy t cell leukemia	C0023443
hcl	C0023443
leukaemia hairy cell	C0023443
leukaemic reticuloendotheliosis	C0023443
leukaemic reticuloendotheliosis nos	C0023443
leukaemic reticuloendotheliosis of unspecified sites	C0023443
leukemia hairy cell	C0023443
leukemic reticuloendothelioses	C0023443
leukemic reticuloendotheliosis	C0023443
leukemic reticuloendotheliosis nos	C0023443
leukemic reticuloendotheliosis of unspecified sites	C0023443
acute leukaemia lymphoblastic	C0023449
acute leukemia lymphoblastic	C0023449
acute leukemia lymphocytic	C0023449
acute leukemia lymphoid	C0023449
acute lymphatic leukaemia	C0023449
acute lymphatic leukemia	C0023449
acute lymphoblastic leukaemia	C0023449
acute lymphoblastic leukaemia nos	C0023449
acute lymphoblastic leukemia	C0023449
acute lymphoblastic leukemia nos	C0023449
acute lymphoblastic leukemias	C0023449
acute lymphocytic leukaemia	C0023449
acute lymphocytic leukemia	C0023449
acute lymphocytic leukemias	C0023449
acute lymphogenous leukemia	C0023449
acute lymphoid leukaemia	C0023449
acute lymphoid leukemia	C0023449
leukemia acute lymphoblastic	C0023449
leukemia lymphoblastic	C0023449
leukemia lymphoblastic acute	C0023449
lymphoblastic leukaemia	C0023449
lymphoblastic leukemia	C0023449
lymphocytic leukemia acute	C0023449
precursor cell lymphoblastic leukemia	C0023449
precursor lymphoblasic leukemia	C0023449
precursor lymphoblastic leukemia	C0023449
acute granulocytic leukaemia	C0023467
acute granulocytic leukemia	C0023467
acute leukemias non lymphoblastic	C0023467
acute myeloblastic leukaemia	C0023467
acute myeloblastic leukemia	C0023467
acute myeloblastic leukemia nos	C0023467
acute myeloblastic leukemias	C0023467
acute myelocytic leukaemia	C0023467
acute myelocytic leukemia	C0023467
acute myelocytic leukemias	C0023467
acute myelogenous leukaemia	C0023467
acute myelogenous leukemia	C0023467
acute myelogenous leukemias	C0023467

© 2021 Yuan Q et al. *JAMA Network Open.*

acute myeloid leukaemia	C0023467
acute myeloid leukemia	C0023467
acute myeloid leukemia nos	C0023467
acute myeloid leukemia not otherwise categorized	C0023467
acute myeloid leukemia not otherwise specified	C0023467
acute myeloid leukemias	C0023467
acute nonlymphoblastic leukemia	C0023467
acute nonlymphoblastic leukemias	C0023467
acute nonlymphocytic leukemia	C0023467
acute nonlymphocytic leukemias	C0023467
aml	C0023467
anll	C0023467
leukemia acute myeloblastic	C0023467
leukemia myeloblastic acute	C0023467
granulocytic leukaemia	C0023470
granulocytic leukemia	C0023470
granulocytic leukemias	C0023470
leukaemia granulocytic	C0023470
leukaemia myelogenous	C0023470
leukemia granulocytic	C0023470
leukemia myelocytic	C0023470
leukemia myelogenous	C0023470
leukemia myeloid	C0023470
myelocytic leukaemia	C0023470
myelocytic leukemia	C0023470
myelocytic leukemias	C0023470
myelogenous leukaemia	C0023470
myelogenous leukemia	C0023470
myelogenous leukemias	C0023470
myeloid granulocytic leukemia	C0023470
myeloid leukaemia	C0023470
myeloid leukaemia nos	C0023470
myeloid leukemia	C0023470
myeloid leukemia nos	C0023470
myeloid leukemias	C0023470
myelosis	C0023470
unspecified myeloid leukemia	C0023470
cgl	C0023473
chronic granulocytic leukaemia	C0023473
chronic granulocytic leukemia	C0023473
chronic granulocytic leukemias	C0023473
chronic myelocytic leukaemia	C0023473
chronic myelocytic leukemia	C0023473
chronic myelocytic leukemias	C0023473
chronic myelogenous leukaemia	C0023473
chronic myelogenous leukemia	C0023473
chronic myelogenous leukemias	C0023473
chronic myeloid leukaemia	C0023473
chronic myeloid leukaemia nos	C0023473
chronic myeloid leukemia	C0023473
chronic myeloid leukemia nos	C0023473
chronic myeloid leukemias	C0023473
chronic myelosis	C0023473
leukemia chronic myelocytic	C0023473
leukemia myelocytic chronic	C0023473
leukemia phila pos	C0023473
myelosis	C0023473

germinoblastic sarcoma	C0024299
germinoblastic sarcomas	C0024299
germinoblastoma	C0024299
germinoblastomas	C0024299
lymphoma	C0024299
lymphoma malignant	C0024299
lymphoma morphology	C0024299
lymphoma nos	C0024299
lymphomas	C0024299
lymphomas malignant	C0024299
lymphomatous	C0024299
lymphosarcoma	C0024299
malignant lymphoma	C0024299
malignant lymphoma nos	C0024299
malignant lymphoma nos of unspecified site	C0024299
malignant lymphomas	C0024299
reticulolymphosarcoma	C0024299
reticulolymphosarcomas	C0024299
diffuse small cleaved cell lymphoma	C0024305
diffuse small cleaved lymphoma	C0024305
lymphoma small cleaved diffuse	C0024305
malignant lymphoma non hodgkins type	C0024305
nhl	C0024305
non hodgkin lymphoma	C0024305
non hodgkin's lymphoma	C0024305
non hodgkins lymphoma	C0024305
nonhodgkin lymphoma	C0024305
nonhodgkin's lymphoma	C0024305
nonhodgkins lymphoma	C0024305
small cleaved lymphoma diffuse	C0024305
ca stomach nos	C0024623
cancer of stomach	C0024623
cancer of the stomach	C0024623
gastric cancer	C0024623
gastric cancer nos	C0024623
gastric cancers	C0024623
malig neopl stomach nos	C0024623
malignant gastric neoplasm	C0024623
malignant gastric tumor	C0024623
malignant neoplasm of stomach	C0024623
malignant neoplasm of stomach nos	C0024623
malignant neoplasm of the stomach	C0024623
malignant neoplasm stomach	C0024623
malignant neosplasm of the stomach	C0024623
malignant tumor of stomach	C0024623
malignant tumor of the stomach	C0024623
malignant tumour of stomach	C0024623
stomach ca	C0024623
stomach cancer	C0024623
stomach cancers	C0024623
cutaneous melanoma	C0025202
malignant melanoma	C0025202
malignant melanoma nos	C0025202
malignant melanomas	C0025202
malignant neoplasm melanoma	C0025202
melanocarcinoma	C0025202
melanoma	C0025202
melanoma malignant	C0025202

© 2021 Yuan Q et al. *JAMA Network Open.*

melanoma syndrome	C0025202
melanomas	C0025202
melanosarcoma	C0025202
nevocarcinoma	C0025202
kahler	C0026764
kahler disease	C0026764
kahler's disease	C0026764
multiple myeloma	C0026764
multiple myeloma / plasma cell neoplasm	C0026764
multiple myeloma and other plasma cell neoplasms	C0026764
multiple myeloma myelomatosis	C0026764
multiple myeloma nos	C0026764
multiple myeloma/plasma cell myeloma	C0026764
multiple myelomas	C0026764
multiple myelomatosis	C0026764
myeloma	C0026764
myeloma multiple	C0026764
myelomas	C0026764
myelomatoses	C0026764
myelomatosis	C0026764
myelomatosis multiple	C0026764
plasma cell myeloma	C0026764
plasma cell myelomas	C0026764
plasma cell neoplasm	C0026764
plasma cell neoplasms	C0026764
plasmacytic myeloma	C0026764
ctcl/ mycosis fungoides	C0026948
mycosis fungoides	C0026948
mycosis fungoides lymphoma	C0026948
mycosis fungoides nos	C0026948
mycosis fungoides of unspecified site	C0026948
chronic myeloproliferative disease	C0027022
disease myeloproliferative	C0027022
diseases myeloproliferative	C0027022
disorder myeloproliferative	C0027022
disorders myeloproliferative	C0027022
myeloproliferative dis	C0027022
myeloproliferative disease	C0027022
myeloproliferative disorder	C0027022
myeloproliferative disorders	C0027022
myeloproliferative neoplasm	C0027022
myeloproliferative neoplasms	C0027022
proliferation of myeloid cells	C0027022
endocrine adenomatosis	C0027662
endocrine multiple neoplasia	C0027662
endocrine neopl multiple	C0027662
familial endocrine adenomatoses	C0027662
familial endocrine adenomatosis	C0027662
familial polyendocrine adenomatosis	C0027662
mea	C0027662
mea syndrome	C0027662
meas	C0027662
men syndrome	C0027662
men syndromes	C0027662
multiple endocrine adenoma	C0027662
multiple endocrine adenomas	C0027662
multiple endocrine adenomatoses	C0027662
multiple endocrine adenomatosis	C0027662

multiple endocrine adenomatosis nos	C0027662
multiple endocrine adenomatosis syndrome	C0027662
multiple endocrine adenopathies	C0027662
multiple endocrine adenopathy	C0027662
multiple endocrine neopl	C0027662
multiple endocrine neopl syndromes	C0027662
multiple endocrine neoplasia	C0027662
multiple endocrine neoplasia syndrome	C0027662
multiple endocrine neoplasia syndromes	C0027662
multiple endocrine neoplasias	C0027662
multiple endocrine neoplasm	C0027662
multiple endocrine neoplasms	C0027662
multiple endocrine tumasia	C0027662
neopl multiple endocrine	C0027662
neuroblastoma	C0027819
neuroblastoma nos	C0027819
neuroblastomas	C0027819
bone sarcoma	C0029463
bone sarcomas	C0029463
osrc	C0029463
osteochondrosarcoma	C0029463
osteogenic sarcoma	C0029463
osteogenic sarcomas	C0029463
osteoid sarcoma	C0029463
osteosarcoma	C0029463
osteosarcoma nos	C0029463
osteosarcoma tumor	C0029463
osteosarcoma tumors	C0029463
osteosarcomas	C0029463
sarcoma osteogenic	C0029463
ca ovarian	C0029925
cancer of ovary	C0029925
cancer of the ovary	C0029925
cancer ovarian	C0029925
cancer ovaries	C0029925
cancer ovary	C0029925
cancers ovarian	C0029925
carcinoma of ovary	C0029925
carcinoma ovarian	C0029925
carcinoma ovaries	C0029925
carcinoma ovary	C0029925
malignant ovaries tumor	C0029925
ovarian ca	C0029925
ovarian cancer	C0029925
ovarian cancers	C0029925
ovarian carcinoma	C0029925
ovarian carcinomas	C0029925
ovary cancer	C0029925
ovary carcinoma	C0029925
carotid body tumors	C0030421
chemodectomas	C0030421
gangliocytic paraganglioma	C0030421
gangliocytic paragangliomas	C0030421
neoplasm of paraganglia	C0030421
neoplasm of paraganglion	C0030421
neoplasm of the paraganglion	C0030421
paraganglia neoplasm	C0030421
paraganglioma	C0030421

© 2021 Yuan Q et al. *JAMA Network Open.*

paraganglioma gangliocytic	C0030421
paraganglioma nos	C0030421
paragangliomas	C0030421
paragangliomas 1	C0030421
paragangliomata	C0030421
paraganglion neoplasm	C0030421
paraganglion tumor	C0030421
paraganglionic neoplasm	C0030421
paraganglionic tumor	C0030421
pgl	C0030421
pgl1	C0030421
tumor of paraganglion	C0030421
tumor of the paraganglion	C0030421
chromaffin paraganglioma	C0031511
chromaffin tumor	C0031511
chromaffin tumors	C0031511
chromaffin tumour	C0031511
chromaffinoma	C0031511
phaeochromocytoma	C0031511
phaeochromocytoma nos	C0031511
pheochromocytoma	C0031511
pheochromocytoma nos	C0031511
pheochromocytoma syndrome	C0031511
pheochromocytomas	C0031511
disorders retinoblastoma	C0035335
malignant retinoblastoma of eye	C0035335
neuroblastoma of retina	C0035335
neuroblastoma of the retina	C0035335
retinal glioblastoma	C0035335
retinal glioblastomas	C0035335
retinal glioma	C0035335
retinal gliomas	C0035335
retinal neuroblastoma	C0035335
retinal neuroblastomas	C0035335
retinoblastoma	C0035335
retinoblastoma eye cancer	C0035335
retinoblastoma eye cancers	C0035335
retinoblastoma nos	C0035335
retinoblastomas	C0035335
malignant neoplasm myosarcoma rhabdomyosarcoma	C0035412
rhabdomyoblastoma	C0035412
rhabdomyosarcoma	C0035412
rhabdomyosarcoma nos	C0035412
rhabdomyosarcomas	C0035412
rhabdosarcoma	C0035412
endotheliosarcoma	C0036220
hhv 8	C0036220
hhv8	C0036220
kaposi sarcoma	C0036220
kaposi's sarcoma	C0036220
kaposi's sarcoma nos	C0036220
kaposi's sarcoma of unspecified site	C0036220
kaposis sarcoma	C0036220
malignant neoplasm sarcoma kaposi's	C0036220
multiple haemorrhagic sarcoma	C0036220
multiple hemorrhagic sarcoma	C0036220
multiple idiopathic pigmented hemangiosarcoma	C0036220

gland neoplasms thyroid	C0040136
gland thyroid tumors	C0040136
gland thyroid tumours	C0040136
neopl thyroid	C0040136
neoplasia of the thyroid gland	C0040136
neoplasm located in the thyroid gland	C0040136
neoplasm of the thyroid	C0040136
neoplasm of the thyroid gland	C0040136
neoplasm of thyroid	C0040136
neoplasm of thyroid gland	C0040136
neoplasm thyroid	C0040136
neoplasms thyroid	C0040136
thryoid neoplasia	C0040136
thyroid cancer	C0040136
thyroid gland neoplasm	C0040136
thyroid gland tumor	C0040136
thyroid neopl	C0040136
thyroid neoplasm	C0040136
thyroid neoplasms	C0040136
thyroid tumor	C0040136
thyroid tumors	C0040136
thyroid tumour	C0040136
thyroid tumours	C0040136
tumor of the thyroid	C0040136
tumor of the thyroid gland	C0040136
tumor of thyroid	C0040136
tumor of thyroid gland	C0040136
tumor thyroid	C0040136
tumor thyroid gland	C0040136
tumour of thyroid gland	C0040136
cancer of the vagina	C0042237
cancer of vagina	C0042237
cancer vagina	C0042237
cancer vaginal	C0042237
malign neopl vagina	C0042237
malignant neoplasm of the vagina	C0042237
malignant neoplasm of vagina	C0042237
malignant neoplasm of vagina nos	C0042237
malignant tumor of the vagina	C0042237
malignant tumor of vagina	C0042237
malignant tumour of vagina	C0042237
malignant vagina neoplasm	C0042237
malignant vagina tumor	C0042237
malignant vaginal neoplasm	C0042237
malignant vaginal tumor	C0042237
vagina cancer	C0042237
vagina cancers	C0042237
vaginal cancer	C0042237
vaginal cancers	C0042237
vaginal neoplasm malignant	C0042237
cell lymphoma t	C0079772
cell lymphomas t	C0079772
cells lymphoma t	C0079772
lymphoma t cell	C0079772
t cell lymphoma	C0079772
cells cutaneous lymphomas t	C0079773
ctcl	C0079773
cutaneous t cell lymphoma	C0079773
pctcl	C0079773

t cell cutaneous lymphoma	C0079773
carcinoma lip	C0149637
carcinoma of lip	C0149637
carcinoma of the lip	C0149637
carcinomas lip	C0149637
lip cancer	C0149637
lip carcinoma	C0149637
lip neoplasm malignant carcinoma	C0149637
cancer of esophagus	C0152018
cancer of oesophagus	C0152018
cancer of the esophagus	C0152018
carcinoma esophageal	C0152018
carcinoma esophageal cancer	C0152018
carcinoma esophagus	C0152018
carcinoma oesophagus	C0152018
carcinoma of esophagus	C0152018
carcinoma of oesophagus	C0152018
carcinoma of the esophagus	C0152018
carcinoma of the oesophagus	C0152018
esophageal cancer	C0152018
esophageal carcinoma	C0152018
esophagus carcinoma	C0152018
oesophageal carcinoma	C0152018
cancer lip	C0153340
cancer lips	C0153340
cancer of lip	C0153340
cancer of the lip	C0153340
lip cancer	C0153340
lip cancers	C0153340
lip neoplasm malignant	C0153340
malignant lip neoplasm	C0153340
malignant lip tumor	C0153340
malignant neoplasm of lip	C0153340
malignant neoplasm of the lip	C0153340
malignant tumor of lip	C0153340
malignant tumor of the lip	C0153340
malignant tumour of lip	C0153340
of lip cancer	C0153340
cancer of mouth	C0153381
cancer of oral cavity	C0153381
cancer of the mouth	C0153381
malig neoplasm mouth nos	C0153381
malignant mouth neoplasm	C0153381
malignant mouth tumor	C0153381
malignant neoplasm of mouth	C0153381
malignant neoplasm of mouth nos	C0153381
malignant neoplasm of oral cavity	C0153381
malignant neoplasm of oral cavity nos	C0153381
malignant neoplasm of the mouth	C0153381
malignant oral cavity neoplasm	C0153381
malignant oral cavity tumor	C0153381
malignant oral neoplasm	C0153381
malignant tumor of mouth	C0153381
malignant tumor of oral cavity	C0153381
malignant tumor of the mouth	C0153381
malignant tumour of mouth	C0153381
malignant tumour of oral cavity	C0153381
mouth cancer	C0153381
mouth cancers	C0153381

oral cancer	C0153381
oral cancers	C0153381
oral cavity cancer	C0153381
cancer of oropharynx	C0153382
cancer of the oropharynx	C0153382
cancer oropharyngeal	C0153382
cancer oropharynx	C0153382
malig neo oropharynx nos	C0153382
malignant neoplasm of oropharynx	C0153382
malignant neoplasm of oropharynx nos	C0153382
malignant neoplasm of the oropharynx	C0153382
malignant oropharyngeal neoplasm	C0153382
malignant oropharyngeal tumor	C0153382
malignant tumor of mesopharynx	C0153382
malignant tumor of oropharynx	C0153382
malignant tumor of the oropharynx	C0153382
malignant tumour of mesopharynx	C0153382
malignant tumour of oropharynx	C0153382
oropharyngeal cancer	C0153382
oropharynx cancer	C0153382
cancer nasopharyngeal	C0153392
cancer nasopharynx	C0153392
mal neo nasopharynx nos	C0153392
malignant nasopharyngeal neoplasm	C0153392
malignant nasopharyngeal tumor	C0153392
malignant neoplasm of nasopharynx	C0153392
malignant neoplasm of nasopharynx nos	C0153392
malignant neoplasm of the nasopharynx	C0153392
malignant tumor of epipharynx	C0153392
malignant tumor of nasopharynx	C0153392
malignant tumor of postnasal space	C0153392
malignant tumor of the nasopharynx	C0153392
malignant tumour of epipharynx	C0153392
malignant tumour of nasopharynx	C0153392
malignant tumour of postnasal space	C0153392
nasopharyngeal cancer	C0153392
nasopharynx cancer	C0153392
cancer hypopharyngeal	C0153398
cancer hypopharynx	C0153398
hypopharyngeal cancer	C0153398
hypopharyngeal cancers	C0153398
hypopharynx cancer	C0153398
mal neo hypopharynx nos	C0153398
malignant hypopharyngeal neoplasm	C0153398
malignant hypopharyngeal tumor	C0153398
malignant neoplasm of hypopharynx	C0153398
malignant neoplasm of hypopharynx nos	C0153398
malignant neoplasm of laryngopharynx	C0153398
malignant neoplasm of the hypopharynx	C0153398
malignant tumor of hypopharynx	C0153398
malignant tumor of laryngopharynx	C0153398
malignant tumor of the hypopharynx	C0153398
malignant tumour of hypopharynx	C0153398
malignant tumour of laryngopharynx	C0153398
cancer of pharynx	C0153405
cancer of the pharynx	C0153405
cancer pharyngeal	C0153405
cancer pharynx	C0153405
mal neo pharynx nos	C0153405

© 2021 Yuan Q et al. *JAMA Network Open.*

malignant neoplasm of pharynx	C0153405
malignant neoplasm of pharynx unspecified	C0153405
malignant pharyngeal neoplasm	C0153405
malignant pharyngeal tumor	C0153405
malignant pharynx neoplasm	C0153405
malignant pharynx tumor	C0153405
malignant tumor of pharynx	C0153405
malignant tumor of the pharynx	C0153405
malignant tumour of pharynx	C0153405
pharnyx cancer	C0153405
pharnyx cancers	C0153405
pharyngeal cancer	C0153405
pharyngeal cancers	C0153405
pharynx cancer	C0153405
pharynx cancers	C0153405
pharynx neoplasm malignant	C0153405
bowel cancer small	C0153425
cancer intestine small	C0153425
cancer intestines small	C0153425
cancer small intestine	C0153425
cancers intestines small	C0153425
mal neo small bowel nos	C0153425
malignant neoplasm of small bowel	C0153425
malignant neoplasm of small intestine	C0153425
malignant neoplasm of small intestine nos	C0153425
malignant neoplasm of the small bowel	C0153425
malignant neoplasm of the small intestine	C0153425
malignant neosplasm of the small intestine	C0153425
malignant small bowel neoplasm	C0153425
malignant small bowel tumor	C0153425
malignant small intestinal neoplasm	C0153425
malignant small intestine neoplasm	C0153425
malignant small intestine tumor	C0153425
malignant tumor of small bowel	C0153425
malignant tumor of small intestine	C0153425
malignant tumor of the small bowel	C0153425
malignant tumor of the small intestine	C0153425
malignant tumour of small bowel	C0153425
malignant tumour of small intestine	C0153425
small bowel cancer	C0153425
small intestine ca	C0153425
small intestine cancer	C0153425
anal cancer	C0153446
anal cancers	C0153446
anus cancer	C0153446
cancer anus	C0153446
cancer of the anus	C0153446
malignant anal neoplasm	C0153446
malignant anal tumor	C0153446
malignant neo anus nos	C0153446
malignant neoplasm of anus	C0153446
malignant neoplasm of anus unspecified	C0153446
malignant neoplasm of the anus	C0153446
malignant neosplasm of the anus	C0153446
malignant tumor of anal margin	C0153446
malignant tumor of anus	C0153446
malignant tumor of the anus	C0153446
malignant tumour of anal margin	C0153446

malignant tumour of anus	C0153446
cancer gallbladder	C0153452
cancer of gallbladder	C0153452
cancer of the gallbladder	C0153452
gall bladder cancer	C0153452
gall bladder cancers	C0153452
gallbladder ca	C0153452
gallbladder cancer	C0153452
gallbladder cancers	C0153452
gallbladder malignant neoplasm	C0153452
gallbladder tumor or cancer	C0153452
malig neo gallbladder	C0153452
malignant gallbladder neoplasm	C0153452
malignant gallbladder tumor	C0153452
malignant neoplasm of gallbladder	C0153452
malignant neoplasm of the gallbladder	C0153452
malignant tumor of gallbladder	C0153452
malignant tumor of the gallbladder	C0153452
malignant tumour of gallbladder	C0153452
of gallbladder cancer	C0153452
ca uterus nos	C0153567
cancer of the uterus	C0153567
cancer of uterus	C0153567
malig neopl uterus nos	C0153567
malignant neoplasm of the uterus	C0153567
malignant neoplasm of uterus	C0153567
malignant neosplasm of the uterus	C0153567
malignant tumor of the uterus	C0153567
malignant tumor of uterus	C0153567
malignant tumour of uterus	C0153567
malignant uterine neoplasm	C0153567
malignant uterine tumor	C0153567
uterine ca nos	C0153567
uterine cancer	C0153567
uterine cancers	C0153567
uterus cancer	C0153567
uterus cancers	C0153567
cancer fallopian tube	C0153579
cancer fallopian tubes	C0153579
cancer of the fallopian tube	C0153579
fallopian tube cancer	C0153579
fallopian tube malignant neoplasm	C0153579
fallopian tube malignant tumor	C0153579
fallopian tubes cancer	C0153579
mal neo fallopian tube	C0153579
malignant fallopian tube neoplasm	C0153579
malignant fallopian tube tumor	C0153579
malignant neoplasm of fallopian tube	C0153579
malignant neoplasm of oviduct	C0153579
malignant neoplasm of the fallopian tube	C0153579
malignant neoplasm of uterine tube	C0153579
malignant tumor of fallopian tube	C0153579
malignant tumor of the fallopian tube	C0153579
malignant tumour of fallopian tube	C0153579
cancer of testis	C0153594
cancer of the testes	C0153594
cancer of the testis	C0153594
cancer testicle	C0153594
cancer testicles	C0153594

© 2021 Yuan Q et al. *JAMA Network Open.*

cancer testicular	C0153594
cancer testis	C0153594
malignant neoplasm of testis	C0153594
malignant neoplasm of testis nos	C0153594
malignant neoplasm of the testis	C0153594
malignant neosplasm of the testis	C0153594
malignant testicular neoplasm	C0153594
malignant testicular tumor	C0153594
malignant tumor of testis	C0153594
malignant tumor of the testis	C0153594
malignant tumour of testis	C0153594
testicle cancer	C0153594
testicular cancer	C0153594
testicular cancers	C0153594
testis cancer	C0153594
testis cancers	C0153594
testis neoplasm malignant	C0153594
ca penis	C0153601
cancer of penis	C0153601
cancer of the penis	C0153601
cancer penile	C0153601
cancer penis	C0153601
malig neo penis nos	C0153601
malignant neoplasm of penis	C0153601
malignant neoplasm of the penis	C0153601
malignant penile neoplasm	C0153601
malignant penile tumor	C0153601
malignant tumor of penis	C0153601
malignant tumor of the penis	C0153601
malignant tumour of penis	C0153601
of penis cancer	C0153601
penile ca	C0153601
penile cancer	C0153601
penile cancers	C0153601
penis cancer	C0153601
penis cancers	C0153601
cancer of parathyroid gland	C0153653
cancer parathyroids	C0153653
malig neo parathyroid	C0153653
malignant neoplasm of parathyroid	C0153653
malignant neoplasm of parathyroid gland	C0153653
malignant neoplasm of the parathyroid	C0153653
malignant neoplasm of the parathyroid gland	C0153653
malignant parathyroid gland neoplasm	C0153653
malignant parathyroid gland tumor	C0153653
malignant parathyroid neoplasm	C0153653
malignant parathyroid tumor	C0153653
malignant tumor of parathyroid	C0153653
malignant tumor of parathyroid gland	C0153653
malignant tumor of the parathyroid	C0153653
malignant tumor of the parathyroid gland	C0153653
malignant tumour of parathyroid gland	C0153653
of parathyroid cancer	C0153653
parathyroid cancer	C0153653
cell germ tumours	C0205851
cells germ tumors	C0205851
germ cell neopl	C0205851
germ cell neoplasm	C0205851
germ cell neoplasms	C0205851
germ cell tumor	C0205851
germ cell tumors	C0205851
germ cell tumour	C0205851
neopl germ cell	C0205851
neoplasm of germ cell	C0205851
neoplasm of the germ cell	C0205851
tumor of germ cell	C0205851
tumor of the germ cell	C0205851
papillomatoses	C0205875
papillomatosis	C0205875
papillomatosis nos	C0205875
malignant thymoma	C0205969
malignant thymoma of thymus	C0205969
thymic carcinoma	C0205969
thymic carcinomas	C0205969
thymoma and thymic carcinoma	C0205969
adenocarcinoma adrenal	C0206686
adenocarcinoma of adrenal gland	C0206686
adrenal adenocarcinoma	C0206686
adrenal carcinoma	C0206686
adrenal carcinomas	C0206686
adrenal cortex adenocarcinoma	C0206686
adrenal cortex cancer	C0206686
adrenal cortex carcinoma	C0206686
adrenal cortical adenocarcinoma	C0206686
adrenal cortical carcinoma	C0206686
adrenal cortical carcinomas	C0206686
adrenal gland carinoma	C0206686
adrenocortical cancer	C0206686
adrenocortical carcinoma	C0206686
adrenocortical carcinoma of adrenal gland	C0206686
adrenocortical carcinomas	C0206686
cancer of the adrenal cortex	C0206686
carcinoma adrenal	C0206686
carcinoma of adrenal cortex	C0206686
carcinoma of adrenal gland	C0206686
carcinoma of the adrenal cortex	C0206686
cortical cell carcinoma	C0206686
bile duct cancer	C0206698
cholangiocarcinoma	C0206698
cholangiocarcinoma of biliary tract	C0206698
cholangiocarcinomas	C0206698
cholangiocellular carcinoma	C0206698
cholangiocellular carcinomas	C0206698
cholangiosarcoma	C0206698
malignant neoplasm of biliary tract cholangiocarcinoma	C0206698
accessory sinus esthesioneuroblastoma	C0206717
aesthesioneuroblastoma	C0206717
aesthesioneuroblastomas	C0206717
aesthesioneuroepithelioma	C0206717
asthesioneuroblastoma	C0206717
esthesioneuroblastoma	C0206717
esthesioneuroblastoma of accessory sinus	C0206717
esthesioneuroblastoma of paranasal sinus	C0206717
esthesioneuroblastoma of the accessory sinus	C0206717

© 2021 Yuan Q et al. *JAMA Network Open.*

esthesioneuroblastoma of the paranasal sinus	C0206717
esthesioneuroblastoma olfactory	C0206717
esthesioneuroblastomas	C0206717
esthesioneuroepithelioma	C0206717
malignant neoplasm neuroblastoma olfactory	C0206717
nasal cavity and paranasal sinus esthesioneuroblastoma	C0206717
olfactory esthesioneuroblastoma	C0206717
olfactory esthesioneuroblastomas	C0206717
olfactory neuroblastoma	C0206717
olfactory neuroblastomas	C0206717
olfactory neuroepithelioma	C0206717
paranasal sinus and nasal cavity esthesioneuroblastoma	C0206717
paranasal sinus esthesioneuroblastoma	C0206717
paranasal sinus nasal cavity esthesioneuroblastoma	C0206717
paranasal sinus olfactory neuroblastoma	C0206717
adult hd	C0220597
adult hodgkin disease	C0220597
adult hodgkin lymphoma	C0220597
adult hodgkin's disease	C0220597
adult hodgkin's lymphoma	C0220597
adult hodgkins disease	C0220597
adult hodgkins lymphoma	C0220597
adults disease hodgkin's	C0220597
hodgkin lymphoma	C0220597
adult nhl	C0220605
childhood rhabdomyosarcoma	C0220611
pediatric rhabdomyosarcoma	C0220611
rhabdomyosarcoma	C0220611
childhood nhl	C0220612
pediatric nhl	C0220612
adult sarcoma of soft tissue	C0220613
adult sarcoma of the soft tissue	C0220613
adult soft tissue sarcoma	C0220613
adult sts	C0220613
soft tissue sarcoma	C0220613
soft tissue sarcoma adult	C0220613
carcinoid gastrointestinal tumors	C0220620
carcinoid tumor of digestive system	C0220620
carcinoid tumor of gastrointestinal system	C0220620
carcinoid tumor of gastrointestinal tract	C0220620
carcinoid tumor of gi system	C0220620
carcinoid tumor of the digestive system	C0220620
carcinoid tumor of the gastrointestinal system	C0220620
carcinoid tumor of the gi system	C0220620
carcinoid tumour of gastrointestinal tract	C0220620
digestive carcinoid tumor	C0220620
digestive system carcinoid tumor	C0220620
digestive system neuroendocrine tumor g1	C0220620
gastrointestinal carcinoid tumor	C0220620
gastrointestinal net g1	C0220620
gastrointestinal neuroendocrine tumor g1	C0220620
gastrointestinal system carcinoid tumor	C0220620
gct	C0220620

gi carcinoid tumor	C0220620
adult liver carcinoma	C0220630
adult primary cancer of liver	C0220630
adult primary cancer of the liver	C0220630
adult primary liver cancer	C0220630
adult primary liver carcinoma	C0220630
liver cancer	C0220630
intraocular melanoma	C0220633
melanoma of the uvea	C0220633
melanoma of uvea	C0220633
uveal melanoma	C0220633
cancer glands salivary	C0220636
cancer of salivary gland	C0220636
cancer of the salivary gland	C0220636
cancer salivary gland	C0220636
glands malignant salivary tumors	C0220636
mal neo salivary nos	C0220636
malignant neoplasm of salivary gland	C0220636
malignant neoplasm of salivary gland duct	C0220636
malignant neoplasm of salivary gland nos	C0220636
malignant neoplasm of the salivary gland	C0220636
malignant salivary gland neoplasm	C0220636
malignant salivary gland tumor	C0220636
malignant tumor of salivary gland	C0220636
malignant tumor of the salivary gland	C0220636
malignant tumour of salivary gland	C0220636
of cancer salivary gland	C0220636
of salivary gland cancer	C0220636
salivary gland cancer	C0220636
salivary gland cancers	C0220636
lip and oral cavity cancer	C0220641
lip and oral cavity carcinoma	C0220641
oral cancer	C0220641
oral carcinoma	C0220641
oral cavity and lip cancer	C0220641
childhood hd	C0220644
childhood hodgkin disease	C0220644
childhood hodgkin lymphoma	C0220644
childhood hodgkin's disease	C0220644
childhood hodgkin's lymphoma	C0220644
childhood hodgkins disease	C0220644
childhood hodgkins lymphoma	C0220644
hodgkin lymphoma	C0220644
pediatric hd	C0220644
pediatric hodgkin's disease	C0220644
pediatric hodgkin's lymphoma	C0220644
childhood sarcoma of soft tissue	C0220645
childhood sarcoma of the soft tissue	C0220645
childhood sarcoma soft tissue	C0220645
childhood soft tissue sarcoma	C0220645
childhood sts	C0220645
pediatric sarcoma of soft tissue	C0220645
pediatric sarcoma of the soft tissue	C0220645
pediatric soft tissue sarcoma	C0220645
pediatric st	C0220645
pediatric sts	C0220645
soft tissue sarcoma	C0220645
bladder carcinoma gall	C0235782
cancer of gallbladder	C0235782

© 2021 Yuan Q et al. *JAMA Network Open.*

cancer of the gallbladder	C0235782
carcinoma gallbladder	C0235782
carcinoma of gallbladder	C0235782
carcinoma of the gall bladder	C0235782
carcinoma of the gallbladder	C0235782
gall bladder carcinoma	C0235782
gallbladder cancer	C0235782
gallbladder carcinoma	C0235782
cancer of pancreas	C0235974
cancer of the pancreas	C0235974
carcinoma of pancreas	C0235974
carcinoma of the pancreas	C0235974
carcinoma pancreas	C0235974
carcinoma pancreatic	C0235974
exocrine cancer	C0235974
exocrine pancreas carcinoma	C0235974
pancreas cancer	C0235974
pancreas carcinoma	C0235974
pancreatic acinar carcinoma	C0235974
pancreatic cancer	C0235974
pancreatic carcinoma	C0235974
cancer of fallopian tube	C0238122
cancer of the fallopian tube	C0238122
carcinoma of fallopian tube	C0238122
carcinoma of the fallopian tube	C0238122
fallopian tube ca	C0238122
fallopian tube cancer	C0238122
fallopian tube cancers	C0238122
fallopian tube carcinoma	C0238122
cancer of small bowel	C0238196
cancer of the small bowel	C0238196
carcinoma intestine small	C0238196
carcinoma of small bowel	C0238196
carcinoma of small intestine	C0238196
carcinoma of the small bowel	C0238196
carcinoma of the small intestine	C0238196
carcinoma small intestine	C0238196
small bowel cancer	C0238196
small bowel carcinoma	C0238196
small intestinal cancer	C0238196
small intestinal carcinoma	C0238196
small intestine cancer	C0238196
small intestine carcinoma	C0238196
gant	C0238198
gants	C0238198
gastrointestinal pacemaker cell tumor	C0238198
gastrointestinal pacemaker cell tumour	C0238198
gastrointestinal stroma tumor	C0238198
gastrointestinal stromal neoplasm	C0238198
gastrointestinal stromal neoplasms	C0238198
gastrointestinal stromal tumor	C0238198
gastrointestinal stromal tumors	C0238198
gastrointestinal stromal tumour	C0238198
gastrointestinal stromal tumours	C0238198
gist	C0238198
gists	C0238198
neoplasm gastrointestinal tract stromal	C0238198
cancer of nasopharynx	C0238301
cancer of the nasopharynx	C0238301

nasopharyngeal cancer	C0238301
nasopharyngeal cancers	C0238301
nasopharynx cancer	C0238301
nasopharynx cancers	C0238301
endocrine pancreas tumor	C0242363
endocrine pancreas tumors	C0242363
endocrine pancreatic tumors	C0242363
endocrine tumor of pancreas	C0242363
endocrine tumour of pancreas	C0242363
island cell tumor	C0242363
island cell tumors	C0242363
islet cell adenoma	C0242363
islet cell neoplasm	C0242363
islet cell tumor	C0242363
islet cell tumors	C0242363
islet cell tumour	C0242363
neoplasm of islets of langerhans	C0242363
nesidioblastoma	C0242363
pancreatic endocrine neoplasm	C0242363
pancreatic endocrine tumor	C0242363
pancreatic endocrine tumour	C0242363
pancreatic neuroendocrine neoplasm	C0242363
tumor of endocrine pancreas	C0242363
tumour of endocrine pancreas	C0242363
cancer of the vagina	C0262659
cancer of vagina	C0262659
carcinoma of the vagina	C0262659
carcinoma of vagina	C0262659
carcinoma vagina	C0262659
carcinoma vaginal	C0262659
vagina cancer	C0262659
vagina carcinoma	C0262659
vaginal cancer	C0262659
vaginal carcinoma	C0262659
cell extragonadal germ tumors	C0262963
extragonadal germ cell neoplasm	C0262963
extragonadal germ cell neoplasms	C0262963
extragonadal germ cell tumor	C0262963
neoplasm of extragonadal germ cell	C0262963
neoplasm of the extragonadal germ cell	C0262963
primary extragonadal germ cell tumor	C0262963
tumor of extragonadal germ cell	C0262963
tumor of the extragonadal germ cell	C0262963
adult burkitt lymphoma	C0278764
adult burkitt's lymphoma	C0278764
adult diffuse small noncleaved cell/burkitt's lymphoma	C0278764
adult snc lymphoma	C0278764
burkitt lymphoma	C0278764
burkitt lymphoma	C0278879
childhood burkitt lymphoma	C0278879
childhood burkitt's lymphoma	C0278879
childhood small noncleaved cell lymphoma	C0278879
childhood snc lymphoma	C0278879
pediatric burkitt's lymphoma	C0278879
pediatric snc lymphoma	C0278879
cancer of liver	C0279000

© 2021 Yuan Q et al. *JAMA Network Open.*

cancer of liver and intrahepatic biliary tract	C0279000
cancer of the liver	C0279000
cancer of the liver and intrahepatic biliary tract	C0279000
hepatic cancer	C0279000
liver and intrahepatic bile duct cancer	C0279000
liver and intrahepatic bile duct carcinoma	C0279000
liver and intrahepatic biliary tract cancer	C0279000
liver and intrahepatic biliary tract carcinoma	C0279000
liver cancer	C0279000
liver/hepatobiliary cancer	C0279000
primary liver carcinoma	C0279000
bone cancer	C0279530
bone cancers	C0279530
bone malignant neoplasm	C0279530
bone malignant tumors	C0279530
cancer of bone	C0279530
cancer of the bone	C0279530
malignant bone neoplasm	C0279530
malignant bone tumor	C0279530
malignant bone tumour	C0279530
malignant neoplasm of bone	C0279530
malignant neoplasm of the bone	C0279530
malignant osseous neoplasm	C0279530
malignant osseous tumor	C0279530
malignant tumor of bone	C0279530
malignant tumor of the bone	C0279530
osseous cancer	C0279530
adult rhabdomyosarcoma	C0279550
rhabdomyosarcoma	C0279550
childhood carcinoma of liver cell	C0279606
childhood carcinoma of the liver cell	C0279606
childhood hepatocellular carcinoma	C0279606
childhood hepatoma	C0279606
childhood liver cell carcinoma	C0279606
hepatocellular cancer	C0279606
pediatric carcinoma of liver cell	C0279606
pediatric carcinoma of the liver cell	C0279606
pediatric hepatocellular carcinoma	C0279606
pediatric hepatoma	C0279606
pediatric liver cell carcinoma	C0279606
adult hepatocellular carcinoma	C0279607
adult hepatoma	C0279607
adult primary carcinoma of liver cell	C0279607
adult primary carcinoma of the liver cell	C0279607
adult primary hepatocellular carcinoma	C0279607
adult primary hepatoma	C0279607
adult primary liver cell carcinoma	C0279607
hepatocellular cancer	C0279607
anal cancer	C0279637
anal cancers	C0279637
anal carcinoma	C0279637
anus cancer	C0279637
anus cancers	C0279637
anus carcinoma	C0279637
anus carcinoma cell squamous	C0279637
anus carcinoma epidermoid	C0279637

ca anus	C0279637
cancer of anus	C0279637
cancer of the anus	C0279637
carcinoma anus	C0279637
carcinoma of anus	C0279637
carcinoma of the anus	C0279637
squamous cell carcinoma anus	C0279637
adult cholangiocarcinoma	C0280725
adult primary cholangiocarcinoma	C0280725
adult primary cholangiocellular carcinoma	C0280725
cholangiocarcinoma	C0280725
cns lymphoma primary	C0280803
malignant neoplasm lymphoma primary central nervous system	C0280803
microglioma	C0280803
pcnsl	C0280803
primary central nervous system lymphoma	C0280803
primary cns lymphoma	C0280803
primary lymphoma cns	C0280803
primary lymphoma of cns	C0280803
ca cervix	C0302592
ca cervix uteri nos	C0302592
cancer of cervix	C0302592
cancer of the cervix	C0302592
cancer of the uterine cervix	C0302592
cancer of uterine cervix	C0302592
carcinoma cervical	C0302592
carcinoma cervix	C0302592
carcinoma cervix uteri	C0302592
carcinoma cervix uterine	C0302592
carcinoma of cervix	C0302592
carcinoma of cervix invasive	C0302592
carcinoma of cervix uteri	C0302592
carcinoma of the cervix	C0302592
carcinoma of the cervix uteri	C0302592
carcinoma of the uterine cervix	C0302592
carcinoma of uterine cervix	C0302592
carcinoma uterine cerix	C0302592
cervical cancer	C0302592
cervical carcinoma	C0302592
cervix cancer	C0302592
cervix carcinoma	C0302592
cervix uteri carcinoma	C0302592
collum carcinoma	C0302592
uterine cervix cancer	C0302592
uterine cervix carcinoma	C0302592
carcinoid	C0334299
carcinoid tumor	C0334299
carcinoid tumor no international classification of diseases for oncology subtype	C0334299
carcinoid tumour	C0334299
typical carcinoid	C0334299
fibrous histiocytoma malignant	C0334463
fibroxanthosarcoma	C0334463
malignant fibrohistiocytic tumor	C0334463
malignant fibrohistiocytic tumors	C0334463
malignant fibrous cytoma	C0334463
malignant fibrous histiocytoma	C0334463

© 2021 Yuan Q et al. *JAMA Network Open.*

malignant fibrous histiocytoma of soft tissue and bone	C0334463
malignant fibrous histiocytoma of the soft tissue and bone	C0334463
malignant fibrous histiocytomas	C0334463
malignant fibroxanthoma	C0334463
mfh	C0334463
unclassified pleomorphic sarcoma	C0334463
undifferentiated high grade pleomorphic sarcoma	C0334463
undifferentiated pleomorphic sarcoma	C0334463
undifferentiated pleomorphic soft tissue sarcoma	C0334463
body of uterus sarcoma	C0338113
corpus uteri sarcoma	C0338113
sarcoma of body of uterus	C0338113
sarcoma of corpus uteri	C0338113
sarcoma of the body of uterus	C0338113
sarcoma of the corpus uteri	C0338113
sarcoma of the uterine body	C0338113
sarcoma of the uterine corpus	C0338113
sarcoma of the uterus	C0338113
sarcoma of uterine body	C0338113
sarcoma of uterine corpus	C0338113
sarcoma of uterus	C0338113
sarcoma uterine	C0338113
sarcoma uterus	C0338113
sarcomas uterine	C0338113
uterine body sarcoma	C0338113
uterine cancer sarcoma	C0338113
uterine corpus sarcoma	C0338113
uterine sarcoma	C0338113
uterine sarcoma cancer	C0338113
uterus sarcoma	C0338113
cancer of liver	C0345904
cancer of the liver	C0345904
hepatic cancer	C0345904
hepatic cancers	C0345904
hepatic neoplasm malignant	C0345904
hepatocellular cancer	C0345904
hepatocellular cancers	C0345904
liver cancer	C0345904
liver cancers	C0345904
liver malignant tumors	C0345904
liver neoplasm malignant	C0345904
liver tumor or cancer	C0345904
malignant neo liver nos	C0345904
malignant neoplasm of liver	C0345904
malignant neoplasm of liver unspecified	C0345904
malignant neosplasm of the liver	C0345904
malignant tumor of liver	C0345904
malignant tumour of liver	C0345904
malignant mesothelial neoplasm	C0345967
malignant mesothelial tumor	C0345967
malignant mesothelioma	C0345967
malignant neoplasm mesothelioma	C0345967
malignant neoplasm of mesothelium	C0345967
malignant neoplasm of the mesothelium	C0345967
malignant tumor of mesothelium	C0345967
malignant tumor of the mesothelium	C0345967
mesom	C0345967
mesothelioma	C0345967
cancer intestine large	C0346629
cancer intestines large	C0346629
cancer of large bowel	C0346629
cancer of large colon	C0346629
cancer of large intestine	C0346629
colon cancer	C0346629
colorectal cancer	C0346629
large bowel cancer	C0346629
large intestine cancer	C0346629
malignant colorectal neoplasm	C0346629
malignant colorectal tumor	C0346629
malignant large bowel neoplasm	C0346629
malignant large bowel tumor	C0346629
malignant large intestine neoplasm	C0346629
malignant large intestine tumor	C0346629
malignant neoplasm of large bowel	C0346629
malignant neoplasm of large intestine	C0346629
malignant neoplasm of large intestine nos	C0346629
malignant neoplasm of the large bowel	C0346629
malignant neoplasm of the large intestine	C0346629
malignant neosplasm of the large bowel	C0346629
malignant neosplasm of the large intestine	C0346629
malignant tumor of large bowel	C0346629
malignant tumor of large intestine	C0346629
malignant tumor of the large bowel	C0346629
malignant tumor of the large intestine	C0346629
malignant tumour of large intestine	C0346629
of large bowel cancer	C0346629
ca pancreas nos	C0346647
cancer of pancreas	C0346647
cancer of the pancreas	C0346647
malig neo pancreas nos	C0346647
malignant neoplasm of pancreas	C0346647
malignant neoplasm of pancreas nos	C0346647
malignant neoplasm of the pancreas	C0346647
malignant neoplasm pancreas	C0346647
malignant neosplasm of the pancreas	C0346647
malignant pancreatic neoplasm	C0346647
malignant tumor of pancreas	C0346647
malignant tumour of pancreas	C0346647
pancreas ca	C0346647
pancreas cancer	C0346647
pancreas cancers	C0346647
pancreas neoplasm malignant	C0346647
pancreatic cancer	C0346647
pancreatic cancers	C0346647
ca vulva	C0375071
cancer of the vulva	C0375071
cancer of vulva	C0375071
cancer vulva	C0375071
cancer vulvar	C0375071
malign neopl vulva nos	C0375071
malignant neoplasm of the vulva	C0375071
malignant neoplasm of vulva	C0375071
malignant neoplasm of vulva unspecified	C0375071
malignant tumor of the vulva	C0375071

© 2021 Yuan Q et al. *JAMA Network Open.*

malignant tumor of vulva	C0375071
malignant tumour of vulva	C0375071
malignant vulva neoplasm	C0375071
malignant vulva tumor	C0375071
malignant vulvar neoplasm	C0375071
malignant vulvar tumor	C0375071
of vulva cancer	C0375071
vulva cancer	C0375071
vulva cancers	C0375071
vulval ca	C0375071
vulval cancer	C0375071
vulvar cancer	C0375071
vulvar cancers	C0375071
vulvar neoplasm malignant	C0375071
cancer of prostate	C0376358
cancer of the prostate	C0376358
malign neopl prostate	C0376358
malignant neoplasm of prostate	C0376358
malignant neoplasm of prostate gland	C0376358
malignant neoplasm of the prostate	C0376358
malignant neoplasm prostate	C0376358
malignant neosplasm of the prostate	C0376358
malignant prostate neoplasm	C0376358
malignant prostate tumor	C0376358
malignant prostatic tumor	C0376358
malignant prostatic tumour	C0376358
malignant tumor of prostate	C0376358
malignant tumor of prostate gland	C0376358
malignant tumor of the prostate	C0376358
malignant tumour of prostate	C0376358
prostate cancer	C0376358
prostate cancers	C0376358
prostatic cancer	C0376358
prostatic cancers	C0376358
lymphoreticular neoplasm plasma cell	C0474853
malignant plasma cell neoplasm	C0474853
plasma cell neoplasm	C0474853
plasma cell sarcoma	C0474853
cancer of endometrium	C0476089
cancer of the endometrium	C0476089
carcinoma endometrial	C0476089
carcinoma of endometrium	C0476089
carcinoma of the endometrium	C0476089
endometrial ca	C0476089
endometrial cancer	C0476089
endometrial cancers	C0476089
endometrial carcinoma	C0476089
endometrial carcinomas	C0476089
endometrium ca	C0476089
endometrium cancer	C0476089
endometrium cancers	C0476089
endometrium carcinoma	C0476089
endometrium carcinomas	C0476089
uterine endometrial cancer	C0476089
appendiceal cancer	C0496779
appendix cancer	C0496779
appendix tumor malignant	C0496779
cancer appendix	C0496779
cancer of appendix	C0496779
cancer of the appendix	C0496779
malignant appendiceal neoplasm	C0496779
malignant appendix neoplasm	C0496779
malignant appendix tumor	C0496779
malignant neo appendix	C0496779
malignant neoplasm of appendix	C0496779
malignant neoplasm of appendix vermiformis	C0496779
malignant neoplasm of the appendix	C0496779
malignant tumor of appendix	C0496779
malignant tumor of the appendix	C0496779
malignant tumour of appendix	C0496779
ca eye	C0496836
cancer eye	C0496836
cancer eyes	C0496836
cancer of eye	C0496836
cancer of the eye	C0496836
cancers eye	C0496836
eye cancer	C0496836
eye cancers	C0496836
eye malignant tumors	C0496836
eyes cancer	C0496836
malign neopl eye nos	C0496836
malignant eye neoplasm	C0496836
malignant eye tumor	C0496836
malignant neoplasm of eye	C0496836
malignant neoplasm of eye nos	C0496836
malignant neoplasm of the eye	C0496836
malignant neoplasm of unspecified site of eye	C0496836
malignant neosplasm of the eye	C0496836
malignant ocular neoplasm	C0496836
malignant ocular tumor	C0496836
malignant tumor of eye	C0496836
malignant tumor of the eye	C0496836
malignant tumour of eye	C0496836
ocular cancer	C0496836
of eye cancer	C0496836
ca esophagus nos	C0546837
ca oesophagus nos	C0546837
cancer of esophagus	C0546837
cancer of oesophagus	C0546837
cancer of the esophagus	C0546837
esophageal cancer	C0546837
esophageal cancers	C0546837
esophageal malignant tumor	C0546837
esophagus cancer	C0546837
esophagus cancers	C0546837
mal neo esophagus nos	C0546837
malignant esophageal neoplasm	C0546837
malignant esophageal tumor	C0546837
malignant esophagus tumor	C0546837
malignant neoplasm of esophagus	C0546837
malignant neoplasm of esophagus nos	C0546837
malignant neoplasm of oesophagus	C0546837
malignant neoplasm of oesophagus nos	C0546837
malignant neoplasm of the esophagus	C0546837
malignant neosplasm of the esophagus	C0546837
malignant neosplasm of the oesophagus	C0546837

© 2021 Yuan Q et al. *JAMA Network Open.*

malignant tumor of esophagus	C0546837
malignant tumor of the esophagus	C0546837
malignant tumour of oesophagus	C0546837
oesophageal cancer	C0546837
oesophageal neoplasm	C0546837
oesophagus cancer	C0546837
cancer of the thyroid	C0549473
cancer of thyroid	C0549473
carcinoma of the thyroid	C0549473
carcinoma of the thyroid gland	C0549473
carcinoma of thyroid	C0549473
carcinoma of thyroid gland	C0549473
carcinoma thyroid	C0549473
carcinoma thyroid gland	C0549473
thyroid cancer	C0549473
thyroid cancers	C0549473
thyroid carcinoma	C0549473
thyroid carcinomas	C0549473
thyroid gland cancer	C0549473
thyroid gland carcinoma	C0549473
ewing sarcoma	C0553580
ewing tumor	C0553580
ewing's family of tumors	C0553580
ewing's sarcoma	C0553580
ewing's tumor	C0553580
ewing's tumors	C0553580
ewing's tumour	C0553580
ewings sarcoma	C0553580
ewings tumor	C0553580
ewings tumors	C0553580
ewings's sarcoma	C0553580
sarcoma ewing	C0553580
sarcoma ewing's	C0553580
sarcoma ewings	C0553580
cancer cell skin squamous	C0553723
cancers cell skin squamous	C0553723
carcinoma cell skin squamous	C0553723
cutaneous squamous cell carcinoma	C0553723
epidermoid carcinoma of skin	C0553723
epidermoid carcinoma of the skin	C0553723
epidermoid skin carcinoma	C0553723
skin squamous cell cancer	C0553723
skin squamous cell carcinoma	C0553723
spinous cell carcinoma	C0553723
squamous cell carcinoma of skin	C0553723
squamous cell carcinoma of the skin	C0553723
squamous cell carcinoma skin	C0553723
squamous cell skin cancer	C0553723
squamous cell skin carcinoma	C0553723
squamous skin carcinoma	C0553723
bone cancer	C0585442
bone osteosarcoma	C0585442
osteosarcoma	C0585442
osteosarcoma of bone	C0585442
cancer of larynx	C0595989
cancer of the larynx	C0595989
carcinoma laryngeal	C0595989
carcinoma larynx	C0595989
carcinoma of larynx	C0595989

carcinoma of the larynx	C0595989
larnyx carcinoma	C0595989
laryngeal cancer	C0595989
laryngeal carcinoma	C0595989
laryngeal throat cancer	C0595989
larynx carcinoma	C0595989
cancer of prostate	C0600139
cancer of the prostate	C0600139
carcinoma of prostate	C0600139
carcinoma of prostate gland	C0600139
carcinoma of the prostate	C0600139
carcinoma prostate	C0600139
carcinoma prostatic	C0600139
prostate cancer	C0600139
prostate carcinoma	C0600139
prostatic carcinoma	C0600139
cancer of the vulva	C0677055
cancer of vulva	C0677055
cancer vulva	C0677055
cancer vulvar	C0677055
carcinoma of the vulva	C0677055
carcinoma of vulva	C0677055
carcinoma vulva	C0677055
carcinoma vulvar	C0677055
vulva cancer	C0677055
vulva carcinoma	C0677055
vulvar cancer	C0677055
vulvar carcinoma	C0677055
cancer epithelial ovarian	C0677886
carcinoma of ovary	C0677886
carcinoma of the ovary	C0677886
carcinoma ovarian	C0677886
epithelial ovarian cancer	C0677886
epithelial ovarian cancers	C0677886
epithelial ovarian carcinoma	C0677886
epithelial ovarian carcinomas	C0677886
ovarian cancer	C0677886
ovarian cancer epithelial	C0677886
ovarian carcinoma	C0677886
ovarian epithelial cancer	C0677886
ovarian epithelial cancers	C0677886
ovarian epithelial carcinoma	C0677886
ovarian epithelial carcinomas	C0677886
breast cancer	C0678222
breast cancer diagnosis	C0678222
breast carcinoma	C0678222
breast carcinomas	C0678222
cancer of breast	C0678222
cancer of the breast	C0678222
carcinoma breast	C0678222
carcinoma of breast	C0678222
carcinoma of breast nos	C0678222
carcinoma of the breast	C0678222
mammary carcinoma	C0678222
adenocarcinoma of parathyroid	C0687150
adenocarcinoma of parathyroid gland	C0687150
adenocarcinoma of the parathyroid	C0687150
adenocarcinoma of the parathyroid gland	C0687150
cancer of parathyroid	C0687150

© 2021 Yuan Q et al. *JAMA Network Open.*

cancer of parathyroid gland	C0687150
cancer of the parathyroid	C0687150
cancer of the parathyroid gland	C0687150
carcinoma of parathyroid	C0687150
carcinoma of parathyroid gland	C0687150
carcinoma of the parathyroid	C0687150
carcinoma of the parathyroid gland	C0687150
carcinoma parathyroid	C0687150
parathyroid adenocarcinoma	C0687150
parathyroid cancer	C0687150
parathyroid cancers	C0687150
parathyroid carcinoma	C0687150
parathyroid carcinomas	C0687150
parathyroid gland adenocarcinoma	C0687150
parathyroid gland cancer	C0687150
parathyroid gland carcinoma	C0687150
prtc	C0687150
cancer of stomach	C0699791
cancer of the stomach	C0699791
carcinoma gastric	C0699791
carcinoma of stomach	C0699791
carcinoma of the stomach	C0699791
carcinoma stomach	C0699791
carcinomas gastric	C0699791
carcinomas stomach	C0699791
gastric cancer	C0699791
gastric carcinoma	C0699791
stomach cancer	C0699791
stomach carcinoma	C0699791
bladder cancer	C0699885
bladder carcinoma	C0699885
bladder carcinoma nos	C0699885
bladder carcinoma urinary	C0699885
cancer of bladder	C0699885
cancer of the bladder	C0699885
cancer of the urinary bladder	C0699885
cancer of urinary bladder	C0699885
carcinoma bladder	C0699885
carcinoma of bladder	C0699885
carcinoma of the bladder	C0699885
carcinoma of the urinary bladder	C0699885
carcinoma of urinary bladder	C0699885
carcinoma urinary bladder	C0699885
urinary bladder cancer	C0699885
urinary bladder carcinoma	C0699885
central neuroblastoma	C0700095
neuroblastoma	C0700095
cancer of the urethra	C0700101
cancer of urethra	C0700101
cancer urethra	C0700101
carcinoma of the urethra	C0700101
carcinoma of urethra	C0700101
carcinoma urethra	C0700101
urethra cancer	C0700101
urethra cancers	C0700101
urethra carcinoma	C0700101
urethral ca	C0700101
urethral cancer	C0700101
urethral cancers	C0700101

urethral carcinoma	C0700101
cancer cavity nasal	C0728864
cancer of the nasal cavity	C0728864
mal neo nasal cavities	C0728864
malignant nasal cavity neoplasm	C0728864
malignant nasal cavity tumor	C0728864
malignant neoplasm of nasal cavities	C0728864
malignant neoplasm of nasal cavities nos	C0728864
malignant neoplasm of nasal cavity	C0728864
malignant neoplasm of the nasal cavity	C0728864
malignant tumor of nasal cavity	C0728864
malignant tumor of the nasal cavity	C0728864
malignant tumour of nasal cavity	C0728864
nasal cavity cancer	C0728864
appendix cancer	C0728951
appendix carcinoma	C0728951
ca appendix	C0728951
carcinoma of appendix	C0728951
carcinoma of the appendix	C0728951
bile cancer duct	C0740277
bile duct cancer	C0740277
bile duct cancers	C0740277
bile duct carcinoma	C0740277
bile ducts cancer	C0740277
cancer bile duct	C0740277
cancer of bile duct	C0740277
cancer of the bile duct	C0740277
carcinoma bile duct	C0740277
carcinoma of bile duct	C0740277
carcinoma of the throat	C0740339
throat cancer	C0740339
throat carcinoma	C0740339
cancer neck	C0746787
cancer neck of	C0746787
cancer of neck	C0746787
cancer of the neck	C0746787
cancers neck	C0746787
malignant neck neoplasm	C0746787
malignant neck tumor	C0746787
malignant neck tumors	C0746787
malignant neoplasm of neck	C0746787
malignant neoplasm of neck nos	C0746787
malignant neoplasm of the neck	C0746787
malignant tumor of neck	C0746787
malignant tumor of the neck	C0746787
malignant tumour of neck	C0746787
neck cancer	C0746787
of neck cancer	C0746787
cancer of pharynx	C0747548
cancer of the pharynx	C0747548
carcinoma of pharynx	C0747548
carcinoma of the pharynx	C0747548
pharyngeal cancer	C0747548
pharyngeal carcinoma	C0747548
pharyngeal carcinoma cancer	C0747548
pharyngeal throat cancer	C0747548
pharynx carcinoma	C0747548
cancer head	C0751177
cancer of head	C0751177

cancer of the head	C0751177
cancers head	C0751177
head cancer	C0751177
malignant neoplasm of head	C0751177
malignant neoplasm of head nos	C0751177
of head cancer	C0751177
previously treated childhood rhabdomyosarcoma	C0796528
previously treated pediatric rhabdomyosarcoma	C0796528
rhabdomyosarcoma	C0796528
melanoma	C0796561
melanoma vaccine	C0796561
melanoma vaccines	C0796561
vaccine melanoma	C0796561
cancer of penis	C0853105
cancer of the penis	C0853105
carcinoma of penis	C0853105
carcinoma of the penis	C0853105
carcinoma penis	C0853105
penile cancer	C0853105
penile carcinoma	C0853105
penis carcinoma	C0853105
accessory sinus cancer	C0854995
accessory sinus carcinoma	C0854995
cancer of paranasal sinus	C0854995
cancer of the paranasal sinus	C0854995
carcinoma of accessory sinus	C0854995
carcinoma of paranasal sinus	C0854995
carcinoma of the accessory sinus	C0854995
carcinoma of the paranasal sinus	C0854995
paranasal sinus cancer	C0854995
paranasal sinus cancers	C0854995
paranasal sinus carcinoma	C0854995
sinus paranasal cancer	C0854995
malignant germ cell neoplasm of testis	C0855197
malignant germ cell neoplasm of the testis	C0855197
malignant germ cell tumor of testis	C0855197
malignant germ cell tumor of the testis	C0855197
malignant germ cell tumour of testis	C0855197
malignant testicular germ cell neoplasm	C0855197
malignant testicular germ cell tumor	C0855197
testicular cancer	C0855197
testicular germ cell cancer	C0855197
ca major salivary gland	C0948750
carcinoma gland salivary	C0948750
carcinoma glands salivary	C0948750
carcinoma of salivary gland	C0948750
carcinoma of the salivary gland	C0948750
salivary carcinoma	C0948750
salivary gland cancer	C0948750
salivary gland carcinoma	C0948750
malignant neopl rectum	C0949022
malignant neoplasm of rectum	C0949022
malignant neoplasm of the rectum	C0949022
malignant neosplasm of the rectum	C0949022
malignant rectal neoplasm	C0949022
malignant rectal tumor	C0949022
malignant tumor of rectum	C0949022

malignant tumor of the rectum	C0949022
malignant tumour of rectum	C0949022
rectal cancer	C0949022
rectal neoplasm malignant	C0949022
gestational trophoblastic disease	C1135868
gestational trophoblastic neopl	C1135868
gestational trophoblastic neoplasia	C1135868
gestational trophoblastic neoplasm	C1135868
gestational trophoblastic neoplasms	C1135868
gestational trophoblastic tumor	C1135868
gestational trophoblastic tumour	C1135868
gtt	C1135868
persistent gestational trophoblastic disease	C1135868
persistent trophoblastic disease	C1135868
pregnancy trophoblastic disease gestational neoplasia	C1135868
trophoblastic neopl gestational	C1135868
trophoblastic tumor gtt	C1135868
cancer of ovary	C1140680
cancer of the ovary	C1140680
malign neopl ovary	C1140680
malignant neoplasm of ovary	C1140680
malignant neoplasm of the ovary	C1140680
malignant ovarian neoplasm	C1140680
malignant ovarian tumor	C1140680
malignant tumor of ovary	C1140680
malignant tumor of the ovary	C1140680
malignant tumour of ovary	C1140680
ovarian ca	C1140680
ovarian cancer	C1140680
ovarian cancers	C1140680
ovary cancer	C1140680
ovary cancers	C1140680
adenocarcinoma duct	C1176475
carcinoma ductal	C1176475
carcinoma ducted	C1176475
carcinoma ducts	C1176475
carcinoma infiltrating duct	C1176475
carcinomas ductal	C1176475
duct adenocarcinoma	C1176475
duct carcinoma	C1176475
duct cell carcinoma	C1176475
ductal carcinoma	C1176475
ductal carcinomas	C1176475
infiltrating duct carcinoma	C1176475
malignant mesenchymal tumor	C1261473
malignant mesenchymal tumour	C1261473
sarcoma	C1261473
sarcoma nos	C1261473
sarcoma of soft tissue and bone	C1261473
sarcoma of the soft tissue and bone	C1261473
sarcomas	C1261473
soft tissue sarcoma	C1261473
soft tissue sarcomas	C1261473
pleuropulmonary blastoma	C1266144
ppb	C1266144
ppb familial tumor and dysplasia syndrome	C1266144
ppbftds	C1266144

© 2021 Yuan Q et al. *JAMA Network Open.*

pulmonary blastoma of childhood	C1266144
sarcoma	C1299262
adult lymphoma	C1332206
lymphoma	C1332206
childhood leukemia	C1332977
leukemia	C1332977
leukemia in children	C1332977
childhood lymphoma	C1332979
lymphoma	C1332979
pediatric lymphoma	C1332979
childhood osteogenic sarcoma	C1332986
childhood osteosarcoma	C1332986
osteosarcoma	C1332986
osteosarcoma in children	C1332986
pediatric osteosarcoma	C1332986
childhood kidney cell carcinoma	C1333001
childhood renal cell carcinoma	C1333001
pediatric kidney cell carcinoma	C1333001
pediatric renal cell carcinoma	C1333001
renal cell cancer	C1333001
cancer of hypopharynx	C1368404
cancer of the hypopharynx	C1368404
carcinoma of hypopharynx	C1368404
carcinoma of the hypopharynx	C1368404
hypopharyngeal cancer	C1368404
hypopharyngeal carcinoma	C1368404
hypopharyngeal throat cancer	C1368404
cancer of nasal cavity	C1377785
cancer of the nasal cavity	C1377785
carcinoma of nasal cavity	C1377785
carcinoma of the nasal cavity	C1377785
nasal cavity cancer	C1377785
nasal cavity carcinoma	C1377785
ppc	C1514428
primary peritoneal cancer	C1514428
primary peritoneal carcinoma	C1514428
colon rectal cancer	C1527249
colon/rectal cancer	C1527249
colorectal cancer	C1527249
colorectal cancers	C1527249
crc	C1527249
duct adenocarcinoma	C1527349
duct carcinoma	C1527349
ductal adenocarcinoma	C1527349
ductal breast carcinoma	C1527349
ductal carcinoma	C1527349
ductal carcinoma of breast	C1527349
ductal carcinoma of the breast	C1527349
basal cell carcinoma	C1547219
plasma cell dyscrasia	C1959632
plasma cell neoplasm	C1959632
plasma cell neoplasms	C1959632
plasma cell tumor	C1959632
plasmacytic neoplasm	C1959632
plasmacytic tumor	C1959632
plasmacytic tumour	C1959632
acute lymphoblastic leukemia	C1961102
acute lymphocytic leukemia	C1961102
acute lymphoid leukemia	C1961102

all1	C1961102
leukemia precursor cell lymphoblastic	C1961102
lymphoblastic leukemia	C1961102
lymphoblastic lymphoma	C1961102
precursor cell lymphoblastic leukaemia	C1961102
precursor cell lymphoblastic leukemia	C1961102
precursor cell lymphoblastic leukemia lymphoma	C1961102
adult liver cancer	C2239176
adult liver cancers	C2239176
carcinoma liver	C2239176
carcinoma of liver	C2239176
carcinoma of liver cell	C2239176
carcinoma of liver cells	C2239176
carcinoma of the liver cells	C2239176
hcc	C2239176
hepatic carcinoma	C2239176
hepatic cell carcinoma	C2239176
hepatocarcinoma	C2239176
hepatocellular cancer	C2239176
hepatocellular carcinoma	C2239176
hepatocellular carcinoma nos	C2239176
hepatocellular carcinoma of liver	C2239176
hepatocellular carcinomas	C2239176
hepatoma	C2239176
hepatomas	C2239176
lcc	C2239176
liver carcinoma	C2239176
liver cell cancer	C2239176
liver cell carcinoma	C2239176
liver cell carcinomas	C2239176
liver neoplasm malignant carcinoma	C2239176
liver neoplasm malignant carcinoma primary	C2239176
malignant hepatoma	C2239176
primary carcinoma of liver	C2239176
primary carcinoma of liver cells	C2239176
primary carcinoma of the liver cells	C2239176
cancer of oropharnyx	C2349952
cancer of oropharynx	C2349952
cancer of the oropharynx	C2349952
carcinoma of oropharynx	C2349952
carcinoma of the oropharynx	C2349952
oropharnyx cancer	C2349952
oropharnyx cancers	C2349952
oropharyngeal cancer	C2349952
oropharyngeal cancers	C2349952
oropharyngeal carcinoma	C2349952
oropharyngeal throat cancer	C2349952
oropharynx cancer	C2349952
oropharynx cancers	C2349952
oropharynx carcinoma	C2349952
diseases trophoblastic	C2931618
gestational trophoblastic disease	C2931618
gestational trophoblastic diseases	C2931618
trophoblastic disease	C2931618
trophoblastic disease nos	C2931618
cancer of nasopharynx	C2931822
cancer of the nasopharynx	C2931822

© 2021 Yuan Q et al. *JAMA Network Open.*

carcinoma nasopharyngeal	C2931822
carcinoma of nasopharynx	C2931822
carcinoma of the nasopharyngeal	C2931822
carcinoma of the nasopharynx	C2931822
carcinomas nasopharyngeal	C2931822
nasopharyngeal cancer	C2931822
nasopharyngeal carcinoma	C2931822
nasopharyngeal carcinomas	C2931822
nasopharyngeal throat cancer	C2931822
nasopharynx carcinoma	C2931822
npc	C2931822
npca	C2931822
pancreatic cancer	C2984259
pancreatic cancer pathway	C2984259
bladder cancer	C2984270
bladder cancer pathway	C2984270
colorectal cancer	C2984278
colorectal cancer pathway	C2984278
crc pathway	C2984278
melanoma	C2984289
melanoma pathway	C2984289
papillary thyroid carcinoma pathway	C2984307
ptc pathway	C2984307
thyroid cancer	C2984307
thyroid cancer pathway	C2984307
basal cell carcinoma	C2984322
basal cell carcinoma pathway	C2984322
prostate cancer	C2984325
prostate cancer pathway	C2984325
acute myeloid leukemia	C2984331
acute myeloid leukemia pathway	C2984331
aml pathway	C2984331
endometrial cancer	C2984333
endometrial cancer pathway	C2984333
998 myelodysplastic syndrome	C3463824
dysmyelopoiesis	C3463824
dysmyelopoietic syndrome	C3463824
dysmyelopoietic syndromes	C3463824
myelodysplasia	C3463824
myelodysplastic neoplasm	C3463824
myelodysplastic synd nos	C3463824
myelodysplastic syndrome	C3463824
myelodysplastic syndrome nos	C3463824
myelodysplastic syndrome/neoplasm	C3463824
myelodysplastic syndromes	C3463824
myeloid dysplasia	C3463824
oligoblastic leukemia	C3463824
preleukaemia	C3463824
preleukaemic syndrome	C3463824
preleukemia	C3463824
preleukemic syndrome	C3463824
smoldering leukemia	C3463824
smouldering leukaemia	C3463824
anal cancer	C3538805
rhabdomyosarcoma	C3538838
vulvar cancer	C3538899
melanoma	C3539018
cervical cancer	C3539064
gestational trophoblastic disease	C3539615

nasopharyngeal cancer	C3539713
salivary gland cancer	C3539927
small intestine cancer	C3539935
penile cancer	C3539940
vaginal cancer	C3539950
neuroblastoma	C3540583
retinoblastoma	C3540616
basal cell carcinoma	C3540686
prostate cancer	C3541264
hodgkin lymphoma	C3541281
thyroid cancer	C3541337
acute myeloid leukemia	C3541370
ewing sarcoma	C3541889
osteosarcoma	C3541899
parathyroid cancer	C3541904
acute lymphoblastic leukemia	C3542401
colorectal cancer	C3542412
squamous cell carcinoma of the skin	C3542432
uterine cancer	C3542435
basal cell cancer	C3811653
basal cell carcinoma	C3811653
basal cell epithelioma	C3811653
basal cell skin carcinoma	C3811653
bcc	C3811653
experimental organism basal cell carcinoma	C3811653
soft tissue sarcoma	C3898127
childhood langerhans cell histiocytosis	C3899655
langerhans cell histiocytosis	C3899655
adult langerhans cell histiocytosis	C3900100
langerhans cell histiocytosis	C3900100
ca cervix	C4048328
cancer of cervix	C4048328
cancer of the cervix	C4048328
cancer of the uterine cervix	C4048328
cervical ca	C4048328
cervical cancer	C4048328
cervical cancers	C4048328
cervix cancer	C4048328
uterine cervical cancer	C4048328
uterine cervical cancers	C4048328
uterine cervix cancer	C4048328
childhood neuroblastoma	C4086165
neuroblastoma	C4086165
adrenal gland chromaffin paraganglioma	C4551683
adrenal gland chromaffinoma	C4551683
adrenal gland paraganglioma	C4551683
adrenal gland pheochromocytoma	C4551683
adrenal medullary paraganglioma	C4551683
adrenal medullary pheochromocytoma	C4551683
adrenal neoplasm of uncertain behavior pheochromocytoma	C4551683
adrenal pheochromocytoma	C4551683
chromaffin paraganglioma	C4551683
chromaffin paraganglioma of the adrenal gland	C4551683
chromaffin tumor	C4551683
chromaffin tumour	C4551683
chromaffinoma	C4551683

intraadrenal paraganglioma	C4551683
pcc	C4551683
phaeochromocytoma	C4551683
pheochromocytoma	C4551683
pheochromocytoma syndrome	C4551683
pheochromocytomas	C4551683
cancer of the soft tissue	C4551686
malignant neoplasm of soft tissue	C4551686
malignant neoplasm of soft tissues	C4551686
malignant neoplasm of the soft tissue	C4551686
malignant neosplasm of the soft tissue	C4551686
malignant soft tissue neoplasm	C4551686
malignant soft tissue tumor	C4551686
malignant tumor of soft tissue	C4551686
malignant tumor of the soft tissue	C4551686
malignant tumour of soft tissue	C4551686
mesenchymal malignant tumor	C4551686
sarcoma	C4551686
sarcomas	C4551686
connective tissue sarcoma	C4551687
malignant soft tissue tumors	C4551687
sarcoma of soft tissue	C4551687
sarcoma of the soft tissue	C4551687
sarcoma soft tissue	C4551687
sarcomas soft tissue	C4551687
soft tissue sarcoma	C4551687
soft tissue sarcomas	C4551687

3. CUIs for histological type

Term	CUI
adenocarcinoma nos	C0001418
adenocarcinoma	C0001418
adenocarcinomas	C0001418
malignant adenomatous neoplasm	C0001418
cancer cell lung non small	C0007131
cancer cell lung non-small	C0007131
cancer cells lung non small	C0007131
carcinoma cell lung non small	C0007131
carcinoma cell lung non-small	C0007131
lung cancer non small cell	C0007131
lung cancer non-small cell	C0007131
lung carcinoma non small cell	C0007131
non small cell lung cancer	C0007131
non small cell lung cancer nos	C0007131
non small cell lung carcinoma	C0007131
non-oat cell lung cancer	C0007131
non-small cell cancer of lung	C0007131
non-small cell cancer of the lung	C0007131
non-small cell carcinoma of lung	C0007131
non-small cell carcinoma of the lung	C0007131
non-small cell lung cancer	C0007131
non-small cell lung carcinoma	C0007131
non-small-cell lung carcinoma	C0007131
non-small cell cancer of left lung	C0007131
non-small cell cancer of right lung	C0007131
nonsmall cell lung cancer	C0007131
non small cell cancer	C0007131
right lung non small cell cancer	C0007131

© 2021 Yuan Q et al. *JAMA Network Open.*

left lung non small cell cancer	C0007131
nsclc	C0007131
nsclc - non-small cell lung cancer	C0007131
small non cell lung cancer	C0007131
epidermoid carcinoma	C0007137
epidermoid cell cancer	C0007137
malignant epidermoid cell neoplasm	C0007137
malignant epidermoid cell tumor	C0007137
malignant squamous cell neoplasm	C0007137
malignant squamous cell tumor	C0007137
scc - squamous cell carcinoma	C0007137
squamous carcinoma	C0007137
squamous carcinomas	C0007137
squamous cell cancer	C0007137
squamous cell carcinoma nos	C0007137
squamous cell carcinoma	C0007137
squamous cell carcinomas	C0007137
squamous cell epithelioma	C0007137
cancer cell lung oat	C0149925
cancer cell lung small	C0149925

lung cancer oat cell	C0149925
lung cancer small cell	C0149925
lung malignant carcinoma oat cell	C0149925
lung small cell neuroendocrine carcinoma	C0149925
oat cell cancer	C0149925
oat cell carcinoma of lung	C0149925
oat cell lung cancer	C0149925
pulmonary small cell carcinoma	C0149925
pulmonary small cell carcinoma oat cell	C0149925
sclc - small cell lung cancer	C0149925
sclc	C0149925
small cell cancer of the lung	C0149925
small cell carcinoma of lung	C0149925
small cell carcinoma of the lung	C0149925
small cell carcinoma	C0149925
small cell lung cancer	C0149925
small cell lung carcinoma	C0149925
small cell neuroendocrine carcinoma of lung	C0149925
small cell neuroendocrine carcinoma of the lung	C0149925
oat cell cancer	C0262584

© 2021 Yuan Q et al. *JAMA Network Open.*

oat cell carcinoma syndrome	C0262584
oat cell carcinoma	C0262584
reserve cell carcinoma	C0262584
round cell carcinoma	C0262584
scc - small cell carcinoma	C0262584
small cell cancer	C0262584
small cell car	C0262584
small cell carcinoma nos	C0262584
small cell carcinoma	C0262584
small cell nec	C0262584
small cell neuroendocrine carcinoma	C0262584
large cell nec	C1265996
large cell neuroendocrine carcinoma	C1265996
large-cell neuroendocrine carcinoma	C1265996
large cell cancer	C1265996
large cell carcinoma	C1265996
large cell lung cancer	C1265996
large cell lung carcinoma	C1265996
non small cell carcinoma	C1266002
non-small cell carcinoma	C1266002
small cell carcinoma non	C1266002
small cell carcinoma-non	C1266002

© 2021 Yuan Q et al. *JAMA Network Open.*

4. Terms for clinical stage

Term	Stage
stage 1	stage 1
stage 1a	stage 1a
stage 1a1	stage 1a1
stage 1a2	stage 1a2
stage 1b	stage 1b
stage 1b1	stage 1b1
stage 1b2	stage 1b2
stage 1c	stage 1c
stage 1m	stage 1m
stage 1s	stage 1s
stage 2	stage 2
stage 2a	stage 2a
stage 2b	stage 2b
stage 2c	stage 2c
stage 3	stage 3
stage 3a	stage 3a
stage 3b	stage 3b
stage 3c	stage 3c
stage 4	stage 4
stage 4a	stage 4a
stage 4b	stage 4b
stage 4c	stage 4c
stage 4s	stage 4s
stage i	stage 1
stage ia	stage 1a
stage ia1	stage 1a1
stage ia2	stage 1a2
stage ia3	stage 1a3
stage ib	stage 1b
stage ic	stage 1c
stage ic	stage 1c
stage ii	stage 2
stage iia	stage 2a
stage iib	stage 2b
stage iic	stage 2c
stage iii	stage 3
stage iiia	stage 3a
stage iiia1	stage 3a1
stage iiia2	stage 3a2
stage iiib	stage 3b
stage iiic	stage 3c
stage iiid	stage 3d
stage im	stage 1m
stage is	stage 1s
stage iv	stage 4
stage metastatic	stage 4
end stage	stage 4
stage iv lower case c	stage 4c
stage iva	stage 4a
stage iva1	stage 4a1
stage iva2	stage 4a2
stage ivb	stage 4b
stage ivc	stage 4c
stage ivs	stage 4s
stage level 1	stage 1
stage level 2	stage2
stage level 3	stage 3
stage level 4	stage 4
extensive stage	extensive stage
early stage	limited stage
limited stage	limited stage

© 2021 Yuan Q et al. *JAMA Network Open.*

5. CUIs for somatic mutations

Term	CUI
2 her	C0242957
activated hepatocyte growth factor receptor gene	C1417123
afterbrain	C0376353
akt	C0812228
akt alpha protein	C0285558
akt1	C0285558
akt1	C0812228
akt1 gene	C0812228
akt1 protein kinase	C0285558
alk	C1332080
alk	C1663627
alk	C1704943
alk gene	C1332080
alk tyrosine kinase receptor	C1663627
alk wt allele	C1704943
alk/eml4 fusion gene	C1332080
alk/npm1 fusion gene	C1332080
alps5	C1705969
anaplastic lymphoma kinase	C1332080
anaplastic lymphoma kinase	C1663627
anaplastic lymphoma receptor tyrosine kinase	C1332080
antigen cd340	C1702024
area pd of bonin	C3496524
armadillo	C1332803
auts9	C1704823
avian ur2 sarcoma virus oncogene homolog 1	C0812281
b7 homolog 1	C0965245
b7 homolog 1	C1540292
b7h1	C1540292
b7h1	C3272500
bcc7	C1705526
beta catenin	C0105770
bpes1	C1412815
braf	C0812241
braf	C1259929
braf gene	C0812241
braf genes	C0812241
braf protein	C1259929
braf/kiaa1549 fusion gene	C0812241
braf1	C0812241
braf1 protein	C1259929
c erbb 1 protein	C0034802
c erbb 2 genes	C0242957
c erbb 2 protein	C0069515
c erbb 2 proto oncogenes	C0242957
c erbb genes 002	C0242957
c erbb protein 001	C0034802
c erbb protein 002	C0069515
c ki ras p 021	C0079973
c ki ras p21	C0079973
catenin beta 1	C1332803
cd	C1705969
cd antigen cd274	C0965245
cd152	C1705969
cd152	C2350360
cd152 antigen	C0111208
cd152 antigen	C2350360
cd152 antigen gene	C1705969
cd152 antigens	C0111208
cd152 gene	C1705969
cd246	C1332080
cd246 antigen	C1663627
cd274	C0965245
cd274	C1540292
cd274 antigen	C0965245
cd274 antigen gene	C3272500
cd274 gene	C1540292
cd274 molecule	C1540292
cd274 molecule gene	C1540292
cd274 molecule wt allele	C3272500
cd274 wt allele	C3272500
cd279	C1418401
cd279 antigen	C1418401
cd340	C0242957
cd340	C1702024
cd340 antigen	C0069515
cd340 antigen	C1702024
cd340 antigens	C0069515
celiac3	C1705969
cell growth inhibiting protein 40	C1739039
cellular tumor antigen p53	C0080055
chronic ulcerative stomatitis protein	C1431519
cohesin subunit sa2	C1420449
ctla 4	C0111208
ctla 4 antigen	C0111208
ctla4	C1705969
ctla4	C2350360
ctla4 protein	C2350360
ctla4 wt allele	C1705969
ctnnb	C1332803
ctnnb1	C0105770
ctnnb1	C1332803
ctnnb1 gene	C1332803
cusp	C1431519
cytotoxic t lymphocyte antigen 4	C0111208
cytotoxic t lymphocyte associated antigen 4	C0111208
deficiency of methionine adenosyltransferase	C0268621
deleted in pancreatic carcinoma 4	C0694891
dfnb97	C1417123
dfnb97	C1704823
disease progression	C1335499
dpc4	C0694891
ec 2.7.1.153	C1451005
ec 2.7.10.1	C1310668
ec 2.7.10.1	C1663627
ec 2.7.10.1	C1702024
ec 2.7.10.1	C1739039
ec 2.7.11.1	C1259929
ec 2.7.11.1	C1431123
ec 2.7.11.1	C1451005

© 2021 Yuan Q et al. *JAMA Network Open.*

eec3	C1422009
egf recept	C0034802
egf receptor	C0034802
egf receptor	C1739039
egf receptor activity	C1150617
egf receptors	C0034802
egfr	C0034802
egfr	C1150617
egfr	C1368111
egfr	C1414313
egfr	C1739039
egfr	C3811844
egfr	C3812682
egfr ecd	C1368111
egfr extracellular domain	C1368111
egfr gene	C1414313
epidermal factor growth receptors	C0034802
epidermal growth factor recept	C0034802
epidermal growth factor receptor	C0034802
epidermal growth factor receptor	C1414313
epidermal growth factor receptor	C1739039
epidermal growth factor receptor	C3812682
epidermal growth factor receptor activity	C1150617
epidermal growth factor receptor extracellular domain	C1368111
epidermal growth factor receptor kinase	C0034802
epidermal growth factor receptor measurement	C3812682
epidermal growth factor receptor protein tyrosine kinase	C0034802
erbb 1 proto oncogene protein	C0034802
erbb 2 genes	C0242957
erbb 2 proto oncogene protein	C0069515
erbb 2 receptor protein tyrosine kinase	C0069515
erbb genes 002	C0242957
erbb protein	C1739039
erbb proto oncogene protein 001	C0034802
erbb proto oncogene protein 002	C0069515
erbb receptor protein tyrosine kinase 002	C0069515
erbb1	C1414313
erbb1	C1739039
erbb1	C3812682
erbb2	C0069515
erbb2	C0242957
erbb2	C1702024
erbb2	C1704824
erbb2	C3810543
erbb2 gene	C0242957
erbb2 genes	C0242957
erbb2 wt allele	C1704824
estimated glomerular filtration rate	C3811844
fasciculus predorsalis	C0175542
flj13213	C1822773
flj92943	C1705526
forkhead box l2	C1412815
forkhead box l2 gene	C1412815
forkhead box protein l2	C2700208

forkhead transcription factor foxl2	C1412815
forkhead transcription factor foxl2	C2700208
foxl2	C1412815
foxl2	C2700208
foxl2 gene	C1412815
fulfill	C1550543
gene p53	C0079419
genes erbb 002	C0242957
genes her 002	C0242957
genes p 053	C0079419
genes tp 053	C0079419
gfre	C3811844
gp280	C1310668
gpbp kinase activity	C2247364
gpbpk	C2247364
grd4	C1705969
gse	C1705969
hep methion adenosyltransf def	C0268621
hepatic methionine adenosyltransferase deficiency	C0268621
hepatocyte growth factor receptor	C1417123
hepatocyte growth factor receptor gene	C1417123
hepatocyte growth factor receptor gene	C1704823
her 2	C0242957
her 2 neu	C0242957
her 2 proto oncogene protein	C0069515
her proto oncogene protein 002	C0069515
her1	C1414313
her1	C1739039
her1	C3812682
her2	C0069515
her2	C0242957
her2	C1702024
her2	C1704824
her2	C3810543
her2 gene	C0242957
her2 genes	C0242957
her2/neu	C1702024
her2/neu	C1704824
her2/neu	C3810543
herstatin	C0242957
hgfr	C1417123
hgfr gene	C1704823
hlkb1	C1431123
hsle1	C1418401
human egf receptor 2	C1702024
human egf receptor 2 gene	C1704824
human epidermal growth factor receptor 2	C0242957
human epidermal growth factor receptor 2	C1702024
human epidermal growth factor receptor 2	C3810543
human epidermal growth factor receptor 2 measurement	C3810543
hypermethioninaemia	C0268621
hypermethioninemia	C0268621
icos	C1705969
iddm12	C1705969

© 2021 Yuan Q et al. *JAMA Network Open.*

increased methionine in blood	C0268621
isolated persistent hypermethioninemia	C0268621
k ras genes	C1537502
k ras oncogenes	C0022457
keratinocyte transcription factor ket	C1431519
ket	C1422009
kirsten murine sarcoma virus 2	C1537502
kirsten ras1	C1537502
kirsten rat sarcoma viral oncogene homolog	C1537502
krab protein	C1259929
kras	C0022457
kras	C0079973
kras	C1537502
kras gene	C1537502
kras1	C1537502
kras1p	C1537502
kras2	C0079973
kras2	C1537502
l methionine	C0025646
lfs1	C0079419
lfs1	C1705526
liver kinase b1	C1431123
lkb1	C0694883
lkb1	C1431123
madh4	C0694891
mat deficiency	C0268621
mat i/iii deficiency	C0268621
mcf3	C0812281
mechanism of action	C1524059
mechanism of action qualifier	C1524059
meet	C1550543
met	C0025646
met	C0268621
met	C0376353
met	C0428210
met	C1417123
met	C1419433
met	C1550543
met	C1704823
met	C1822773
met	C2983100
met gene	C1417123
met oncogene	C1417123
met protooncogene	C1417123
met wt allele	C1704823
metabolic equivalent of task	C1419433
metabolic equivalent of task	C2983100
metabolic equivalent of task unit	C1419433
metastatic lymph node gene 19 protein	C0069515
metastatic lymph node gene 19 protein	C1702024
metencephalon	C0376353
metencephalons	C0376353
methioninaemia	C0268621
methionine	C0025646
methionine	C0428210
methionine adenosyltransferase deficiency	C0268621
methionine level	C0428210

methionine measurement	C0428210
methionine preparation	C0025646
methionine product	C0025646
methionine test	C0428210
methioninemia	C0268621
mgc142294	C3272500
mgc142296	C3272500
mln 19	C1702024
mode of action	C1524059
n ras genes	C0027260
n ras oncogenes	C0027260
nbp	C1422009
neu	C0069515
neu	C0242957
neu	C1702024
neu gene	C0242957
neu gene	C1704824
neu genes	C0242957
neu proto oncogene protein	C0069515
neu receptor	C0069515
neu receptors	C0069515
neuro/glioblastoma derived oncogene homolog	C0242957
neuroblastoma ras viral oncogene homolog	C0027260
neuroblastoma ras viral oncogene homolog	C0809246
neuroblastoma ras viral oncogene homolog gene	C0809246
ngl	C0242957
ngl	C1704824
nras	C0027260
nras	C0079975
nras	C0809246
nras gene	C0809246
nras1	C0027260
nras1	C0809246
ofc8	C1422009
oncogene akt1	C0812228
oncogene braf	C0812241
oncogene erbb	C1414313
oncogene erbb2	C0242957
oncogene hgfr	C1417123
oncogene kras1	C1537502
oncogene kras2	C1537502
oncogene met	C1417123
oncogene nras	C0809246
oncogene protein her 2	C0069515
oncogene ros	C0812281
oncoprotein p53	C0080055
p a 021 c ki ras	C0079973
p a 021 n ras	C0079975
p a 053 genes	C0079419
p a 053 tumor suppressor protein	C0080055
p110alpha	C1451005
p185erbb2	C1702024
p185erbb2 protein	C0069515
p21 c k ras	C0079973
p21 c ki ras	C0079973

© 2021 Yuan Q et al. *JAMA Network Open.*

p21c ki ras	C0079973
p21n ras	C0079975
p51	C1422009
p51	C1431519
p53	C0079419
p53	C0080055
p53	C1705526
p53 antigen	C0080055
p53 gene	C0079419
p53 gene	C1705526
p53 genes	C0079419
p53 oncogene	C0079419
p53 oncoprotein	C0080055
p53 protein	C0080055
p53 proteins	C0080055
p53 tumor suppressor	C0079419
p53 tumor suppressor	C0080055
p53 tumor suppressor gene	C0079419
p53 tumor suppressor protein	C0080055
p53cp	C1422009
p63	C1422009
p63	C1431519
p73h	C1422009
p73l	C1422009
p73l	C1431519
p94	C1259929
palladium	C0030230
palladium metallicum	C0030230
palladium metallicum / palladium	C0030230
palladium metallicum/palladi	C0030230
park	C3160718
parkinson disease late onset	C3160718
pd	C0030230
pd	C0175542
pd	C1335499
pd	C1524059
pd	C2362652
pd	C3160718
pd	C3496524
pd element	C0030230
pd1	C1418401
pdcd1	C1418401
pdcd1 gene	C1418401
pdcd1 ligand 1	C0965245
pdcd1 ligand 1	C1540292
pdcd1l1	C1540292
pdcd1l1	C3272500
pdcd1lg1	C1540292
pdcd1lg1	C3272500
pdl1	C1540292
pdl1	C3272500
pfrk	C1412815
pharmacol	C1524059
pharmacology	C1524059
phosphoprotein p53	C0080055
pi3k	C1335212
pi3k	C1451005
pi3kalpha	C1451005
pik3ca	C1335212
pik3ca	C1451005
pik3ca gene	C1335212
pjs	C0694883
pkb	C0285558
pkb	C0812228
pkb protein	C0285558
pons and cerebellum	C0376353
pons et cerebellum	C0376353
possible	C2362652
possible diagnosis	C2362652
possibly	C2362652
pp53 phosphoprotein	C0080055
predorsal bundle	C0175542
predorsal bundle of edinger	C0175542
predorsal bundles	C0175542
predorsal fasciculus	C0175542
prkba	C0285558
prkba	C0812228
pro2286	C0105770
programmed cell death 1	C1418401
programmed cell death 1 gene	C1418401
programmed cell death 1 ligand 1	C0965245
programmed cell death 1 ligand 1	C1540292
programmed cell death 1 ligand 1 gene	C3272500
programmed death ligand 1	C0965245
programmed death ligand 1	C1540292
progressive disease	C1335499
protein kinase b	C0285558
protein kinase b alpha	C0285558
protein p53	C0080055
proto oncogene c erbb 1 protein	C0034802
proto oncogene c erbb 2	C0069515
proto oncogene protein her 002	C0069515
proto oncogene protein her 2	C0069515
proto oncogene protein neu	C0069515
proto oncogene protein p 185 neu	C0069515
proto oncogene proteins c akt1	C0285558
proto oncogene proteins c erbb 2	C0069515
rac	C0285558
rac	C0812228
rac pk alpha protein	C0285558
rac serine/threonine protein kinase	C0812228
rafb1	C0812241
rafb1 protein	C1259929
rask1	C1537502
rask2	C1537502
rccp2	C1417123
rccp2	C1704823
recept egf	C0034802
recept epidermal growth factor	C0034802
recept tgf alpha	C0034802
recept transforming growth factor alpha	C0034802
recept urogastrone	C0034802
resceptor tyrosine protein kinase erbb 1	C0034802
rg7mt1	C1419433
rnmt	C1419433
rnmt gene	C1419433
ros	C0812281
ros1	C0812281

ros1	C1310668
ros1 gene	C0812281
ros1 protein	C1310668
sa2	C1420449
sa7	C1414313
safb like transcription modulator	C1822773
scc3b	C1420449
segfr	C1368111
serine/threonine kinase 11	C0694883
serine/threonine kinase 11 gene	C0694883
serine/threonine protein kinase 11	C0694883
serine/threonine protein kinase 11	C1431123
serine/threonine protein kinase pik3ca	C1451005
serine/threonine protein kinase stk11	C1431123
shfm4	C1422009
sltm	C1822773
sltm gene	C1822773
smad family member 4	C0694891
smad4	C0694891
smad4 gene	C0694891
soluble epidermal growth factor receptor	C1368111
species antigen 7	C1414313
stag2	C1420449
stag2 gene	C1420449
stk11	C0694883
stk11	C1431123
stk11	C2247364
stk11 gene	C0694883
stk11/lkb1 protein	C1431123
stromal antigen 2	C1420449
stromal antigen 2 gene	C1420449
tectospinal fiber	C0175542
tectospinal fibers	C0175542
tgf alpha receptor	C0034802
tkr1	C0242957
tkr1	C1704824

tp a 053 genes	C0079419
tp53	C0079419
tp53	C0080055
tp53	C1705526
tp53 gene	C0079419
tp53 genes	C0079419
tp53 protein	C0080055
tp53 wt allele	C1705526
tp63	C1422009
tp63	C1431519
tp63 gene	C1422009
tp73l	C1422009
tp73l gene	C1422009
transforming growth factor alpha recept	C0034802
transforming growth factor alpha receptor	C0034802
trp53	C0079419
trp53	C1705526
trp53 protein	C0080055
tumor protein 63	C1431519
tumor protein p53	C0079419
tumor protein p53	C0080055
tumor protein p53 gene	C1705526
tumor protein p63	C1422009
tumor protein p63 gene	C1422009
tumor suppressor p53	C0079419
tumor suppressor p53	C0080055
tumor suppressor protein p 053	C0080055
tumor suppressor protein p53	C0080055
tyrosine kinase type cell surface receptor her2	C0069515
urogastrone recept	C0034802
urogastrone receptor	C0034802
urogastrone receptor	C1739039

© 2021 Yuan Q et al. *JAMA Network Open.*

eTable 3. Completeness of Variables in Final Cohort

	Completeness (% having at least one measurement)	
Time-invariant variable		
Birthday	100	
Sex	100	
Race	92.2	
Diagnosis date	100	
OS	100	
Stage	75.9	
Histology type	87.1	
Time-variant variable		
	Total[a]	Baseline[b]
BMI	49.6	38.9
ECOG	30.4	15.4
Laboratory test [c]	82.5	65.3

[a] Data available at any time
[b] Data available within 3 months before or after diagnosis date
[c] At least one measurement for common laboratory tests included complete blood count, metabolic panel, lipid panel, liver panel, hemoglobin A1C and urinalysis.

© 2021 Yuan Q et al. *JAMA Network Open.*

eTable 4. Discrepancies Between Electronic Health Record Diagnosis Date and Random Samples and Boston Lung Cancer Study Diagnosis Date

Absolute discrepancy	Combine ICD date and NICE extracted dates[a]		ICD Date[b]	
	Random samples	BLCS	Random samples	BLCS
<90 days	89.6% (60/67)	87.6%	86.6% (58/67)	87.5%
<180 days	91.0% (61/67)	91.2%	88.1% (59/67)	91.1%
<1 year	92.5% (62/67)	93.6%	91.0% (60/67	93.6%

[a] The earlier of date of the first ICD code and date mentioned in clinical notes was used as a proxy for date of diagnosis.
[b] The date of the first ICD code was used as a proxy for date of diagnosis.
Combining ICD dates and dates mentioned in clinical notes have higher concordance compared to using ICD dates only

© 2021 Yuan Q et al. *JAMA Network Open.*

eTable 5. Histologic Type From Boston Lung Cancer Study Cohort and Random Samples vs From Electronic Health Records

	BLCS				Random samples			
EMR	Adeno	Squamous	NSCLC	SCLC	Adeno	Squamous	NSCLC unspecified	SCLC
Adeno	3386	64	185	3	38	0	0	0
Squamous	44	951	46	0	0	8	0	0
NSCLC unspecified	95	41	296	2	2	1	4	0
SCLC	8	16	12	477	0	0	0	8
Total	3533	1072	539	482	40	9	4	8
Accuracy	0.96	0.89	0.55	0.99	0.95	0.89	1.00	1.00

© 2021 Yuan Q et al. *JAMA Network Open.*

eTable 6. Stage From Boston Lung Cancer Study Cohort and Random Samples vs From Electronic Health Records

	BLCS						Random samples					
EMR	Stage1	Stage 2	Stage 3	Stage 4	Extensive	Limited	Stage1	Stage 2	Stage 3	Stage 4	Extensive	Limited
Stage 1	1287	89	37	49	0	1	9	1	0	0	0	0
Stage 2	160	308	24	19	0	0	3	6	1	1	0	0
Stage 3	84	47	955	63	1	0	1	0	13	1	0	0
Stage 4	145	45	94	1302	1	0	1	1	0	14	0	0
Extensive	2	0	2	12	207	11	0	0	0	0	2	1
Limited	25	9	15	8	14	174	0	0	0	0	0	2
Total	1703	498	1127	1453	223	186	14	8	14	16	2	3
Accuracy	0.76	0.62	0.85	0.90	0.93	0.94	0.64	0.75	0.93	0.88	1.00	0.67

© 2021 Yuan Q et al. *JAMA Network Open.*

eTable 7. Multivariate Cox Proportional Hazards Regression for Patients With Non–Small Cell Lung Cancer in Boston Lung Cancer Study and Electronic Health Record Data

	BLCS cohort (n=5056)		EMR cohort overlapped with BLCS cohort (n=4,377)		EMR cohort (n=23,420)	
	HR	P-value	HR	P-value	HR	P-value
Age at diagnosis	1.02	<0.001	1.02	<0.001	1.02	<0.001
Sex						
Female	ref					
Male	1.38	0.002	1.31	<0.001	1.24	<0.001
Race						
White	ref					
Other	0.93	0.45	0.88	0.21	0.96	0.27
Smoking status						
Never smoker	ref					
Smoker	1.41	<0.001	1.80	<0.001	1.68	<0.001
Type						
Adenocarcinoma	ref					
Squamous cell	1.42	<0.001	1.35	<0.001	1.21	<0.001
Others	1.29	<0.001	1.71	<0.001	1.77	<0.001
Stage						
1	ref					
2	1.50	<0.001	1.52	<0.001	1.67	<0.001
3	2.78	<0.001	3.05	<0.001	2.77	<0.001
4	6.22	<0.001	5.43	<0.001	4.89	<0.001

Note: Patients with complete data on age, sex, stage, type, race, and smoking status were included in the analysis. We assessed the overall data quality by comparing hazard ratio (HR) estimates from fitting Cox models for OS to the BLCS cohort, EMR cohort overlapped with BLCS cohort and also EMR cohort.

© 2021 Yuan Q et al. *JAMA Network Open.*

eTable 8. Multivariate Cox Proportional Hazards Regression for Patients With Small Cell Lung Cancer in Boston Lung Cancer Study and Electronic Health Record Data

	BLCS cohort (n=475)		EMR cohort overlapped with BLCS cohort (n=412)		EMR cohort (n=3356)	
	HR	P-value	HR	P-value	HR	P-value
Age at diagnosis	1.02	<0.001	1.02	<0.001	1.02	<0.001
Sex						
Female	ref					
Male	1.38	0.002	1.31	0.02	1.12	0.01
Race						
White	ref					
Other	1.44	0.22	1.08	0.83	1.07	0.45
Smoking status						
Never smoker	ref					
Smoker	1.30	0.57	1.78	0.32	1.55	<0.001
Stage						
limited	ref					
extensive	2.89	<0.001	2.78	<0.001	2.60	<0.001

Note: Patients with complete data of age, sex, stage, type, race, and smoking status were included in the analysis. We assessed the overall data quality by comparing hazard ratio (HR) estimates from fitting Cox models for OS to the BLCS cohort, EMR cohort overlapped with BLCS cohort and also EMR cohort.

© 2021 Yuan Q et al. *JAMA Network Open.*

eTable 9. Basic Characteristics of Patients in Non–Small Cell Lung Cancer Prognostic Model

Patient characteristic	n=11724
Age, median, (years)	66.88
Sex	
Female	6,306 (53.8)
Male	5,418 (46.2)
Race	
White	10,798 (92.1)
Others	926 (7.9)
Smoking	
Smoker	11,048 (94.2)
Nonsmoker	676 (5.8)
Histological Type	
Adenocarcinoma	7,985 (68.1)
Squamous	2,504 (21.4)
NSCLC not specified	1,235 (10.5)
Stage	
1	3,513 (30.0)
2	1,521 (13.0)
3	2,682 (22.9)
4	4,008 (34.2)
BMI	
Reference range	3,088 (26.3)
Obesity	2,014 (17.2)
Overweight	2,994 (25.5)
Underweight	259 (2.2)
Missing	3,369 (28.7)
History of COPD	
No	9,082 (77.5)
Yes	2,642 (22.5)
History of asthma	
No	9,280 (79.2)
Yes	2,444 (20.8)
History of diabetes	
No	11,623 (99.1)
Yes	101 (0.9)

© 2021 Yuan Q et al. *JAMA Network Open.*

eTable 10. Values of Laboratory Variables for Patients in Non–Small Cell Lung Cancer Prognostic Model

Patient characteristic		n=11724
Albumin	≤3.5 g/dL	2079 (17.7)
	>3.5 g/dL	7661 (65.3)
	Missing	1984 (16.9)
ALKP	≤140 IU/L	9488 (80.9)
	>140 IU/L	870 (7.4)
	Missing	1366 (11.7)
ALT	≤7 IU/L	405 (3.5)
	≥56 IU/L	511 (4.4)
	7-56 IU/L	9383 (80.0)
	Missing	1425 (12.2)
AST	≤10 IU/L	193 (1.6)
	≥40 IU/L	1006 (8.6)
	10-40 IU/L	9060 (77.3)
	Missing	1465 (12.5)
BUN	≥7 mg/dL	451 (3.8)
	≥20 mg/dL	3081 (26.3)
	7-20 mg/dL	7771 (66.3)
	Missing	421 (3.6)
Calcium	≤8.5 mg/dL	1879 (16.0)
	≥10.5 mg/dL	338 (2.9)
	8.5-10.5 mg/dL	8833 (75.3)
	Missing	674 (5.7)
Chloride	≤96 mEq/L	1097 (9.4)
	≥106 mEq/L	1750 (14.9)
	96-106 mEq/L	8354 (71.3)
	Missing	523 (4.5)
Creatinine	Above	1981 (16.9)
	Normal	388 (3.3)
	Low	8924 (76.1)
	Missing	431 (3.7)
Glucose	≤100 mg/dL	4386 (37.4)
	≥125 mg/dL	3633 (31.0)
	100-125 mg/dL	3122 (26.6)
	Missing	583 (5.0)
HBG	Above	156 (1.3)
	Normal	384 (3.3)
	Low	6598 (56.3)
	Missing	4586 (39.1)
MCH	≤27 pg/cell	1024 (8.7)
	≥33 pg/cell	894 (7.6)
	27-33 pg/cell	9419 (80.3)
	Missing	387 (3.3)
MCHC	≤31 g/dL	229 (2.0)
	≥37 g/dL	32 (0.3)
	31-37 g/dL	11079 (94.5)
	Missing	384 (3.3)
MCV	≤80 femtoliters/cell	649 (5.5)
	≥96 femtoliters/cell	1254 (10.7)
	80-96 femtoliters/cell	9435 (80.5)
	Missing	386 (3.3)

© 2021 Yuan Q et al. *JAMA Network Open.*

PLT	≤150000/mL	521 (4.4)
	≥450000/mL	1083 (9.2)
	150000-450000/mL	9721 (82.9)
	Missing	399 (3.4)
Potassium	≤3.5 mEq/L	1176 (10.0)
	≥5.0 mEq/L	428 (3.7)
	3.5-5.0 mEq/L	9462 (80.7)
	Missing	658 (5.6)
RBC	Normal	385 (3.3)
	Low	5023 (42.8)
	MISSING	6316 (53.9)
RDW	≤14.5%	8691 (74.1)
	>14.5%	2637 (22.5)
	Missing	396 (3.4)
Sodium	≤135 mEq/L	1819 (15.5)
	≥145 mEq/L	277 (2.4)
	135-145 mEq/L	9110 (77.7)
	Missing	518 (4.4)
Bilirubin	≤0.2 mg/dL	1322 (11.3)
	>1.2 mg/dL	226 (1.9)
	0.2-1.2 mg/dL	8797 (75.0)
	Missing	1379 (11.8)
WBC	>11 000 count, per µL	8489 (72.4)
	4500-11 000count, per µL	2859 (24.4)
	Missing	376 (3.2)
Lymphocytes	≤20%	5484 (46.8)
	≥40%	302 (2.6)
	20-40%	4104 (35.0)
	Missing	1834 (15.6)
Neutrophils	≤40%	106 (0.9)
	≥60%	8365 (71.3)
	40-60%	1419 (12.1)
	Missing	1834 (15.6)
Monocytes	≤2%	457 (3.9)
	≥8%	2073 (17.7)
	2-8%	7358 (62.8)
	Missing	1836 (15.7)
Eosinophils	≤1%	3683 (31.4)
	≥4%	1612 (13.7)
	1-4%	4584 (39.1)
	Missing	1845 (15.7)
Basophils	≤0.5%	6293 (53.7)
	≥1%	2025 (17.3)
	0.5-1%	1538 (13.1)
	Missing	1868 (15.9)
NLR	≤4	5262 (44.9)
	>4	4621 (39.4)
	Missing	1841 (15.7)

© 2021 Yuan Q et al. *JAMA Network Open.*

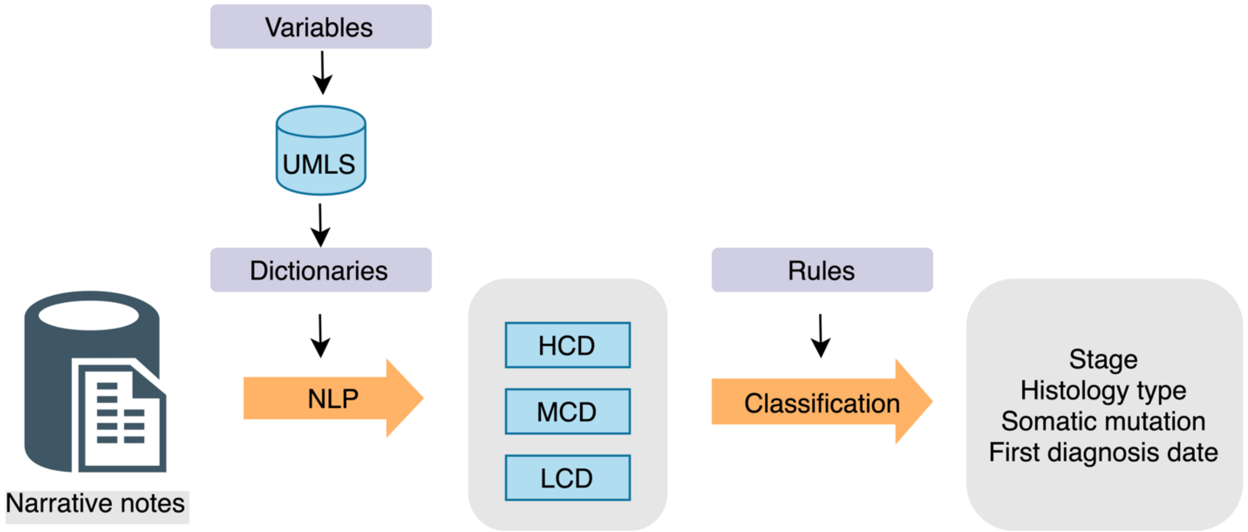

eFigure 1. Flow Chart of Natural Language Processing Interpreter for Cancer Extraction (NICE)

The first step is to build dictionaries for variables of interest using UMLS. Then process narrative notes to identify data in notes with different confidence levels for each variable. Using knowledge-based rules to obtain final result for each variable.

HCD-High confidence data, with mention of lung cancer concepts in the same sentence
MCD-Medium confidence data, with mention of lung cancer concepts in the same note
LCD-Low confidence data, with no mention of lung cancer concepts in the same note
NLP-Natural Language Processing; UMLS-Unified Medical Language System

© 2021 Yuan Q et al. *JAMA Network Open.*

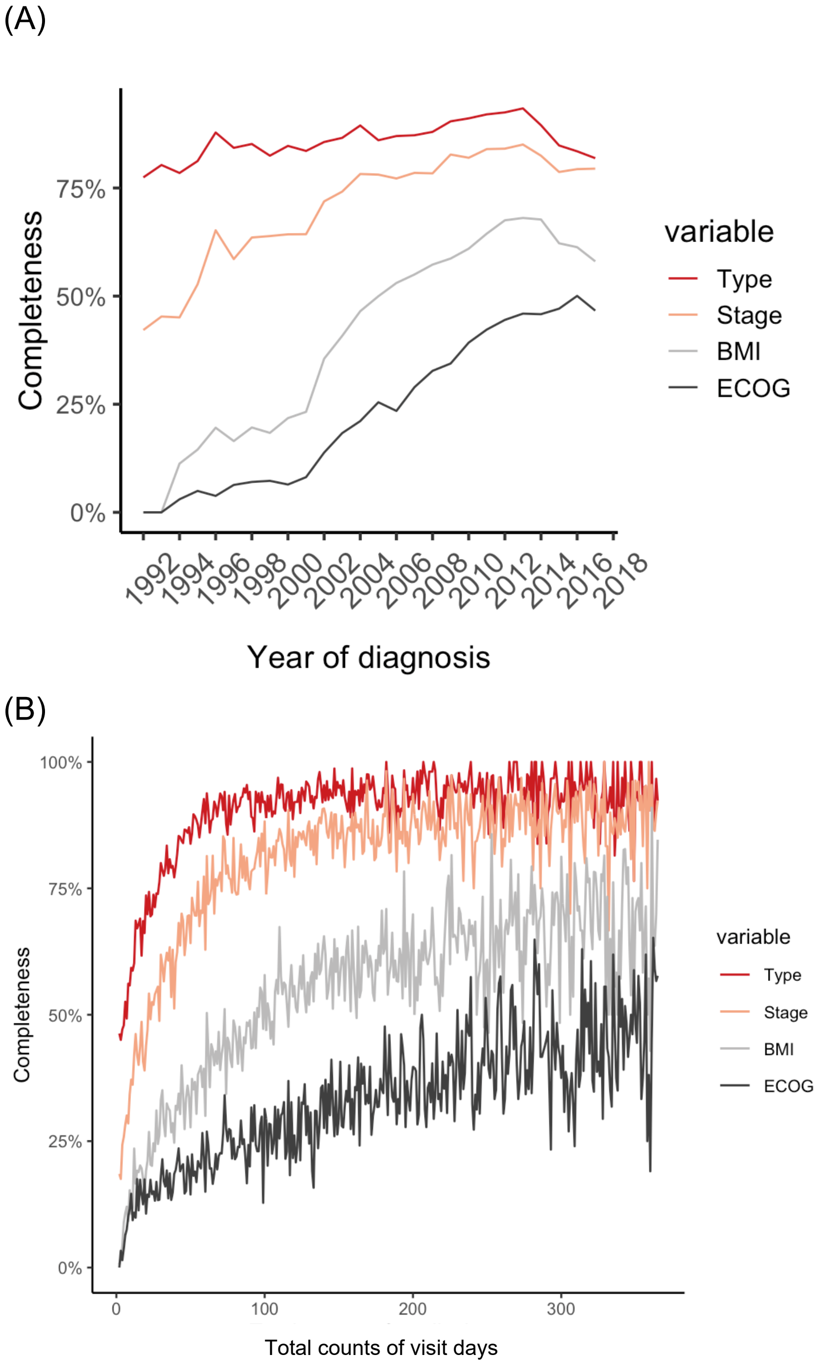

eFigure 2. Completeness of Type, Stage, Body Mass Index, and Eastern Cooperative Oncology Group Performance Status Improvement Over Time With Total Counts of Visit Days

© 2021 Yuan Q et al. *JAMA Network Open.*

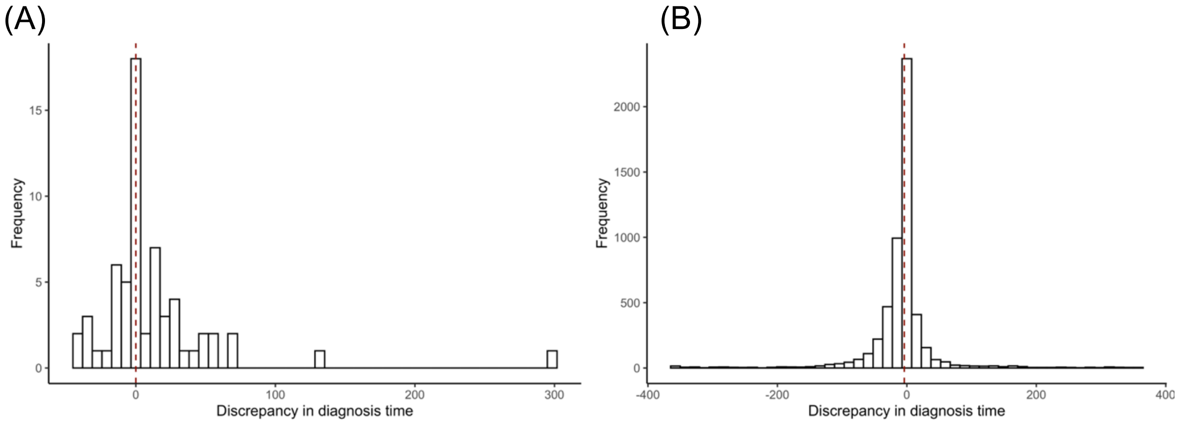

eFigure 3. Histogram of Date Discrepancies for Extracted Diagnosis Date From Electronic Health Records Compared With Record Review and Boston Lung Cancer Study Cohort Diagnosis Date

Positive deflections represent an extracted date that is later than the chart review/BLCS date. Outliers beyond +/- 365 days (1 year) are not shown.

© 2021 Yuan Q et al. *JAMA Network Open.*

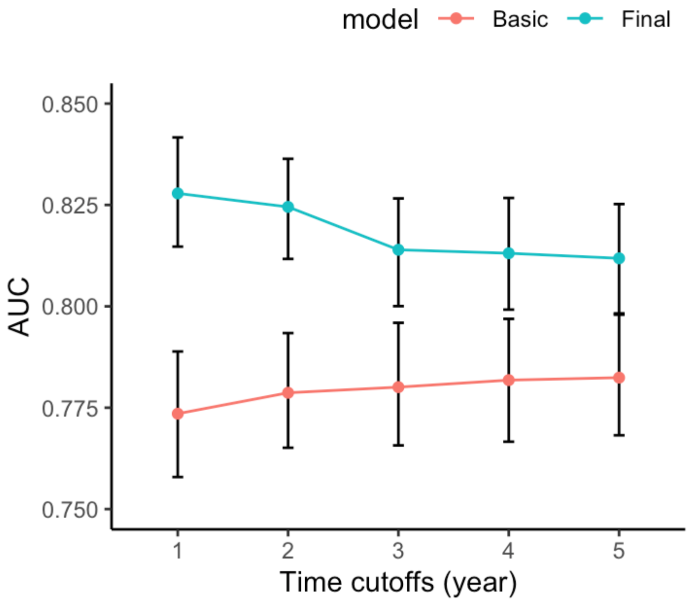

eFigure 4. Time-Dependent Area Under the Receiver Operating Characteristic Curves and 95% CIs for 1 to 5 Years in Testing Set

The final model included age, sex, smoking status, histological type, stage, BMI, albumin, ALP, creatinine, HGB, RDW, WBC, NLR, calcium, and sodium. The basic model included age, sex, histological type, and stage. 1000 bootstrap replicates were performed to estimate the confidence interval of AUC.

© 2021 Yuan Q et al. *JAMA Network Open.*

(A)

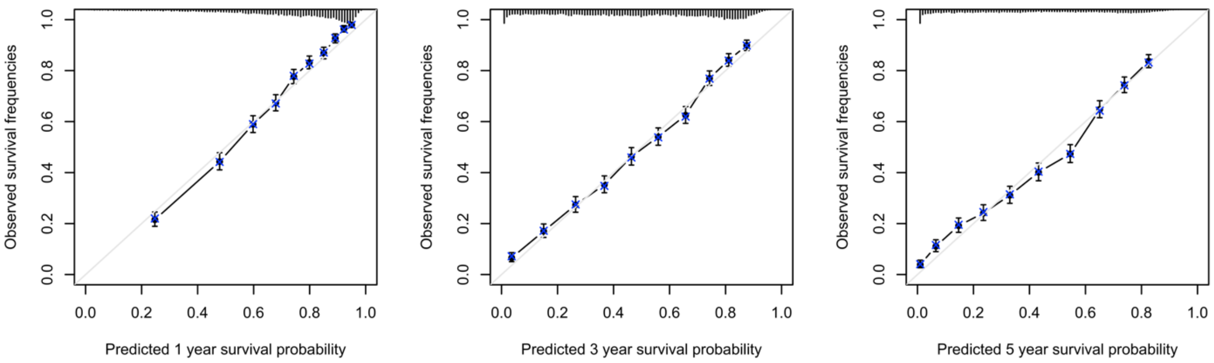

(B(

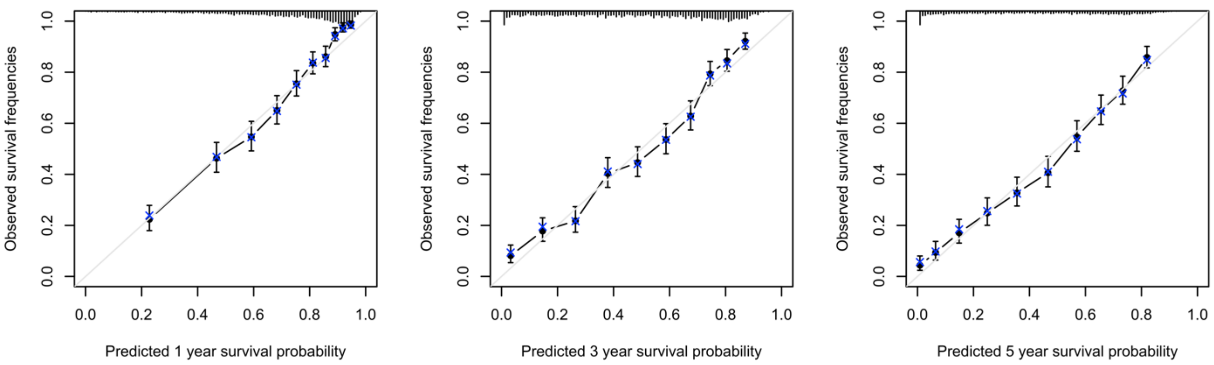

eFigure 5. Calibration Curves Comparing Predicted and Actual Survival Probabilities at 1, 3, and 5 Years for Training and Testing Sets

For each time point, patients were grouped into 10 intervals based on predicted survival probabilities. A plot along the 45-degree line would indicate a perfect calibration model in which the predicted probabilities are identical to the actual outcomes.

© 2021 Yuan Q et al. *JAMA Network Open.*
